# Efficacy of first-line immune checkpoint inhibitors in advanced non-small-cell lung cancer with or without brain metastases: a systematic review and network meta-analysis

**DOI:** 10.3389/fonc.2026.1809450

**Published:** 2026-04-01

**Authors:** Jie Luo, Moxuan Han, Mingwei Sima, Yan Cui, Ziyang Yu, Chang Dong, Kejia Ma, Donghui Yue

**Affiliations:** 1 College of Basic Medicine, Changchun University of Chinese Medicine, Changchun, China; 2 College of Traditional Chinese Medicine, Changchun University of Chinese Medicine, Changchun, China

**Keywords:** brain metastasis, efficacy, immune checkpoint inhibitors, network meta-analysis, non-small cell lung cancer, randomized controlled trial

## Abstract

**Background:**

Immune checkpoint inhibitors (ICIs) are the standard first-line care for advanced non-small cell lung cancer (NSCLC). However, optimal therapeutic choices for patients with brain metastases remain unclear due to a lack of direct comparisons. We conducted a systematic review and Bayesian network meta-analysis to evaluate the comparative efficacy of first-line ICI regimens stratified by brain metastasis status.

**Methods:**

We searched PubMed, Cochrane Library, Embase, and Web of Science for randomized controlled trials (RCTs) of first-line ICI regimens in advanced NSCLC. We performed a Bayesian network meta-analysis to estimate hazard ratios (HRs) and 95% credible intervals (CrIs) for overall survival (OS) and progression-free survival (PFS). Models were stratified by brain metastasis status to rank treatment efficacy using the CINeMA system for quality assessment. The protocol is registered with PROSPERO (CRD420251236228).

**Results:**

We synthesized data from 12 RCTs (n=7, 122) evaluating 14 ICI regimens. In patients with brain metastases, ICI plus chemotherapy significantly improved OS versus chemotherapy alone (HR = 0.57; 95% CrI: 0.45–0.72), while both monotherapy (HR = 0.50; 95% CrI: 0.30–0.85) and combination therapy (HR = 0.42; 95% CrI: 0.31–0.55) prolonged PFS. For patients without brain metastases, both strategies yielded superior OS and PFS. Bayesian ranking indicated that for brain metastases, cemiplimab plus chemotherapy conferred the greatest OS benefit (HR = 0.29; 95% CrI: 0.11–0.76; rank 1 probability: 41.66%). Regarding PFS in this subgroup, sugemalimab plus chemotherapy showed the strongest relative effect (HR = 0.30; 95% CrI: 0.15–0.59), whereas camrelizumab plus chemotherapy achieved the highest probability of ranking first (35.85%). In the non-brain metastasis cohort, pembrolizumab plus chemotherapy (HR = 0.58; 95% CrI: 0.46–0.73) and sintilimab plus chemotherapy (HR = 0.49; 95% CrI: 0.37–0.64) were the leading regimens for OS and PFS, respectively.

**Conclusions:**

ICI plus chemotherapy provides a survival advantage over chemotherapy alone in advanced NSCLC, irrespective of brain metastasis status. Bayesian rankings favor cemiplimab, sugemalimab, or camrelizumab plus chemotherapy for patients with brain metastases, and pembrolizumab or sintilimab plus chemotherapy for those without. These findings, currently limited by low-certainty indirect evidence, warrant validation in adequately powered, head-to-head trials.

**Systematic review registration:**

https://www.crd.york.ac.uk/prospero/, identifier CRD420251236228.

## 1 Introduction

Cancer remains a predominant cause of global mortality, with lung cancer accounting for the highest burden of incidence and death (1). Data from GLOBOCAN 2022 indicates that there are roughly 2.5 million new diagnoses and 1.8 million fatalities attributed to lung cancer globally each year (2). Non-small cell lung cancer (NSCLC) constitutes roughly 85% of these diagnoses, with most patients presenting with advanced or metastatic disease (3). Historically, platinum-based doublet chemotherapy was the cornerstone of first-line treatment (4, 5). Yet, despite improving objective response rates (ORR), chemotherapy offered only modest survival gains, with median overall survival (OS) stagnating at 8–12 months and 5-year survival rates rarely exceeding 5% (6–8). Moreover, the severe toxicity profile of these regimens, including myelosuppression, gastrointestinal toxicity, nephrotoxicity, and peripheral neuropathy, often undermines patient quality of life and treatment adherence (9).

The emergence of immune checkpoint inhibitors (ICIs) has fundamentally reshaped the oncology landscape, offering durable efficacy and manageable safety profiles across a spectrum of malignancies (7, 10, 11). By targeting the PD-1/PD-L1 or CTLA-4 axes, these agents dismantle the immunosuppressive barriers within the tumor microenvironment, effectively reinvigorating T-cell-mediated antitumor immunity (12). This therapeutic potential was famously underscored by the landmark KEYNOTE-024 trial, where pembrolizumab monotherapy delivered a 5-year overall survival rate of 31.9% in patients with high PD-L1 expression (13). Similarly, for non-squamous NSCLC devoid of actionable driver mutations, the addition of pembrolizumab to chemotherapy has translated into substantial OS and progression-free survival (PFS) gains, with 5-year survival rates reaching 19.4%, a figure significantly superior to that achieved by chemotherapy alone (14).

Brain metastases are a frequent site of distant recurrence in advanced NSCLC and are associated with a poor prognosis (15, 16). Approximately 10-25% of patients present with intracranial disease at diagnosis, and up to 50% develop it during the course of their illness (17). The associated morbidity is significant, characterized by seizures, intracranial hypertension, and cognitive or motor deficits (18). However, while systemic therapies for NSCLC have improved substantially, survival gains in this population have lagged behind, highlighting an unmet clinical need.

While guidelines list multiple immune checkpoint inhibitors (ICIs) and chemoimmunotherapy combinations as standard first-line options for NSCLC (19), the best treatment for patients with brain metastases remains undefined. This is largely because direct comparisons between these regimens are rare. Consequently, we conducted a systematic review and Bayesian network meta-analysis to assess comparative survival efficacy. By evaluating specific outcomes based on brain metastasis status, this study aims to define the optimal regimen and guide treatment selection.

## 2 Materials and methods

Reporting follows the PRISMA-NMA extension statement in **Supplementary Table 1** (20), with the protocol registered in PROSPERO (CRD420251236228). We employed a systematic review and Bayesian network meta-analysis to assess the efficacy of first-line immunotherapy. By stratifying outcomes based on the presence or absence of brain metastases, we aimed to define the optimal therapeutic approach. As all data were retrieved from the literature, institutional review board (IRB) approval was not required.

### 2.1 Data sources and search strategy

Two investigators independently performed a comprehensive literature search across PubMed, the Cochrane Library, Embase, and Web of Science for relevant studies published from database inception through November 1, 2025, restricting results to English-language publications. Our strategy integrated Medical Subject Headings (MeSH) with free-text keywords, targeting concepts related to “Non-Small-Cell Lung Cancer”, “Brain Metastases” and “Immune Checkpoint Inhibitors” (including specific PD-1, PD-L1, and CTLA-4 inhibitors such as pembrolizumab, cemiplimab, sintilimab, sugemalimab, nivolumab, tremelimumab, durvalumab, atezolizumab, and serplulimab). Study design filters were applied to isolate randomized controlled trials (RCTs). The full electronic search strings and detailed strategies are tabulated in **Supplementary Table 2**.

### 2.2 Selection criteria

Inclusion Criteria:

1. Enrollment of patients with histologically or cytologically confirmed NSCLC, with explicit stratification by brain metastasis status. Brain metastases were defined as intracranial lesions confirmed via contrast-enhanced MRI, CT imaging (21), or pathological biopsy (22). Patients classified as brain metastasis-free required negative baseline neuroimaging (23).
2. Immune checkpoint inhibitors (ICIs), either as monotherapy or in combination with other agents, administered as first-line therapy.
3. Comparison of ICI-based regimens against standard-of-care alternatives.
4. Reporting of at least one primary efficacy endpoint: Overall Survival (OS), measured as the duration from randomization to all cause mortality; and Progression-Free Survival (PFS), calculated from the date of random assignment until the first documentation of radiographic disease progression or death.
5. Randomized controlled trial (RCT) design.

Exclusion Criteria:

1. Non-randomized studies, including single-arm trials, observational cohorts, reviews, and case reports.
2. Trials that failed to report outcomes stratified by brain metastasis status or lacked relevant subgroup data.
3. Duplicate publications derived from the same patient cohort (in instances of multiple reports from a single trial, we prioritized the most recent or complete dataset).
4. Studies with undefined endpoints or unextractable data.

To identify eligible studies and secure the most current evidence, two reviewers conducted an independent assessment of full text papers after the preliminary title and abstract check.

### 2.3 Data extraction and quality assessment

Data extraction was carried out independently by three authors adhering to PRISMA protocols, with a fourth senior investigator adjudicating any disagreements. From each eligible trial, we retrieved key bibliographic and methodological details-including study identity (name, journal, publication year), registration (NCT number), trial design (phase, randomization ratio), and sample size. We also collated baseline patient characteristics such as age, sex, tumor stage, smoking status, ECOG performance status, and histology, alongside specific dosing protocols for all intervention and control arms. The primary efficacy endpoints extracted were hazard ratios (HRs) and 95% credible intervals (CrIs) for overall survival (OS) and progression-free survival (PFS).

The quality of the studies was evaluated via the Cochrane Risk of Bias Tool 2.0 (RoB 2.0) (24). We systematically evaluated bias across five domains: the randomization process, deviations from intended interventions, missing outcome data, outcome measurement, and selection of reported results. Supplementary appendices were scrutinized to clarify ambiguities where primary texts were insufficient. Studies were categorized into one of three levels: ‘low risk’, ‘some concerns’, or ‘high risk’, according to these standards.

### 2.4 Statistical analysis

Efficacy was assessed via overall survival (OS) and progression-free survival (PFS), with hazard ratios (HRs) and 95% credible intervals (CrIs) used to estimate effect sizes.

Pairwise meta-analyses were conducted within a frequentist framework using RevMan 5.4 to estimate the clinical benefit of first-line immunotherapy versus chemotherapy alone, stratified by brain metastasis status. We conducted subgroup analyses to differentiate outcomes between ICI monotherapy and ICI-chemotherapy combinations. Between-study variation was quantified using Cochrane’s Q test and the I^2^ metric. Data were pooled using a fixed effect model if heterogeneity was low (I² ≤ 50% or P ≥ 0.10) and a random-effects model otherwise. Sensitivity analyses omitting one study at a time were employed to identify sources of instability, and visual assessment of funnel plots was used to check for publication bias. We set the threshold for statistical significance at a bilateral P value of < 0.05.

Using the gemtc and rjags packages within the R environment (version 4.5.2), we conducted a Bayesian network meta-analysis (NMA) to compare the efficacy of first-line ICI regimens, stratifying for brain metastasis status (25). A random-effects model was fitted using four independent Markov chains, each programmed with 20, 000 burn-in iterations followed by 50,000 sampling iterations. MCMC convergence was confirmed using trace plots and Gelman-Rubin statistics. We then employed SUCRA values, calculated from posterior samples, to rank the interventions for each endpoint. Ranking probabilities were obtained via the rank.probability() command, and the resulting hierarchies were displayed in heatmaps using the pheatmap library.

We rigorously evaluated model transitivity by assessing both global and local inconsistency. Global inconsistency was appraised by comparing the Deviance Information Criterion (DIC) of the consistency model against that of the inconsistency model. Local inconsistency was interrogated using the node-splitting method.

### 2.5 Certainty of evidence assessment

We graded the certainty of evidence using the CINeMA framework (26). Starting from a baseline of high certainty for RCTs, we assessed within-study bias by weighting RoB 2.0 ratings according to the network contribution matrix. Evidence could be downgraded across five domains: indirectness, imprecision, heterogeneity, incoherence which refers to the disagreement between direct and indirect evidence, and reporting bias as evaluated via trial registries and small-study effects.We rated each domain as having ‘no concerns’, ‘some concerns’ or ‘major concerns’. Consequently, the overall certainty was downgraded by one or two levels, respectively, yielding a final classification of high, moderate, low, or very low.

## 3 Results

### 3.1 Search results and characteristics of eligible studies

**Figure 1:**
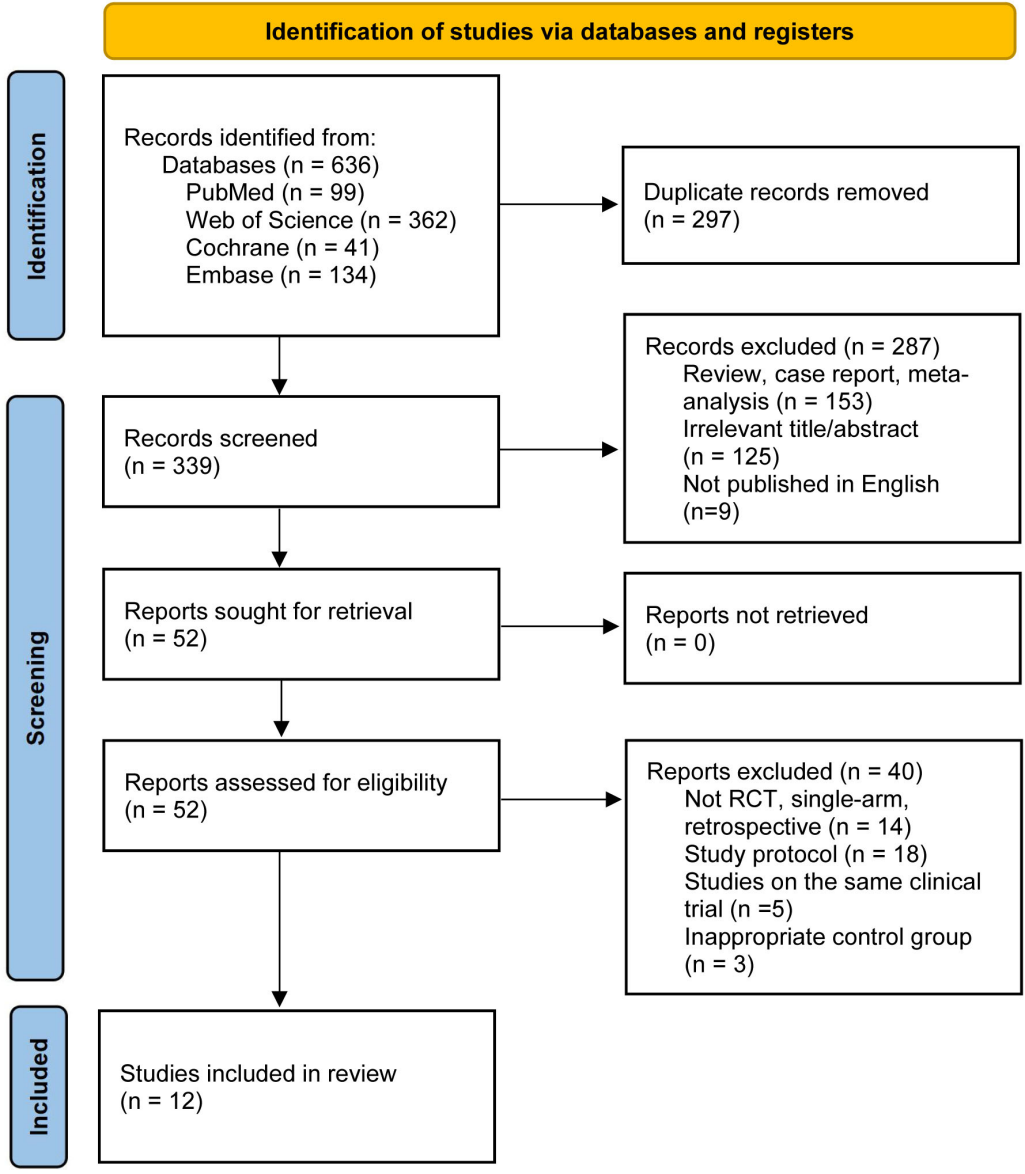
PRISMA flow diagram depicting the literature search and study selection process.

The original search strategy yielded 636 potential articles. Following deduplication and preliminary screening of titles and abstracts, 339 unique citations advanced to full-text eligibility assessment. Of these, 287 were excluded as reviews, case reports, meta-analyses (n = 153), irrelevant studies (n = 125), or non-English publications (n = 9). The remaining 52 articles underwent detailed review, resulting in the exclusion of 40 additional records due to non-randomized/single-arm designs (n = 14), protocol-only publications (n = 18), overlapping patient cohorts (n = 5), or inappropriate comparators (n = 3). Ultimately, 12 RCTs comprising 7,122 patients and 14 distinct treatment nodes were included in the network meta-analysis. The interventions analyzed included chemotherapy alone (chemo), pembrolizumab monotherapy (pembro) or combined with chemotherapy (pembro-chemo), cemiplimab monotherapy (cemip) or combined with chemotherapy (cemip-chemo), and chemotherapy combinations with camrelizumab (camre-chemo), sintilimab (sinti-chemo), sugemalimab (sugem-chemo), durvalumab (durva-chemo), serplulimab (serpl-chemo), or tremelimumab plus durvalumab (treme-durva-chemo), as well as dual immunotherapy regimens involving pembrolizumab plus ipilimumab (pembro-ipi) and nivolumab plus ipilimumab (nivo-ipi), and atezolizumab monotherapy (atezo). The literature search and selection strategy are depicted in the PRISMA flowchart in **Figure 1**; detailed characteristics of the eligible cohorts are listed in **Table 1**.

**Table 1:** Baseline characteristics of studies included in the network meta-analysis.

Study	Source	Registered ID	Sample size	Stage	ECOG PS	Histology	Intervention arm(s)	Control arm
(Phase, design)	(y)	(Randomization)	(Median age/y)	(Male/ female)	Smoking status			
KEYNOTE-024 (27, 28)	J Clin Oncol	NCT02142738	154/151	IV	0:54/53 1:99/98 2:1/0	Squamous	Pembrolizumab 200mg, q3w	Gemcitabine 1250mg/m^2^ + cisplatin 75mg/m^2^/carboplatin AUC 5/6, q3w, for 4–6 cycles; Paclitaxel 200mg/m^2^ + carboplatin AUC 5/6, q3w, for 4–6 cycles;
(open-label, III)	2019	(1:1)	64.5/66	187/118	Never:5/19 Former:115/101 Current:34/31	Non-squamous		Pemetrexed 500mg/m^2^ + cisplatin 75mg/m^2^/carboplatin AUC 5/6, q3w, for 4–6 cycles;
KEYNOTE-189 (29)	Ann Oncol	NCT02578680	410/206	IV	0:185/80 1:221/125 2:1/0	Non-squamous	Pembrolizumab 200mg + pemetrexed 500mg/m² + cisplatin 75mg/m²/carboplatin AUC 5, q3w, for 4 cycles;	Placebo + pemetrexed 500mg/m² + cisplatin 75mg/m²/carboplatin AUC 5, q3w, for 4 cycles;
(double-blind, III)	2021	(2:1)	65/63.5	363/253	Never:48/25 Former or Current:362/181			
EMPOWER-Lung 1 (30)	Lancet	NCT03088540	356/354	IIIB/IIIC/IV	0:96/96 1:260/258	Squamous and non-squamous	Cemiplimab 350mg, q3w	Pemetrexed 500mg/m^2^/paclitaxel 200mg/m^2^+ cisplatin 75mg/m^2^/carboplatin AUC 5/6, q3w, for 4–6 cycles; Gemcitabine 1250mg/m^2^ + cisplatin 100mg/m^2^/carboplatin AUC 5/6, q3w, for 4–6 cycles;
(open-label, III)	2021	(1:1)	63/64	606/104	Former:223/234 Current:133/120			
ORIENT-11 (31)	J THORAC ONCOL	NCT03607539	266/131	IIIB/IIIC/IV	0:75/33 1:191/98	Non-squamous	Sintilimab 200mg + pemetrexed 500mg/m² + cisplatin 75mg/m^2^/carboplatin AUC 5, q3w, for 4 cycles	Placebo + pemetrexed 500mg/m² + cisplatin 75mg/m^2^/carboplatin AUC 5, q3w, for 4 cycles
(double-blind, III)	2021	(2:1)	61/61	303/94	Never:95/44 Former or Current:171/87			
KEYNOTE-598 (32)	J Clin Oncol	NCT03302234	284/284	IV	0:101/104 1:183/180	Squamous and non-squamous	Pembrolizumab 200mg, q3w + ipilimumab 1mg/kg, q6w	Pembrolizumab 200mg, q3w + saline placebo, q6w
(double-blind, III)	2021	(1:1)	64/65	393/174	Never:29/25 Former:197/183 Current:58/76			
CheckMate 227 (33)	J THORAC ONCOL	NCT02477826	583/583	IV	0:204/191 1:377/386 2:2/4 Not reported:0/2	Squamous and non-squamous	Nivolumab 3mg/kg, q2w + ipilimumab 1mg/kg, q6w	Cisplatin 75mg/m^2^/carboplatin AUC 5, q3w
(open-label, III)	2023	(1:1)	60/65	778/388	Never:79/78 Former or Current:497/499 Unknown:7/6			
EMPOWER-Lung 3 (34)	J THORAC ONCOL	NCT03409614	312/154	IIIB/IIIC/IV	0:51/18 1:259/134	Squamous	Cemiplimab 350mg + paclitaxel 200mg/m^2^ + cisplatin 75mg/m^2^/carboplatin AUC 5/6, day 1, q3w, for 4 cycles;	Paclitaxel 200mg/m^2^ + cisplatin 75mg/m^2^/carboplatin AUC 5/6, day 1, q3w, for 4 cycles;
(double-blind, III)	2023	(2:1)	63/63	391/75	Never:43/24 Former:96/55 Current:173/75	Non-squamous	Cemiplimab 350mg + pemetrexed 500mg/m² + cisplatin 75mg/m^2^/carboplatin AUC 5/6, day 1, q3w, for 4 cycles;	Pemetrexed 500mg/m² + cisplatin 75mg/m^2^/carboplatin AUC 5/6, day 1, q3w, for 4 cycles;
IPSOS (35)	Lancet	NCT03191786	302/151	IIIB/IV	0/1:56/19 2:228/116 3:18/16	Squamous and non-squamous	Atezolizumab 1200mg, q3w	Vinorelbine 25–30mg/m^2^/gemcitabine 1250mg/m^2^, q3w, days 1, 8;
(open-label, III)	2023	(2:1)	75/75	328/125	Never:35/20 Former:209/103 Current:58/28			
CameL (36)	J Immunother Cancer	NCT03134872	205/207	IIIB/IIIC/IV	0:48/36 1:157/171	Non-squamous	Camrelizumab 200mg + carboplatin AUC 5 + pemetrexed 500mg/m^2^, q3w, for 4–6 cycles	Carboplatin AUC 5 + pemetrexed 500mg/m^2^, q3w, for 4–6 cycles
(open-label, III)	2024	(1:1)	65/65	295/117	≥20 pack-years: 127/130 <20 pack-years or never:78/77			
ASTRUM-004 (37)	Cancer Cell	NCT04033354	358/179	IIIB/IIIC/IV	0:65/26 1:293/153	Squamous	Serplulimab 4.5mg/kg + nab-paclitaxel 100mg/m² (Days 1, 8, 15)+ carboplatin AUC 5/6, day 1, q3w, for 4–6 cycles	Placebo + nab-paclitaxel 100mg/m²(Days 1, 8, 15)+ carboplatin AUC 5/6, day 1, q3w, for 4–6 cycles
(double-blind, III)	2024	(2:1)	63/63	488/49	Never:50/20 Former:229/122 Current:79/37			
GEMSTONE-302 (38)	Lancet Oncol	NCT03789604	320/159	IV	0:59/25 1:261/134	Squamous	Sugemalimab 1200mg + carboplatin AUC 5 + paclitaxel 175mg/m², day 1, q3w, up to 4 cycles	Placebo + carboplatin AUC 5 + paclitaxel 175mg/m², day 1, q3w, up to 4 cycles
(double-blind, III)	2025	(2:1)	62/64	383/96	Never:88/40 Former or Current:232/119	Non-squamous	Sugemalimab 1200mg + carboplatin AUC 5 + pemetrexed 500mg/m², day 1, q3w, up to 4 cycles	Placebo + carboplatin AUC 5 + pemetrexed 500mg/m², day 1, q3w, up to 4 cycles
POSEIDON (39)	J THORAC ONCOL	NCT03164616	338/338/337	IV	0:110/109/119 1:228/229/218	Squamous	Arm1:Tremelimumab 75mg + Durvalumab 1500mg + platinum-doublet chemotherapy, q3w, up to 4 cycles; Arm2:Durvalumab 1500mg + platinum-doublet chemotherapy, q3w, up to 4 cycles;	Platinum-doublet chemotherapy: Abraxane 100mg/m²(Days 1, 8, 15)+ carboplatin AUC 5/6 day 1, q3w, for 4–6 cycles;Gemcitabine 1000/1250mg/m² (Days 1, 8)+ cisplatin 75mg/m²/carboplatin AUC 5/6, q3w, for 4–6 cycles
(open-label, III)	2025	(1:1:1)	63/64/63	770/243	Never:59/84/79 Former:195/190/191 Current:84/64/66	non-squamous		Abraxane 100mg/m² (Days 1, 8, 15)+ carboplatin AUC 5/6 day 1, q3w, for 4–6 cycles; Pemetrexed 500mg/m² + cisplatin 75mg/m²/carboplatin AUC 5/6, day 1, q3w, for 4–6 cycles;

According to the RoB 2.0 evaluation, half of the 12 studies achieved a ‘low risk’ rating. The others were noted for ‘some concerns’, ‘ mainly driven by the lack of masking in open-label protocols. However, the use of hard survival endpoints largely insulates the analysis from the performance and detection biases typical of unblinded designs. Thus, we regard the influence on our results as minimal. Overall, the methodological robustness of these RCTs ensures the validity of our findings, as detailed in **Figure 2**.

**Figure 2:**
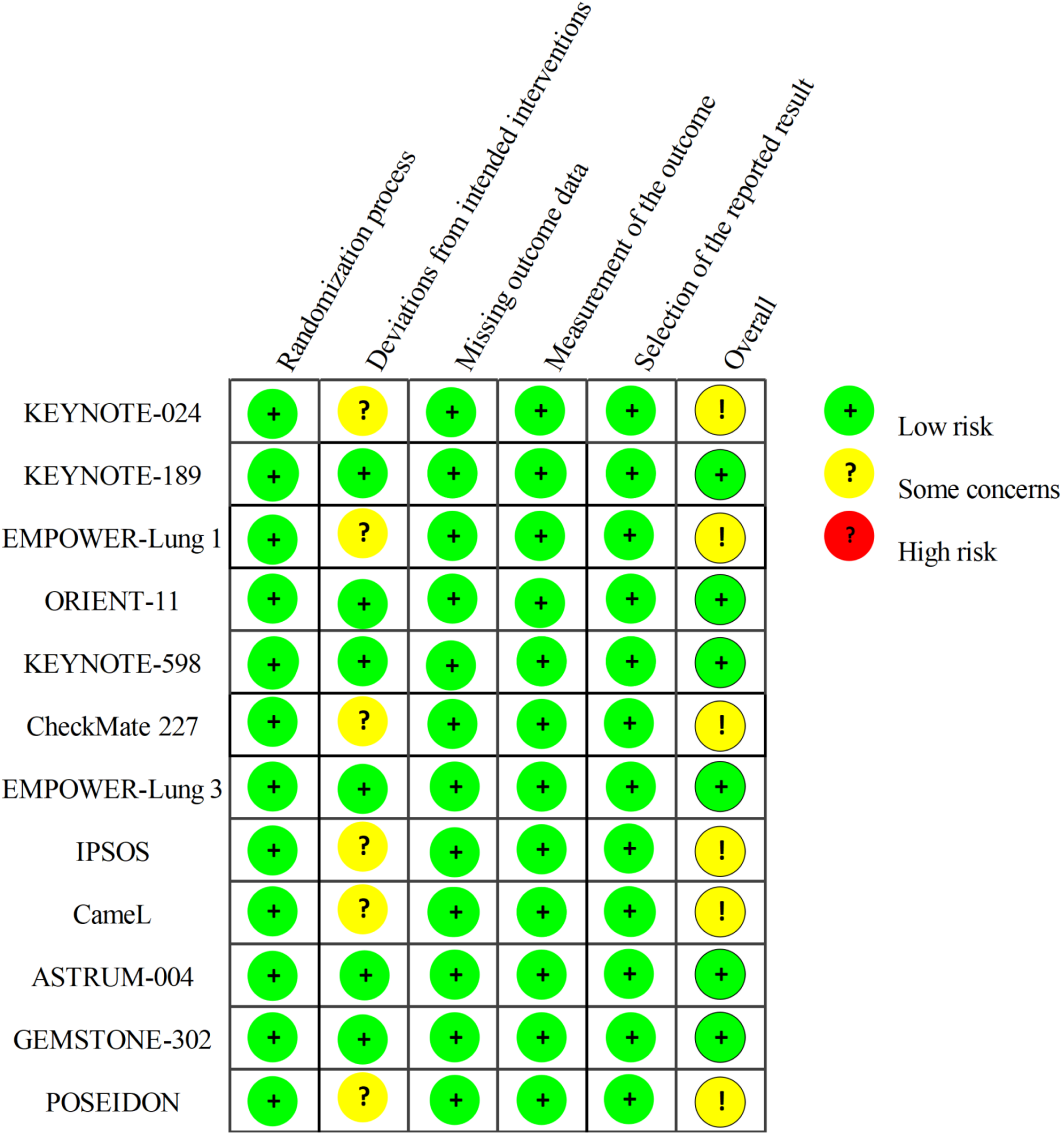
Overview of methodological quality based on Cochrane’s RoB 2.0 criteria. Studies are graded as low risk, some concerns, or high risk within each specific bias domain.

### 3.2 Pairwise meta-analysis

#### 3.2.1 Pairwise meta-analysis of overall survival

**Figure 3:**
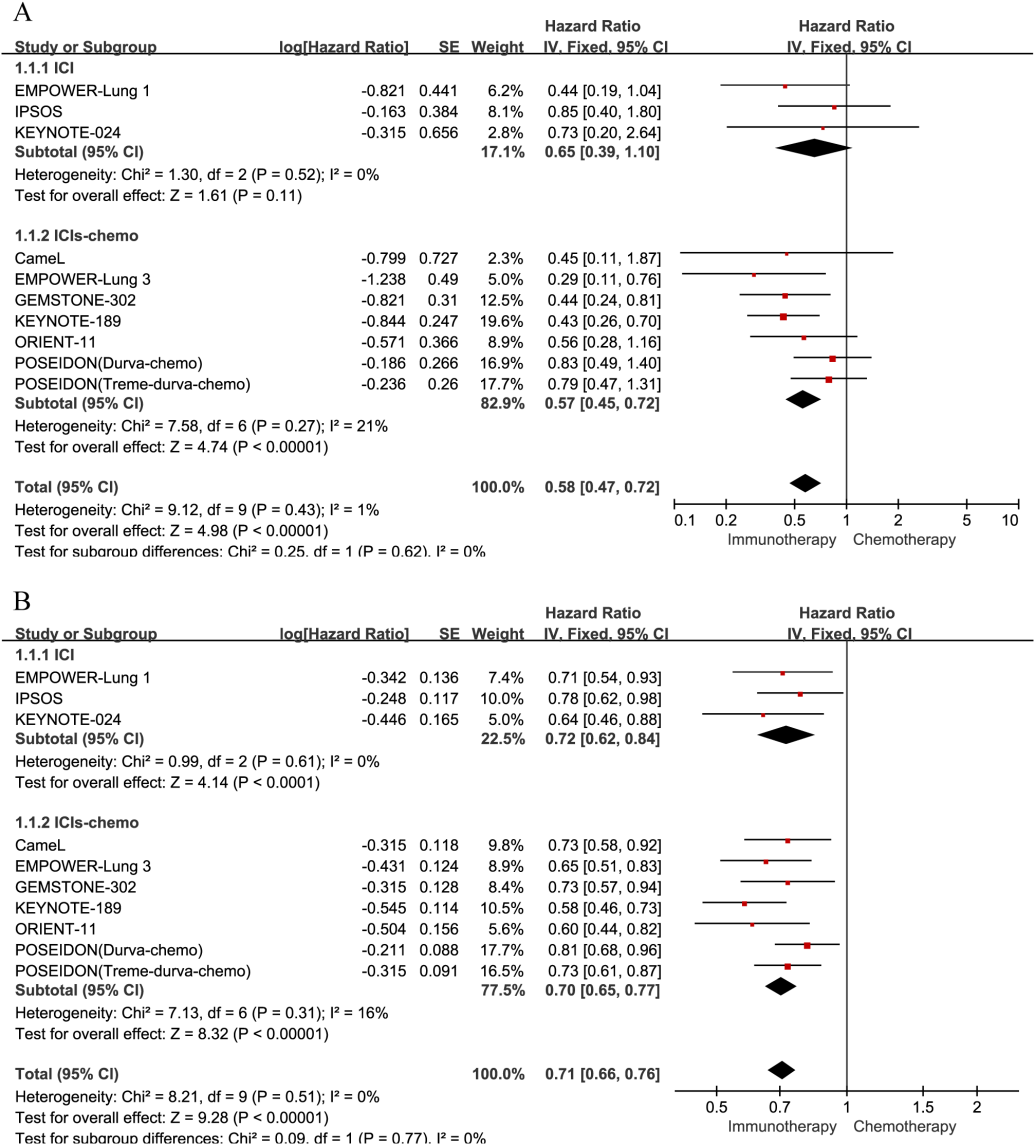
Forest plots illustrating the pairwise meta-analysis of overall survival (OS). The efficacy of first-line immunotherapy versus chemotherapy is compared in patients with NSCLC stratified by brain metastasis status: **(A)** patients with brain metastases; **(B)** patients without brain metastases.

A total of nine studies contributed OS data for the cohort with brain metastases. Given the negligible heterogeneity (P = 0.43, I² = 1%), a fixed-effects model was deemed appropriate for the analysis in **Figure 3A**.In this cohort, ICI monotherapy demonstrated a trend toward improved survival compared with chemotherapy alone (HR = 0.65, 95% CrI: 0.39–1.10), though this failed to reach statistical significance. In contrast, the combination of ICI plus chemotherapy conferred a significant survival advantage over chemotherapy alone (HR = 0.57, 95% CrI: 0.45–0.72).

For patients without brain metastases, nine studies reported extractable OS outcomes. Heterogeneity was virtually absent (P = 0.51, I² = 0%), supporting the application of a fixed-effects model in **Figure 3B**. Compared with chemotherapy alone, both ICI monotherapy (HR = 0.72, 95% CrI: 0.62–0.84) and ICI-chemotherapy combinations (HR = 0.70, 95% CrI: 0.65–0.77) were associated with significantly prolonged overall survival.

#### 3.2.2 Pairwise meta-analysis of progression-free survival

**Figure 4:**
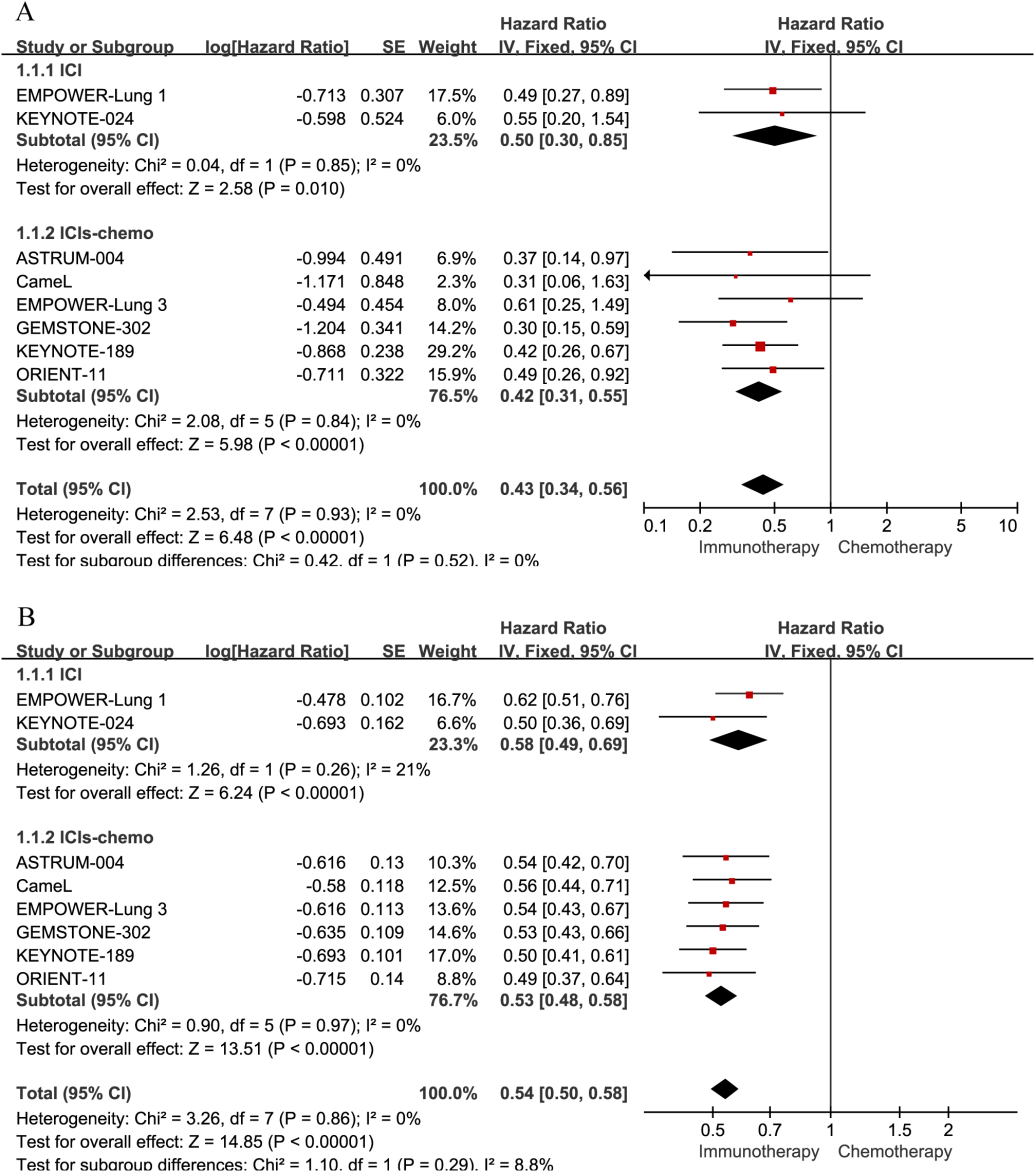
Forest plots illustrating the pairwise meta-analysis of progression-free survival (PFS). Comparisons of first-line immunotherapy versus chemotherapy are shown for patients with NSCLC: **(A)** patients with brain metastases; **(B)** patients without brain metastases.

With eight studies reporting PFS for the brain metastases subgroup showing no evident heterogeneity(P = 0.93, I² = 0%), we employed a fixed-effects model in **Figure 4A**. The analysis revealed that both ICI monotherapy (HR = 0.50, 95% CrI: 0.30–0.85) and ICI plus chemotherapy (HR = 0.42, 95% CrI: 0.31–0.55) yielded significant PFS benefits relative to chemotherapy alone.

Similarly, among patients without brain metastases (eight studies), heterogeneity was negligible (P = 0.86, I² = 0%), warranting a fixed-effects approach in **Figure 4B**. Both ICI monotherapy (HR = 0.58, 95% CrI: 0.49–0.69) and ICI plus chemotherapy (HR = 0.53, 95% CrI: 0.48–0.58) significantly reduced the risk of disease progression compared with chemotherapy alone.

### 3.3 Network meta-analysis

**Figure 5:**
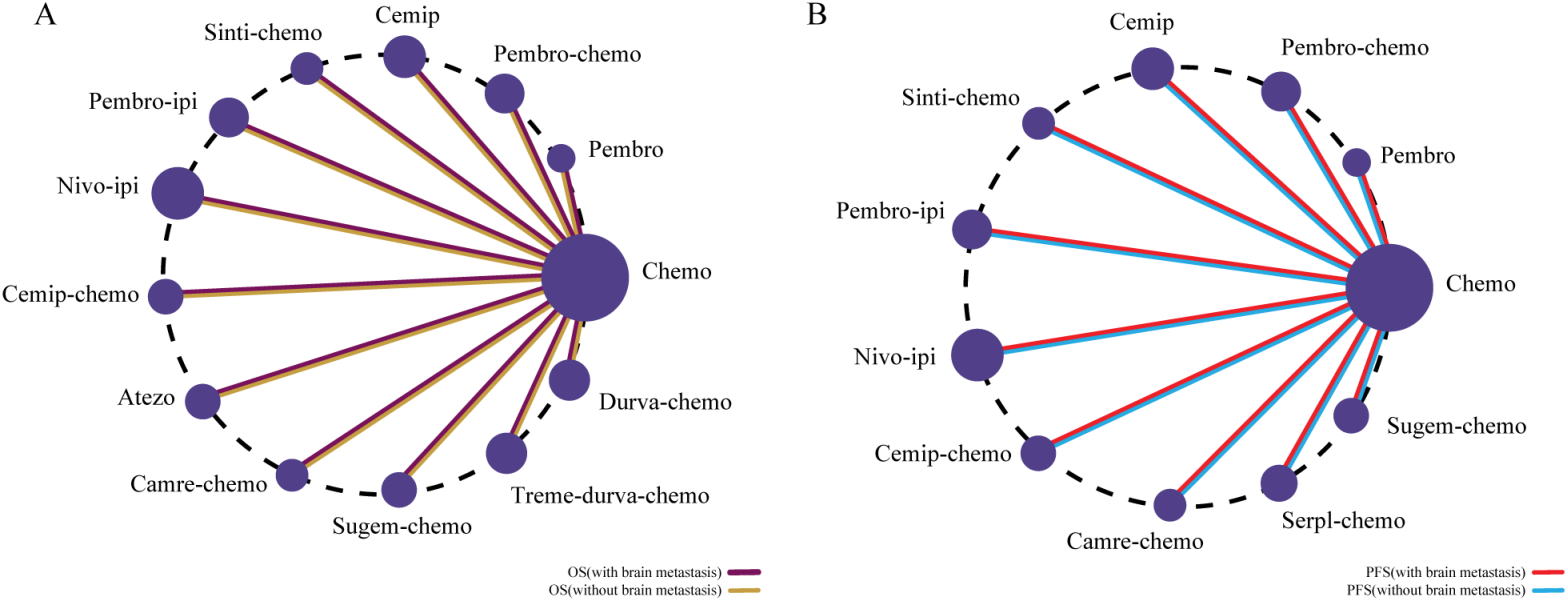
Network plots of comparisons for first-line immunotherapeutic regimens in patients with NSCLC with and without brain metastases. **(A)** Network plot for overall survival (OS); **(B)** Network plot for progression-free survival (PFS).

We constructed Bayesian network meta-analysis models to evaluate OS and PFS. The evidence network for OS incorporated 13 first-line immunotherapeutic interventions across the entire NSCLC population stratified by brain metastasis status as depicted in **Figure 5A**, while the PFS network comprised 11 distinct regimens as presented in **Figure 5B**.

#### 3.3.1 Comparative efficacy for overall survival

**Figure 6:**
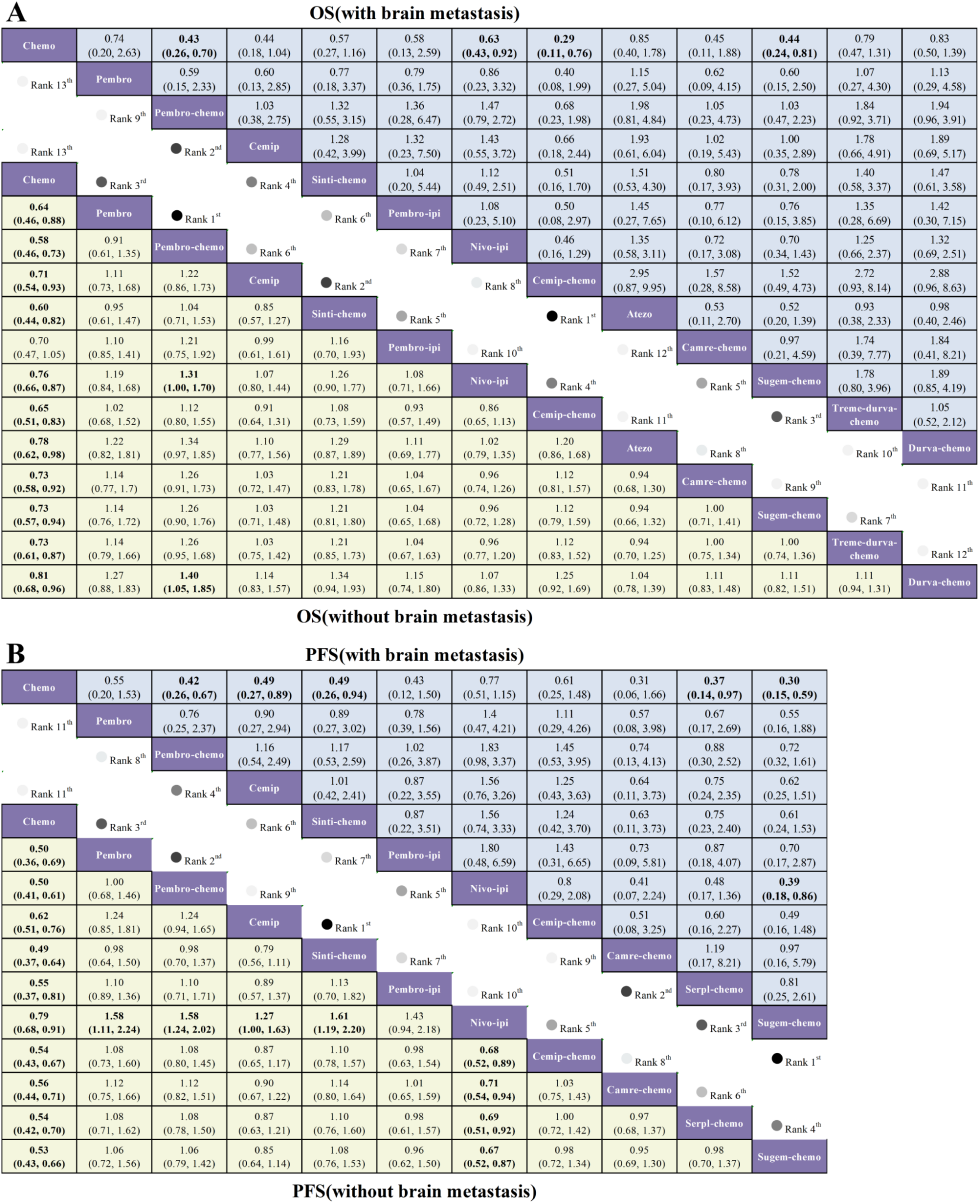
League tables of relative efficacy for first-line immunotherapies in NSCLC. Results are stratified by brain metastasis status for **(A)** overall survival (OS) and **(B)** progression-free survival (PFS). Comparisons for the brain metastases subgroup are located in the upper-right (blue) section, whereas non-brain metastases outcomes appear in the lower-left (yellow). Values represent Hazard Ratios (HRs) and 95% Credible Intervals (CrIs), with estimates below 1.00 indicating superiority of the column regimen.

Among patients with brain metastases in **Figure 6A**, several ICI-chemotherapy combinations yielded significant survival benefits relative to chemotherapy alone. Specifically, cemip-chemo (HR = 0.29, 95% CrI: 0.11–0.76), pembro-chemo (HR = 0.43, 95% CrI: 0.26–0.70), and sugem-chemo (HR = 0.44, 95% CrI: 0.24–0.81) all significantly prolonged OS. In direct comparisons between active agents, sugem-chemo exhibited survival efficacy comparable to that of cemiplimab monotherapy (HR = 1.00, 95% CrI: 0.35–2.89).

In the cohort without brain metastases in **Figure 6A**, pembro-chemo (HR = 0.58, 95% CrI: 0.46–0.73), sinti-chemo (HR = 0.60, 95% CrI: 0.44–0.82), and pembrolizumab monotherapy (HR = 0.64, 95% CrI: 0.46–0.88) all significantly improved OS compared with chemotherapy alone. Additionally, pembrolizumab monotherapy demonstrated survival outcomes comparable to those of cemip-chemo (HR = 1.02, 95% CrI: 0.68–1.52).

#### 3.3.2 Comparative efficacy for progression-free survival

For patients with brain metastases in **Figure 6B**, ICI-chemotherapy combinations demonstrated superior efficacy in controlling disease progression compared with chemotherapy alone. Both sugem-chemo (HR = 0.30, 95% CrI: 0.15–0.59) and serpl-chemo (HR = 0.37, 95% CrI: 0.14–0.97) conferred a significant PFS benefit. Camre-chemo also showed a strong trend toward prolonged PFS (HR = 0.31, 95% CrI: 0.06–1.66), although this did not reach statistical significance due to wide credible intervals.

Among patients without brain metastases in **Figure 6B**, sinti-chemo (HR = 0.49, 95% CrI: 0.37–0.64), pembro-chemo (HR = 0.50, 95% CrI: 0.41–0.61), and pembrolizumab monotherapy (HR = 0.50, 95% CrI: 0.36–0.69) all significantly reduced the risk of disease progression relative to chemotherapy alone. No significant difference in efficacy was observed between pembrolizumab monotherapy and sugem-chemo (HR = 1.06, 95% CrI: 0.72–1.56).

### 3.4 Probabilistic ranking of regimens

**Figure 7:**
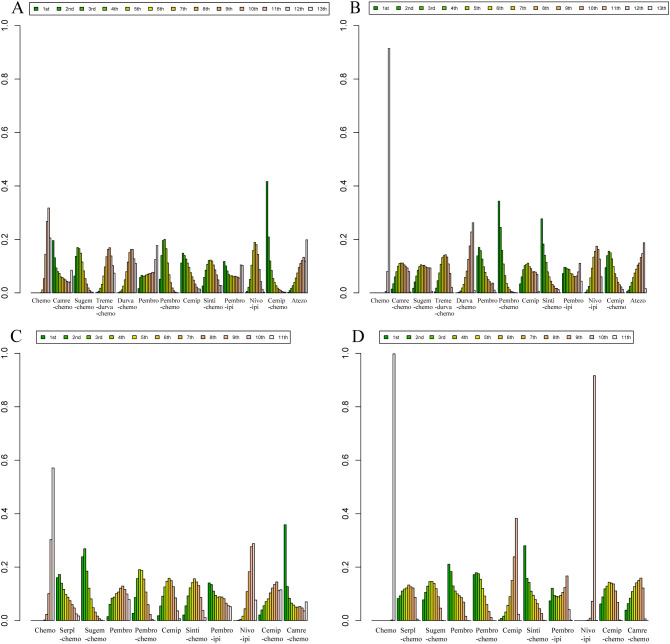
Rank probability plots for first-line treatments. Data are organized by endpoint and subgroup: OS for patients with **(A)** and without **(B)** brain metastases; PFS for patients with **(C)** and without **(D)** brain metastases.

Bayesian ranking profiles were used to identify the optimal first-line regimens, stratified by brain metastasis status, as shown in **Figure 7**.

For overall survival, cemip-chemo exhibited the highest probability of being the best treatment (rank 1 probability: 41.66%) in the brain metastasis cohort. In contrast, among patients without brain metastases, pembro-chemo was identified as the top-ranked regimen (rank 1 probability: 34.31%). Both combinations conferred significant survival advantages within their respective subpopulations.

With respect to progression-free survival, camre-chemo held the highest probability of ranking first (35.85%) for patients with brain metastases, followed by sugem-chemo (26.87%). Conversely, in the non-brain metastasis cohort, sinti-chemo emerged as the most likely optimal strategy (rank 1 probability: 28.00%), with pembrolizumab monotherapy ranking second (18.36%).

### 3.5 Heterogeneity and inconsistency

Inspection of diagnostic trace plots and the Gelman-Rubin statistic verified that the model converged satisfactorily, showing well-mixed Markov Chain Monte Carlo (MCMC) sampling in **Supplementary Figures 1**-**8**. To test the transitivity assumption, we assessed global inconsistency by comparing the Deviance Information Criterion (DIC) of the consistency and inconsistency models. The difference in DIC values remained consistently below 3 points across all outcomes in **Table 2**, indicating an absence of significant global inconsistency. This high concordance between direct and indirect evidence supports the validity of the network estimates.

**Table 2:** Evaluation of DIC values in consistency and inconsistency models.

Outcome	Subgroup	DIC (consistency)	DIC (inconsistency)	Difference
OS	with brain metastasis	23.90	23.99	0.09
	without brain metastasis	24.10	23.98	0.12
PFS	with brain metastasis	20.07	19.93	0.14
	without brain metastasis	20.11	19.98	0.13

**Figure 8:**
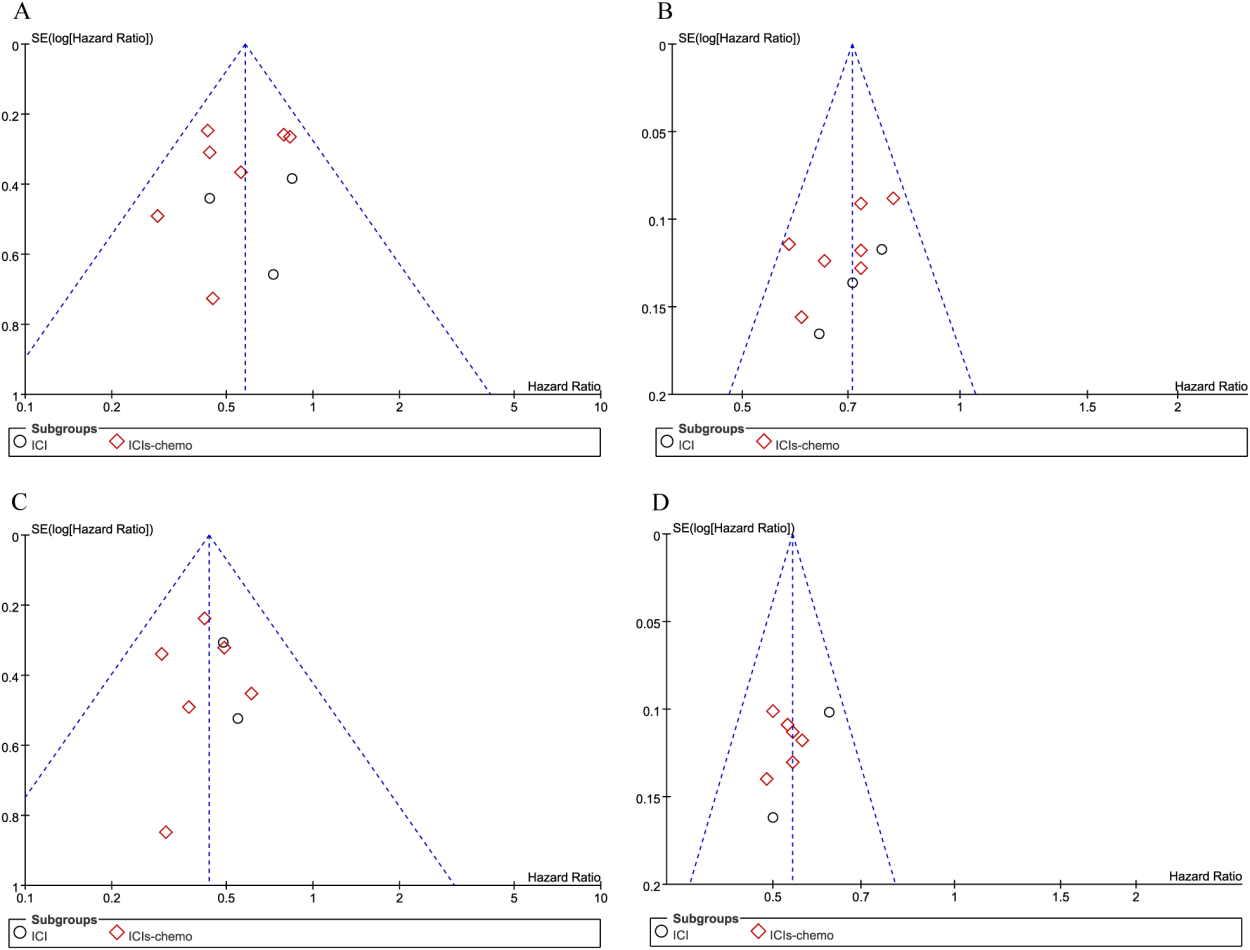
Funnel plots for publication bias. Data are separated by endpoint and subgroup: OS for patients with **(A)** and without **(B)** brain metastases; PFS for patients with **(C)** and without **(D)** brain metastases.

The stability of our primary estimates was validated through leave-one-out sensitivity analyses, which showed negligible deviation in the pooled effect sizes. Regarding publication bias, the funnel plots for both OS and PFS exhibited broad symmetry in **Figure 8**. Most data points fell within the expected confidence boundaries, indicating minimal risk of small-study effects or selective reporting.

### 3.6 Certainty of evidence

Evidence quality throughout the network was evaluated via the CINeMA approach. Overall, the quality of evidence was limited, with all estimates classified as either “low” or “very low” certainty. Specifically, for OS in patients with brain metastases, 37 of 78 comparisons (47.44%) were rated as “low” and 41 (52.56%) as “very low.” In the non-brain metastasis cohort, 26 of 78 OS comparisons (33.33%) were graded as “low”, while the remaining 52 (66.67%) were “very low”. Regarding PFS, evidence for patients with brain metastases was rated as “low” in 42 of 55 comparisons (76.36%) and “very low” in 13 (23.64%). Similarly, for patients without brain metastases, 34 of 55 PFS comparisons (61.82%) were graded as “low” and 21 (38.18%) as “very low”. Full details of the CINeMA assessment are tabulated in **Supplementary Tables 3**-**6**.

## 4 Discussion

This study represents the first Bayesian network meta-analysis to systematically evaluate and rank first-line immunotherapeutic strategies for advanced NSCLC, explicitly stratified by brain metastasis status. By synthesizing direct and indirect evidence from high-quality RCTs, we addressed the lack of head-to-head comparisons in this domain. Compared to chemotherapy alone, superior outcomes regarding both survival and disease progression were observed with select ICI-chemotherapy combinations in our analysis. However, these findings warrant cautious interpretation. Network estimates are inherently sensitive to heterogeneity in trial populations, control arms, and eligibility criteria. Moreover, given the generally low certainty of the available evidence, our rankings should be viewed as “probabilistic hierarchies of relative benefit” rather than definitive declarations of clinical superiority. Nonetheless, our data offer valuable insights for the clinical management of patients matching the trial cohorts, most notably those with stable or asymptomatic brain metastases. Additionally, these outcomes may guide the strategic planning of upcoming clinical trials. Our key findings are summarized as follows:

1. In the brain metastasis subpopulation, ICI-chemotherapy combinations consistently conferred significant OS and PFS benefits over chemotherapy alone, supporting their role as the preferred first-line strategy. Conversely, while ICI monotherapy improved PFS, it failed to demonstrate a statistically significant OS advantage in this high-risk group.
2. In the non-brain metastases subgroup, ICI monotherapy and chemo-immunotherapy combinations both demonstrated superior efficacy in OS and PFS relative to chemotherapy alone.
3. According to Bayesian ranking probabilities for patients with brain metastases, cemip-chemo exhibited the highest likelihood of maximizing OS, while camre-chemo and sugem-chemo emerged as the most effective regimens for prolonging PFS.
4. Regarding the population without brain metastases, ranking probabilities favored pembro-chemo for OS and sinti-chemo for PFS as the most effective interventions.

This analysis establishes that chemo-immunotherapy outperforms chemotherapy alone in advanced NSCLC, irrespective of the presence of brain metastases. The combination leverages distinct but complementary pathways: ICIs antagonize PD-1/PD-L1 checkpoint signaling to reinvigorate anti-tumor immunity (40), while chemotherapy induces immunogenic cell death, shedding antigens that render the tumor more visible to the immune system (41). This dual attack addresses key failure modes of monotherapy, including chemotherapy resistance and the limited activity of ICIs in low-antigenicity tumors (42, 43). By coupling the antigen-priming effects of cytotoxic agents with the checkpoint-release mechanism of ICIs, the regimen achieves a synergy that drives consistent survival benefits across all patient subgroups (44).

Among patients with brain metastases, cemip-chemo conferred the greatest overall survival benefit. This efficacy may derive from the biological properties of cemiplimab, a high-affinity IgG4 monoclonal antibody known to induce potent, durable systemic immunity (45). While the blood-brain barrier (BBB) typically restricts macromolecular transport, the inflammatory microenvironment of metastatic lesions often disrupts BBB integrity, thereby permitting antibody penetration into the central nervous system (46). In contrast, camre-chemo and sugem-chemo emerged as the superior regimens for progression-free survival (PFS), suggesting superior early intracranial control. Camrelizumab accelerates T-cell activation and trafficking to the tumor bed; when combined with the cytoreductive impact of chemotherapy, this synergy can drive rapid regression of intracranial disease (47). Uniquely, sugemalimab engages Fcγ receptors to trigger antibody-dependent cellular phagocytosis (ADCP) of PD-L1-expressing cells by macrophages (48, 49). Notably, while the pronounced PFS advantages of camrelizumab and sugemalimab indicate strong initial disease suppression, extended follow-up is necessary to confirm whether this early control translates into a sustained survival benefit.

Among patients free of brain metastases, pembro-chemo emerged as the premier regimen for OS, while sinti-chemo yielded the superior PFS outcomes. From a mechanistic standpoint, pembrolizumab blocks the interaction between PD-1 and PD-L1, thereby restoring T-cell activity. This effect is potentiated by chemotherapy, which induces immunogenic cell death and antigen shedding, thereby establishing durable immunological memory (50, 51). In parallel, sintilimab is distinguished by its high binding affinity for PD-1, facilitating rapid checkpoint saturation that accelerates the activation and expansion of tumor-infiltrating lymphocytes (TILs) (52). Crucially, the absence of central nervous system involvement implies a distinct clinical phenotype; these patients typically exhibit a lower systemic tumor burden and an immune microenvironment that is inherently more amenable to checkpoint blockade (53).

While several prior meta-analyses have explored the role of immunotherapy in NSCLC across populations with and without brain metastases, pertinent knowledge gaps remain. Consistent with Brown et al. (54), our findings confirm that combining ICIs with chemotherapy yields superior efficacy over chemotherapy alone, irrespective of intracranial disease status. However, previous studies stopped short of delineating the comparative efficacy of individual regimens. This study is the first to employ network meta-analysis to rank 14 immunotherapeutic strategies, determining the optimal first-line treatments based on brain metastasis status. Notably, while Lyu et al. positioned pembrolizumab as the preferred agent for patients with brain metastases (55), their conclusions were confounded by the pooling of RCTs with retrospective data, a methodology prone to significant heterogeneity and selection bias. In contrast, our analysis was strictly confined to high-quality RCTs, with independent evaluations of brain metastasis subgroups. This rigorous design minimizes confounding, thereby enhancing the internal validity and clinical robustness of our recommendations.

Intriguingly, our analysis reveals that the optimal first-line therapeutic strategy appears contingent upon brain metastasis status. In patients without intracranial disease, pembrolizumab plus chemotherapy conferred a significant survival advantage, a finding that aligns with the consensus of current clinical guidelines and major pivotal trials. By contrast, within the brain metastasis cohort, cemiplimab plus chemotherapy emerged as the leading candidate. This divergence in efficacy profiles implies a potential biological nuance-perhaps involving superior blood-brain barrier penetration or favorable modulation of the intracranial immune microenvironment specific to the cemiplimab-chemotherapy combination. While hypothetical, this observation warrants a dedicated mechanistic investigation. Ultimately, these data underscore the critical need for precise risk stratification and the adoption of tailored, organ-specific therapeutic strategies in the management of metastatic NSCLC.

This study represents the first Bayesian network meta-analysis to systematically appraise and rank first-line immunotherapeutic regimens for advanced NSCLC, explicitly stratified by brain metastasis status. By restricting our analysis to 12 high-quality RCTs, we maximized internal validity while minimizing selection bias and confounding. Leveraging SUCRA probability rankings to bridge the gap left by missing head-to-head trials, we identified distinct optimal strategies for specific patient subgroups. Our data indicate that cemip-chemo, camre-chemo, and sugem-chemo should be prioritized in the management of patients with brain metastases, whereas pembro-chemo and sinti-chemo emerge as the superior choices for those without intracranial involvement. These findings offer clinicians granular, evidence-based decision support tailored to baseline characteristics, effectively complementing current NCCN guidelines for the brain-metastatic subpopulation. However, as these recommendations are derived from indirect comparisons, they should be viewed as hypothesis-generating. Future research must prioritize dedicated head-to-head trials in this high-risk cohort and investigate novel combinatorial approaches-such as immunotherapy plus anti-angiogenic agents-to further expand the therapeutic armamentarium against brain metastases.

The predominantly low to very low certainty of evidence assessed by CINeMA reflects inherent limitations in the source literature. Specifically, estimates were downgraded for imprecision-due to small brain metastasis cohorts and wide confidence intervals-and for indirectness, owing to the lack of head-to-head comparisons. Despite this, our study offers the best available comparative framework for this specific population.

Our study has several limitations that merit consideration. First, the evidence base for certain interventions was limited to a single RCT, making the resultant rankings sensitive to the outcomes of isolated trials. Consequently, these specific findings should be considered provisional pending validation in future confirmatory studies. Second, the reliance on indirect comparisons-necessitated by the scarcity of head-to-head trials-inherently constrains the certainty of relative efficacy estimates. Third, potential heterogeneity in baseline characteristics, particularly the definition and management of brain metastases, may impact generalizability. Notably, pivotal trials such as KEYNOTE-024 and IPSOS predominantly enrolled patients with treated, asymptomatic intracranial disease; thus, our results may not fully extrapolate to populations with active or untreated metastases. Notwithstanding these constraints, this analysis represents the most comprehensive and contemporary synthesis of the available evidence, providing a critical reference for clinical decision-making in this therapeutically challenging subpopulation.

## Data Availability

The original contributions presented in the study are included in the article/**Supplementary Material**. Further inquiries can be directed to the corresponding author.
