# Supplementary material for: Efficacy of first-line immune checkpoint inhibitors in advanced non-small-cell lung cancer with or without brain metastases: a systematic review and network meta-analysis
Source: Front Oncol. 2026 Apr 1;16:1809450. doi: 10.3389/fonc.2026.1809450 (PMC13079038; doi:10.3389/fonc.2026.1809450)
Supplement: Supplementary file 1 [file DataSheet1.docx]

Supplementary Material

| **Table of Contents** | | |
| --- | --- | --- |
| **Title** | **Content** | **page** |
| Table S1 | PRISMA NMA Checklist of Items to Include When Reporting a Systematic Review Involving a Network Meta-analysis | 1-5 |
| Table S2 | Literature Search Strategy | 5-8 |
| Table S3 | Certainty of evidence for overall survival in NSCLC patients with brain metastases assessed by CINeMA. | 33-34 |
| Table S4 | Certainty of evidence for overall survival in NSCLC patients without brain metastases assessed by CINeMA. | 35-36 |
| Table S5 | Certainty of evidence for progression-free survival in NSCLC patients with brain metastases assessed by CINeMA. | 37-38 |
| Table S6 | Certainty of evidence for progression-free survival in NSCLC patients without brain metastases assessed by CINeMA. | 39-40 |
| Figure S1 | Convergence diagnostics for the overall survival (OS) model in the brain metastasis subgroup. | 9-10 |
| Figure S2 | Trace and density plots for the overall survival (OS) model in the brain metastasis subgroup. | 11-14 |
| Figure S3 | Convergence diagnostics for the overall survival (OS) model in the non-brain metastasis subgroup. | 15-16 |
| Figure S4 | Trace and density plots for the overall survival (OS) model in the non-brain metastasis subgroup. | 17-20 |
| Figure S5 | Convergence diagnostics for the progression-free survival (PFS) model in the brain metastasis subgroup. | 21-22 |
| Figure S6 | Trace and density plots for the progression-free survival (PFS) model in the brain metastasis subgroup. | 23-26 |
| Figure S7 | Convergence diagnostics for the progression-free survival (PFS) model in the non-brain metastasis subgroup. | 27-28 |
| Figure S8 | Trace and density plots for the progression-free survival (PFS) model in the non-brain metastasis subgroup. | 29-32 |

Supplementary Table 1. PRISMA NMA Checklist of Items to Include When Reporting a Systematic Review Involving a Network Meta-analysis

| **Section/Topic** | **Item #** | **Checklist Item** | **Reported on Page #** |
| --- | --- | --- | --- |
| **TITLE** |  |  |  |
| Title | 1 | Identify the report as a systematic review *incorporating*  *anetwork meta-analysis (or related form of meta-analysis).* | **1** |
|  |  |  |  |
| **ABSTRACT** |  |  |  |
| Structured summary | 2 | Provide a structured summary including, as applicable:  **Background:** main objectives  **Methods:** data sources; study eligibility criteria, participants, and interventions; study appraisal; and *synthesis methods, such as network meta-analysis.*  **Results:** number of studies and participants identified; summary estimates with corresponding confidence/credible intervals; *treatment rankings may also be discussed. Authors may choose to summarize pairwise comparisons against a chosen treatment included in their analyses for brevity.*  **Discussion/Conclusions:** limitations; conclusions and implications of findings.  **Other:** systematic review registration number with registry name. | 1-2 |
|  |  |  |  |
| **INTRODUCTION** |  |  |  |
| Rationale | 3 | Describe the rationale for the review in the context of what is already known*, including mention of why a network meta-analysis has been conducted.* | **2** |
| Objectives | 4 | Provide an explicit statement of questions being addressed, with reference to participants, interventions, comparisons, outcomes, and study design (PICOS). | 2 |
|  |  |  |  |
| **METHODS** |  |  |  |
| Protocol and registration | 5 | Indicate whether a review protocol exists and if and where it can be accessed (e.g., Web address); and, if available, provide registration information, including registration number. | 2 |
| Eligibility criteria | 6 | Specify study characteristics (e.g., PICOS, length of follow-up) and report characteristics (e.g., years considered, language, publication status) used as criteria for eligibility, giving rationale. *Clearly describe eligible treatments included in the treatment network, and note whether any have been clustered or merged into the same node (with justification).* | 2-3 |
| Information sources | 7 | Describe all information sources (e.g., databases with dates of coverage, contact with study authors to identify additional studies) in the search and date last searched. | 2 |
| Search | 8 | Present full electronic search strategy for at least one database, including any limits used, such that it could be repeated. | 2，Supplementary  Table 2 |
| Study selection | 9 | State the process for selecting studies (i.e., screening, eligibility, included in systematic review, and, if applicable, included in the meta-analysis). | 4**，**Table 1 |
| Data collection process | 10 | Describe method of data extraction from reports (e.g., piloted forms, independently, in duplicate) and any processes for obtaining and confirming data from investigators. | 2 |
| Data items | 11 | List and define all variables for which data were sought (e.g., PICOS, funding sources) and any assumptions and simplifications made. | 3 |
| **Geometry of the network** | **S1** | Describe methods used to explore the geometry of the treatment network under study and potential biases related to it. This should include how the evidence base has been graphically summarized for presentation, and what characteristics were compiled and used to describe the evidence base to readers. | **3** |
| Risk of bias within individual studies | 12 | Describe methods used for assessing risk of bias of individual studies (including specification of whether this was done at the study or outcome level), and how this information is to be used in any data synthesis. | 3 |
| Summary measures | 13 | State the principal summary measures (e.g., risk ratio, difference in means). *Also describe the use of additional summary measures assessed, such as treatment rankings and surface under the cumulative ranking curve (SUCRA) values, as well as modified approaches used to present summary findings from meta-analyses.* | 3 |
| Planned methods of analysis | 14 | Describe the methods of handling data and combining results of studies for each network meta-analysis. This should include, but not be limited to:   - *Handling of multi-arm trials;* - *Selection of variance structure;* - *Selection of prior distributions in Bayesian analyses; and* - *Assessment of model fit.* | 3 |
| **Assessment of Inconsistency** | **S2** | Describe the statistical methods used to evaluate the agreement of direct and indirect evidence in the treatment network(s) studied. Describe efforts taken to address its presence when found. | 3 |
| Risk of bias across studies | 15 | Specify any assessment of risk of bias that may affect the cumulative evidence (e.g., publication bias, selective reporting within studies). | 3 |
| Additional analyses | 16 | Describe methods of additional analyses if done, indicating which were pre-specified. This may include, but not be limited to, the following:   - Sensitivity or subgroup analyses; - Meta-regression analyses; - *Alternative formulations of the treatment network; and* - *Use of alternative prior distributions for Bayesian analyses (if applicable).* | 3 |
| **RESULTS†** |  |  |  |
| Study selection | 17 | Give numbers of studies screened, assessed for eligibility, and included in the review, with reasons for exclusions at each stage, ideally with a flow diagram. | 4，Figure 1 |
| **Presentation of network structure** | **S3** | Provide a network graph of the included studies to enable visualization of the geometry of the treatment network. | 9，Figure 5 |
| **Summary of network geometry** | **S4** | Provide a brief overview of characteristics of the treatment network. This may include commentary on the abundance of trials and randomized patients for the different interventions and pairwise comparisons in the network, gaps of evidence in the treatment network, and potential biases reflected by the network structure. | 7 |
| Study characteristics | 18 | For each study, present characteristics for which data were extracted (e.g., study size, PICOS, follow-up period) and provide the citations. | 3, Table 1 |
| Risk of bias within studies | 19 | Present data on risk of bias of each study and, if available, any outcome level assessment. | 4, Figure 2 |
| Results of individual studies | 20 | For all outcomes considered (benefits or harms), present, for each study: 1) simple summary data for each intervention group, and 2) effect estimates and confidence intervals. *Modified approaches may be needed to deal with information from larger networks.* | 7 |
| Synthesis of results | 21 | Present results of each meta-analysis done, including confidence/credible intervals. *In larger networks, authors may focus on comparisons versus a particular comparator (e.g. placebo or standard care), with full findings presented in an appendix. League tables and forest plots may be considered to summarize pairwise comparisons.* If additional summary measures were explored (such as treatment rankings), these should also be presented. | 7-8, Figure 3,4,6,7 |
| **Exploration for inconsistency** | **S5** | Describe results from investigations of inconsistency. This may include such information as measures of model fit to compare consistency and inconsistency models, *P* values from statistical tests, or summary of inconsistency estimates from different parts of the treatment network. | 8 , Table 2 |
| Risk of bias across studies | 22 | Present results of any assessment of risk of bias across studies for the evidence base being studied. | 10, Supplementary  Table 3-6 |
| Results of additional analyses | 23 | Give results of additional analyses, if done (e.g., sensitivity or subgroup analyses, meta-regression analyses, alternative network geometries studied, alternative choice of prior distributions for Bayesian analyses, and so forth). | 8 |
|  |  |  |  |
| **DISCUSSION** |  |  |  |
| Summary of evidence | 24 | Summarize the main findings, including the strength of evidence for each main outcome; consider their relevance to key groups (e.g., healthcare providers, users, and policy-makers). | 4-10 |
| Limitations | 25 | Discuss limitations at study and outcome level (e.g., risk of bias), and at review level (e.g., incomplete retrieval of identified research, reporting bias). *Comment on the validity of the assumptions, such as transitivity and consistency. Comment on any concerns regarding network geometry (e.g., avoidance of certain comparisons).* | 13 |
| Conclusions | 26 | Provide a general interpretation of the results in the context of other evidence, and implications for future research. | 11-12 |
|  |  |  |  |
| **FUNDING** |  |  |  |
| Funding | 27 | Describe sources of funding for the systematic review and other support (e.g., supply of data); role of funders for the systematic review. This should also include information regarding whether funding has been received from manufacturers of treatments in the network and/or whether some of the authors are content experts with professional conflicts of interest that could affect use of treatments in the network. | NA |

PICOS = population, intervention, comparators, outcomes, study design.

* Text in italics indicate S wording specific to reporting of network meta-analyses that has been added to guidance from the PRISMA statement.

† Authors may wish to plan for use of appendices to present all relevant information in full detail for items in this section.

| **Supplementary Table 2. Literature Search Strategy** | |
| --- | --- |
| **Pubmed** | (((("Carcinoma, Non-Small-Cell Lung"[Mesh]) OR (((((((((((Carcinoma, Non Small Cell Lung[Title/Abstract]) OR (Carcinomas, Non-Small-Cell Lung[Title/Abstract])) OR (Lung Carcinoma, Non-Small-Cell[Title/Abstract])) OR (Lung Carcinomas, Non-Small-Cell[Title/Abstract])) OR (Non-Small-Cell Lung Carcinomas[Title/Abstract])) OR (Carcinoma, Non-Small Cell Lung[Title/Abstract])) OR (Non-Small Cell Lung Cancer[Title/Abstract])) OR (Non-Small-Cell Lung Carcinoma[Title/Abstract])) OR (Non Small Cell Lung Carcinoma[Title/Abstract])) OR (Nonsmall Cell Lung Cancer[Title/Abstract])) OR (Non-Small Cell Lung Carcinoma[Title/Abstract]))) AND (("Brain Neoplasms"[Mesh]) OR (((((((((((((((((((((((((((((((((((((((((((((((((((((Brain Neoplasm) OR (Neoplasm, Brain)) OR (Brain Tumors)) OR (Brain Tumor)) OR (Tumor, Brain)) OR (Neoplasms, Brain)) OR (Brain Cancer)) OR (Brain Cancers)) OR (Cancer, Brain)) OR (Neoplasms, Brain, Malignant)) OR (Brain Neoplasms, Malignant)) OR (Brain Neoplasm, Malignant)) OR (Malignant Brain Neoplasm)) OR (Malignant Brain Neoplasms)) OR (Cancer of Brain)) OR (Cancer of the Brain)) OR (Malignant Neoplasms, Brain)) OR (Brain Malignant Neoplasm)) OR (Brain Malignant Neoplasms)) OR (Malignant Neoplasm, Brain)) OR (Malignant Primary Brain Tumors)) OR (Brain Neoplasms, Malignant, Primary)) OR (Brain Neoplasms, Primary Malignant)) OR (Malignant Primary Brain Neoplasms)) OR (Primary Malignant Brain Neoplasms)) OR (Primary Malignant Brain Tumors)) OR (Neoplasms, Intracranial)) OR (Intracranial Neoplasm)) OR (Neoplasm, Intracranial)) OR (Intracranial Neoplasms)) OR (Benign Neoplasms, Brain)) OR (Benign Neoplasm, Brain)) OR (Brain Benign Neoplasm)) OR (Brain Benign Neoplasms)) OR (Brain Neoplasms, Benign)) OR (Benign Brain Neoplasm)) OR (Benign Brain Neoplasms)) OR (Brain Neoplasm, Benign)) OR (Neoplasms, Brain, Benign)) OR (Brain Tumor, Primary)) OR (Primary Brain Tumor)) OR (Primary Brain Tumors)) OR (Primary Brain Neoplasms)) OR (Brain Neoplasms, Primary)) OR (Primary Brain Neoplasm)) OR (Brain Neoplasm, Primary)) OR (Neoplasms, Brain, Primary)) OR (Brain Tumor, Recurrent)) OR (Brain Tumors, Recurrent)) OR (Recurrent Brain Tumor)) OR (Recurrent Brain Tumors)) OR (Brain Metastases)) OR (Brain Metastase)))) AND (("Immune Checkpoint Inhibitors"[Mesh]) OR (((((((((((((((((((((((((((((((((Checkpoint Inhibitors, Immune[Title/Abstract]) OR (Immune Checkpoint Blockers[Title/Abstract])) OR (Checkpoint Blockers, Immune[Title/Abstract])) OR (Immune Checkpoint Inhibitor[Title/Abstract])) OR (Checkpoint Inhibitor, Immune[Title/Abstract])) OR (CTLA-4 Inhibitors[Title/Abstract])) OR (CTLA 4 Inhibitors[Title/Abstract])) OR (Cytotoxic T-Lymphocyte-Associated Protein 4 Inhibitors[Title/Abstract])) OR (Cytotoxic T Lymphocyte Associated Protein 4 Inhibitors[Title/Abstract])) OR (Cytotoxic T-Lymphocyte-Associated Protein 4 Inhibitor[Title/Abstract])) OR (Cytotoxic T Lymphocyte Associated Protein 4 Inhibitor[Title/Abstract])) OR (CTLA-4 Inhibitor[Title/Abstract])) OR (CTLA 4 Inhibitor[Title/Abstract])) OR (PD-1 Inhibitors[Title/Abstract])) OR (PD 1 Inhibitors[Title/Abstract])) OR (Programmed Cell Death Protein 1 Inhibitor[Title/Abstract])) OR (Programmed Cell Death Protein 1 Inhibitors[Title/Abstract])) OR (PD-1 Inhibitor[Title/Abstract])) OR (Inhibitor, PD-1[Title/Abstract])) OR (PD 1 Inhibitor[Title/Abstract])) OR (Immune Checkpoint Blockade[Title/Abstract])) OR (Checkpoint Blockade, Immune[Title/Abstract])) OR (Immune Checkpoint Inhibition[Title/Abstract])) OR (Checkpoint Inhibition, Immune[Title/Abstract])) OR (PD-L1 Inhibitors[Title/Abstract])) OR (PD L1 Inhibitors[Title/Abstract])) OR (Programmed Death-Ligand 1 Inhibitors[Title/Abstract])) OR (Programmed Death Ligand 1 Inhibitors[Title/Abstract])) OR (PD-L1 Inhibitor[Title/Abstract])) OR (PD L1 Inhibitor[Title/Abstract])) OR (PD-1-PD-L1 Blockade[Title/Abstract])) OR (Blockade, PD-1-PD-L1[Title/Abstract])) OR (PD 1 PD L1 Blockade[Title/Abstract])))) AND ((((((((randomized controlled trial[Publication Type]) OR (controlled clinical trial[Publication Type])) OR (randomized[Title/Abstract])) OR (placebo[Title/Abstract])) OR (drug therapy[Title/Abstract])) OR (randomly[Title/Abstract])) OR (trial[Title/Abstract])) OR (groups[Title/Abstract]))  99 |
| **Cochrane** | #1 MeSH descriptor: [Carcinoma, Non-Small-Cell Lung] explode all trees 6910  #2 ("carcinoma, non small cell lung" OR "Carcinomas, Non-Small-Cell Lung" OR "Lung Carcinoma, Non-Small-Cell" OR "Lung Carcinomas, Non-Small-Cell" OR "Non-Small-Cell Lung Carcinomas" OR "Carcinoma, Non-Small Cell Lung" OR "Non-Small Cell Lung Cancer" OR "Non-Small-Cell Lung Carcinoma" OR "Non Small Cell Lung Carcinoma" OR "Nonsmall Cell Lung Cancer" OR "Non-Small Cell Lung Carcinoma"):ti,ab,kw 17919  #3 #1 OR #2 17919  #4 MeSH descriptor: [Brain Neoplasms] explode all trees 3255  #5 ("Brain Neoplasm" OR "Neoplasm, Brain" OR "Brain Tumors" OR "Brain Tumor" OR "Tumor, Brain" OR "Neoplasms, Brain" OR "Brain Cancer" OR "Brain Cancers" OR "Cancer, Brain" OR "Neoplasms, Brain, Malignant" OR "Brain Neoplasms, Malignant" OR "Brain Neoplasm, Malignant" OR "Malignant Brain Neoplasm" OR "Malignant Brain Neoplasms" OR "Cancer of Brain" OR "Cancer of the Brain" OR "Malignant Neoplasms, Brain" OR "Brain Malignant Neoplasm" OR "Brain Malignant Neoplasms" OR "Malignant Neoplasm, Brain" OR "Malignant Primary Brain Tumors" OR "Brain Neoplasms, Malignant, Primary" OR "Brain Neoplasms, Primary Malignant" OR "Malignant Primary Brain Neoplasms" OR "Primary Malignant Brain Neoplasms" OR "Primary Malignant Brain Tumors" OR "Neoplasms, Intracranial" OR "Intracranial Neoplasm" OR "Neoplasm, Intracranial" OR "Intracranial Neoplasms" OR "Benign Neoplasms, Brain" OR "Benign Neoplasm, Brain" OR "Brain Benign Neoplasm" OR "Brain Benign Neoplasms" OR "Brain Neoplasms, Benign" OR "Benign Brain Neoplasm" OR "Benign Brain Neoplasms" OR "Brain Neoplasm, Benign" OR "Neoplasms, Brain, Benign" OR "Brain Tumor, Primary" OR "Primary Brain Tumor" OR "Primary Brain Tumors" OR "Primary Brain Neoplasms" OR "Brain Neoplasms, Primary" OR "Primary Brain Neoplasm" OR "Brain Neoplasm, Primary" OR "Neoplasms, Brain, Primary" OR "Brain Tumor, Recurrent" OR "Brain Tumors, Recurrent" OR "Recurrent Brain Tumor" OR "Recurrent Brain Tumors" OR "Brain Metastases" OR "Brain Metastase") 5224  #6 #4 OR #5 7014  #7 MeSH descriptor: [Immune Checkpoint Inhibitors] explode all trees 414  #8 ("Checkpoint Inhibitors, Immune" OR "Immune Checkpoint Blockers" OR "Checkpoint Blockers, Immune" OR "Immune Checkpoint Inhibitor" OR "Checkpoint Inhibitor, Immune" OR "CTLA-4 Inhibitors" OR "CTLA 4 Inhibitors" OR "Cytotoxic T-Lymphocyte-Associated Protein 4 Inhibitors" OR "Cytotoxic T Lymphocyte Associated Protein 4 Inhibitors" OR "Cytotoxic T-Lymphocyte-Associated Protein 4 Inhibitor" OR "Cytotoxic T Lymphocyte Associated Protein 4 Inhibitor" OR "CTLA-4 Inhibitor" OR "CTLA 4 Inhibitor" OR "PD-1 Inhibitors" OR "PD 1 Inhibitors" OR "Programmed Cell Death Protein 1 Inhibitor" OR "Programmed Cell Death Protein 1 Inhibitors" OR "PD-1 Inhibitor" OR "Inhibitor, PD-1" OR "PD 1 Inhibitor" OR "Immune Checkpoint Blockade" OR "Checkpoint Blockade, Immune" OR "Immune Checkpoint Inhibition" OR "Checkpoint Inhibition, Immune" OR "PD-L1 Inhibitors" OR "PD L1 Inhibitors" OR "Programmed Death-Ligand 1 Inhibitors" OR "Programmed Death Ligand 1 Inhibitors" OR "PD-L1 Inhibitor" OR "PD L1 Inhibitor" OR "PD-1-PD-L1 Blockade" OR "Blockade, PD-1-PD-L1" OR "PD 1 PD L1 Blockade"):ti,ab,kw 2410  #9 #7 OR #8 2640  #10 #3 AND #6 AND #9 41 |
| **Embase** | #11. #3 AND #6 AND #9 AND #10 134  #10. 'randomized controlled trial'/exp OR 'randomized controlled trial' 1,439,402  #9. #7 OR #8 81,526  #8. 'checkpoint inhibitors, immune':ab,ti OR 'immune checkpoint blockers':ab,ti OR 'checkpoint blockers, immune':ab,ti OR 'immune checkpoint inhibitor':ab,ti OR 'checkpoint inhibitor, immune':ab,ti OR 'ctla-4 inhibitors':ab,ti OR 'ctla 4 inhibitors':ab,ti OR 'cytotoxic t-lymphocyte-associated protein 4 inhibitors':ab,ti OR 'cytotoxic t lymphocyte associated protein 4 inhibitors':ab,ti OR 'cytotoxic t-lymphocyte-associated protein 4 inhibitor':ab,ti OR 'cytotoxic t lymphocyte associated protein 4 inhibitor':ab,ti OR 'ctla-4 inhibitor':ab,ti OR 'ctla 4 inhibitor':ab,ti OR 'pd-1 inhibitors':ab,ti OR 'pd 1 inhibitors':ab,ti OR 'programmed cell death protein 1 inhibitor':ab,ti OR 'programmed cell death protein 1 inhibitors':ab,ti OR 'pd-1 inhibitor':ab,ti OR 'inhibitor, pd-1':ab,ti OR 'pd 1 inhibitor':ab,ti OR 'immune checkpoint blockade':ab,ti OR 'checkpoint blockade, immune':ab,ti OR 'immune checkpoint inhibition':ab,ti OR 'checkpoint inhibition, immune':ab,ti OR 'pd-l1 inhibitors':ab,ti OR 'pd l1 inhibitors':ab,ti OR 'programmed death-ligand 1 inhibitors':ab,ti OR 'programmed death ligand 1 inhibitors':ab,ti OR 'pd-l1 inhibitor':ab,ti OR 'pd l1 inhibitor':ab,ti OR 'pd-1-pd-l1 blockade':ab,ti OR 'blockade, pd-1-pd-l1':ab,ti OR 'pd 1 pd l1 blockade':ab,ti 46195  #7. 'immune checkpoint inhibitor'/exp OR 'immune checkpoint inhibitor' 57,965  #6. #4 OR #5 315,064  #5. 'brain neoplasm':ab,ti OR 'neoplasm, brain':ab,ti OR 'brain tumors':ab,ti OR 'brain tumor':ab,ti OR 'tumor, brain':ab,ti OR 'neoplasms, brain':ab,ti OR 'brain cancer':ab,ti OR 'brain cancers':ab,ti OR 'cancer, brain':ab,ti OR 'neoplasms, brain, malignant':ab,ti OR 'brain neoplasms, malignant':ab,ti OR 'brain neoplasm, malignant':ab,ti OR 'malignant brain neoplasm':ab,ti OR 'malignant brain neoplasms':ab,ti OR 'cancer of brain':ab,ti OR 'cancer of the brain':ab,ti OR 'malignant neoplasms, brain':ab,ti OR 'brain malignant neoplasm':ab,ti OR 'brain malignant neoplasms':ab,ti OR 'malignant neoplasm, brain':ab,ti OR 'malignant primary brain tumors':ab,ti OR 'brain neoplasms, malignant, primary':ab,ti OR 'brain neoplasms, primary malignant':ab,ti OR 'malignant primary brain neoplasms':ab,ti OR 'primary malignant brain neoplasms':ab,ti OR 'primary malignant brain tumors':ab,ti OR 'neoplasms, intracranial':ab,ti OR 'intracranial neoplasm':ab,ti OR 'neoplasm, intracranial':ab,ti OR 'intracranial neoplasms':ab,ti OR 'benign neoplasms, brain':ab,ti OR 'benign neoplasm, brain':ab,ti OR 'brain benign neoplasm':ab,ti OR 'brain benign neoplasms':ab,ti OR 'brain neoplasms, benign':ab,ti OR 'benign brain neoplasm':ab,ti OR 'benign brain neoplasms':ab,ti OR 'brain neoplasm, benign':ab,ti OR 'neoplasms, brain, benign':ab,ti OR 'brain tumor, primary':ab,ti OR 'primary brain tumor':ab,ti OR 'primary brain tumors':ab,ti OR 'primary brain neoplasms':ab,ti OR 'brain neoplasms, primary':ab,ti OR 'primary brain neoplasm':ab,ti OR 'brain neoplasm, primary':ab,ti OR 'neoplasms, brain, primary':ab,ti OR 'brain tumor, recurrent':ab,ti OR 'brain tumors, recurrent':ab,ti OR 'recurrent brain tumor':ab,ti OR 'recurrent brain tumors':ab,ti OR 'brain metastases':ab,ti OR 'brain metastase':ab,ti 114171  #4. 'brain tumor'/exp OR 'brain tumor' 299,948  #3. #1 OR #2 276,909  #2. 'carcinoma, non small cell lung':ab,ti OR 'carcinomas, non-small-cell lung':ab,ti OR 'lung carcinoma, non-small-cell':ab,ti OR 'lung carcinomas, non-small-cell':ab,ti OR 'non-small-cell lung carcinomas':ab,ti OR 'carcinoma, non-small cell lung':ab,ti OR 'non-small cell lung cancer':ab,ti OR 'non-small-cell lung carcinoma':ab,ti OR 'non small cell lung carcinoma':ab,ti OR 'nonsmall cell lung cancer':ab,ti OR 'non-small cell lung carcinoma':ab,ti  160072  #1. 'non small cell lung cancer'/exp OR 'non small cell lung cancer' 274,453 |
| **Web of Science** | #1 ((((((((((((TS=(Carcinoma, Non-Small-Cell Lung)) OR TS=(Carcinoma, Non Small Cell Lung)) OR TS=(Carcinomas, Non-Small-Cell Lung)) OR TS=(Lung Carcinoma, Non-Small-Cell)) OR TS=(Lung Carcinomas, Non-Small-Cell))) OR TS=(Non-Small-Cell Lung Carcinomas)) OR TS=(Carcinoma, Non-Small Cell Lung)) OR TS=(Non-Small Cell Lung Cancer)) OR TS=(Non-Small-Cell Lung Carcinoma)) OR TS=(Non Small Cell Lung Carcinoma)) OR TS=(Nonsmall Cell Lung Cancer)) OR TS=(Non-Small Cell Lung Carcinoma) and Preprint Citation Index (Exclude – Database) and Research Commons (Exclude – Database) [194,679](https://www.webofscience.com/wos/alldb/summary/ddb1e217-292e-4d81-b1c6-6b02717c2eeb-018788e475/relevance/1)  #2 TS=(Brain Neoplasm) OR TS=(Neoplasm, Brain) OR TS=(Brain Tumors) OR TS=(Brain Tumor) OR TS=(Tumor, Brain) OR TS=(Neoplasms, Brain) OR TS=(Brain Cancer) OR TS=(Brain Cancers) OR TS=(Cancer, Brain) OR TS=(Neoplasms, Brain, Malignant) OR TS=(Brain Neoplasms, Malignant) OR TS=(Brain Neoplasm, Malignant) OR TS=(Malignant Brain Neoplasm) OR TS=(Malignant Brain Neoplasms) OR TS=(Cancer of Brain) OR TS=(Cancer of the Brain) OR TS=(Malignant Neoplasms, Brain) OR TS=(Brain Malignant Neoplasm) OR TS=(Brain Malignant Neoplasms) OR TS=(Malignant Neoplasm, Brain) OR TS=(Malignant Primary Brain Tumors) OR TS=(Brain Neoplasms, Malignant, Primary) OR TS=(Brain Neoplasms, Primary Malignant) OR TS=(Malignant Primary Brain Neoplasms) OR TS=(Primary Malignant Brain Neoplasms) OR TS=(Primary Malignant Brain Tumors) OR TS=(Neoplasms, Intracranial) OR TS=(Intracranial Neoplasm) OR TS=(Neoplasm, Intracranial) OR TS=(Intracranial Neoplasms) OR TS=(Benign Neoplasms, Brain) OR TS=(Benign Neoplasm, Brain) OR TS=(Brain Benign Neoplasm) OR TS=(Brain Benign Neoplasms) OR TS=(Brain Neoplasms, Benign) OR TS=(Benign Brain Neoplasm) OR TS=(Benign Brain Neoplasms) OR TS=(Brain Neoplasm, Benign) OR TS=(Neoplasms, Brain, Benign) OR TS=(Brain Tumor, Primary) OR TS=(Primary Brain Tumor) OR TS=(Primary Brain Tumors) OR TS=(Primary Brain Neoplasms) OR TS=(Brain Neoplasms, Primary) OR TS=(Primary Brain Neoplasm) OR TS=(Brain Neoplasm, Primary) OR TS=(Neoplasms, Brain, Primary) OR TS=(Brain Tumor, Recurrent) OR TS=(Brain Tumors, Recurrent) OR TS=(Recurrent Brain Tumor) OR TS=(Recurrent Brain Tumors) OR TS=(Brain Metastases) OR TS=(Brain Metastase) and Preprint Citation Index (Exclude – Database) and Research Commons (Exclude – Database) [495,228](https://www.webofscience.com/wos/alldb/summary/53f670b5-8d9e-48f1-934b-db5bf96b4145-018788f0eb/relevance/1)  #3 TS=(Checkpoint Inhibitors, Immune) OR TS=(Immune Checkpoint Blockers) OR TS=(Checkpoint Blockers, Immune) OR TS=(Immune Checkpoint Inhibitor) OR TS=(Checkpoint Inhibitor, Immune) OR TS=(CTLA-4 Inhibitors) OR TS=(CTLA 4 Inhibitors) OR TS=(Cytotoxic T-Lymphocyte-Associated Protein 4 Inhibitors) OR TS=(Cytotoxic T Lymphocyte Associated Protein 4 Inhibitors) OR TS=(Cytotoxic T-Lymphocyte-Associated Protein 4 Inhibitor) OR TS=(Cytotoxic T Lymphocyte Associated Protein 4 Inhibitor) OR TS=(CTLA-4 Inhibitor) OR TS=(CTLA 4 Inhibitor) OR TS=(PD-1 Inhibitors) OR TS=(PD 1 Inhibitors) OR TS=(Programmed Cell Death Protein 1 Inhibitor) OR TS=(Programmed Cell Death Protein 1 Inhibitors) OR TS=(PD-1 Inhibitor) OR TS=(Inhibitor, PD-1) OR TS=(PD 1 Inhibitor) OR TS=(Immune Checkpoint Blockade) OR TS=(Checkpoint Blockade, Immune) OR TS=(Immune Checkpoint Inhibition) OR TS=(Checkpoint Inhibition, Immune) OR TS=(PD-L1 Inhibitors) OR TS=(PD L1 Inhibitors) OR TS=(Programmed Death-Ligand 1 Inhibitors) OR TS=(Programmed Death Ligand 1 Inhibitors) OR TS=(PD-L1 Inhibitor) OR TS=(PD L1 Inhibitor) OR TS=(PD-1-PD-L1 Blockade) OR TS=(Blockade, PD-1-PD-L1) OR TS=(PD 1 PD L1 Blockade) and Preprint Citation Index (Exclude – Database) and Research Commons (Exclude – Database) [137,686](https://www.webofscience.com/wos/alldb/summary/90cf4c71-6e78-4ec5-aafa-6fb719c36c40-018788fa8c/relevance/1)  #4 TS=(random* OR randomi* OR randomly OR placebo* OR sham OR trial OR "clinical trial" OR "controlled clinical trial" OR "double blind" OR "single blind" OR "triple blind" OR crossover OR "cross over" OR "parallel group" OR cluster random* OR RCT) and Preprint Citation Index (Exclude – Database) and Research Commons (Exclude – Database) [6,147,169](https://www.webofscience.com/wos/alldb/summary/ada309d3-a0e6-4d78-8ed0-ea2808ba9bde-018788fe93/relevance/1)  #5 #1 AND #2 AND #3 AND #4 and Preprint Citation Index (Exclude – Database) and Research Commons (Exclude – Database) 362 |


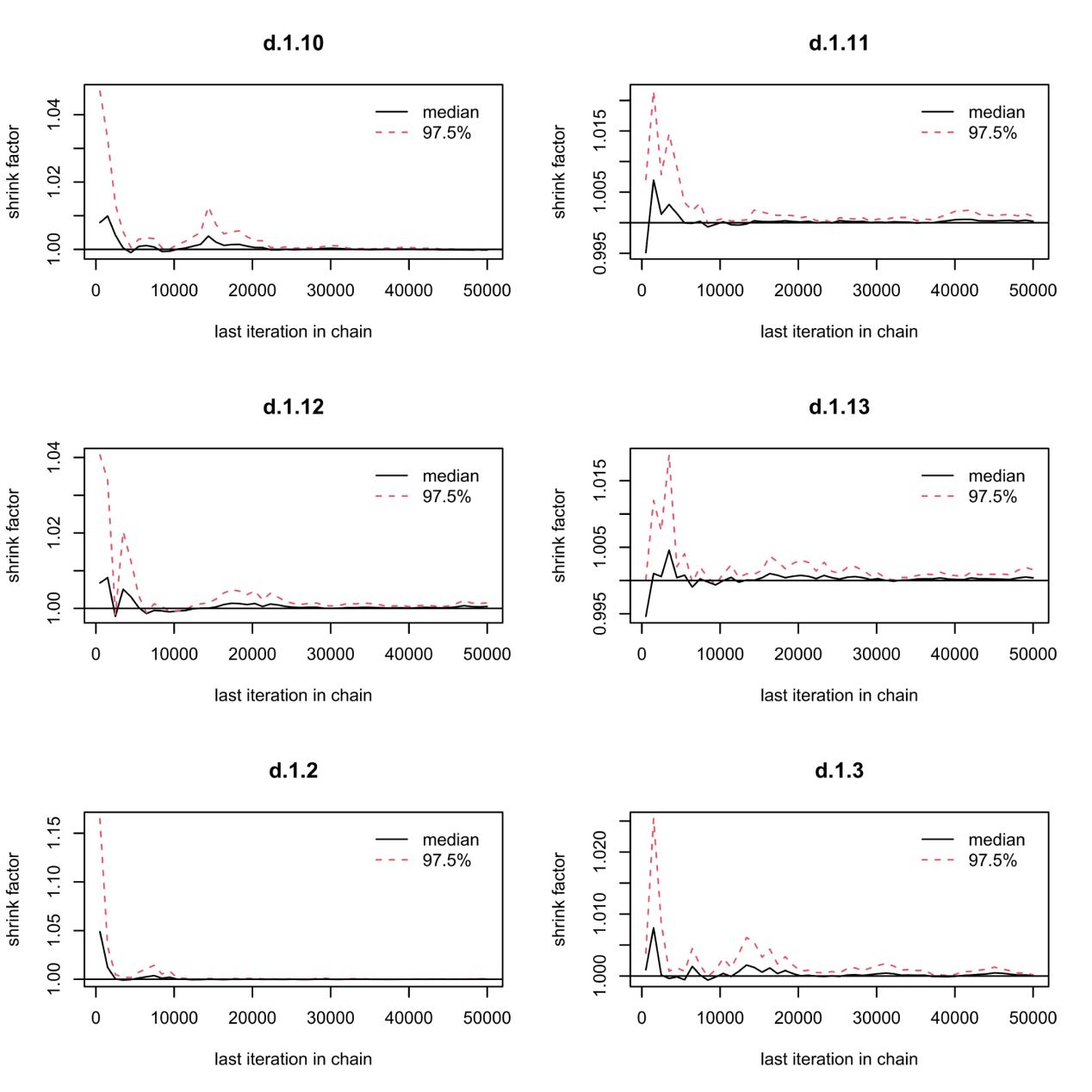


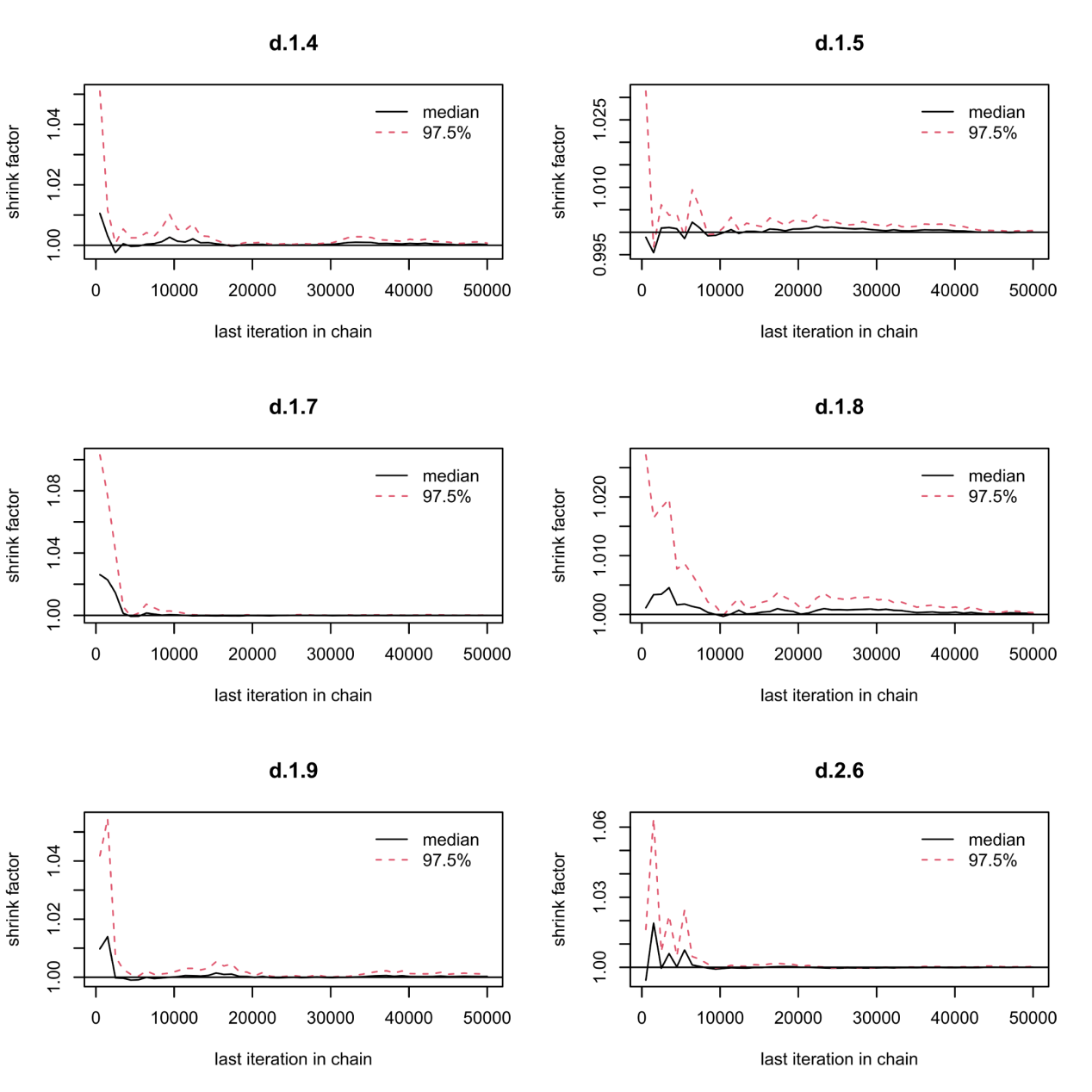


**Supplementary Figure 1.** Convergence diagnostics for the overall survival (OS) model in the brain metastasis subgroup.


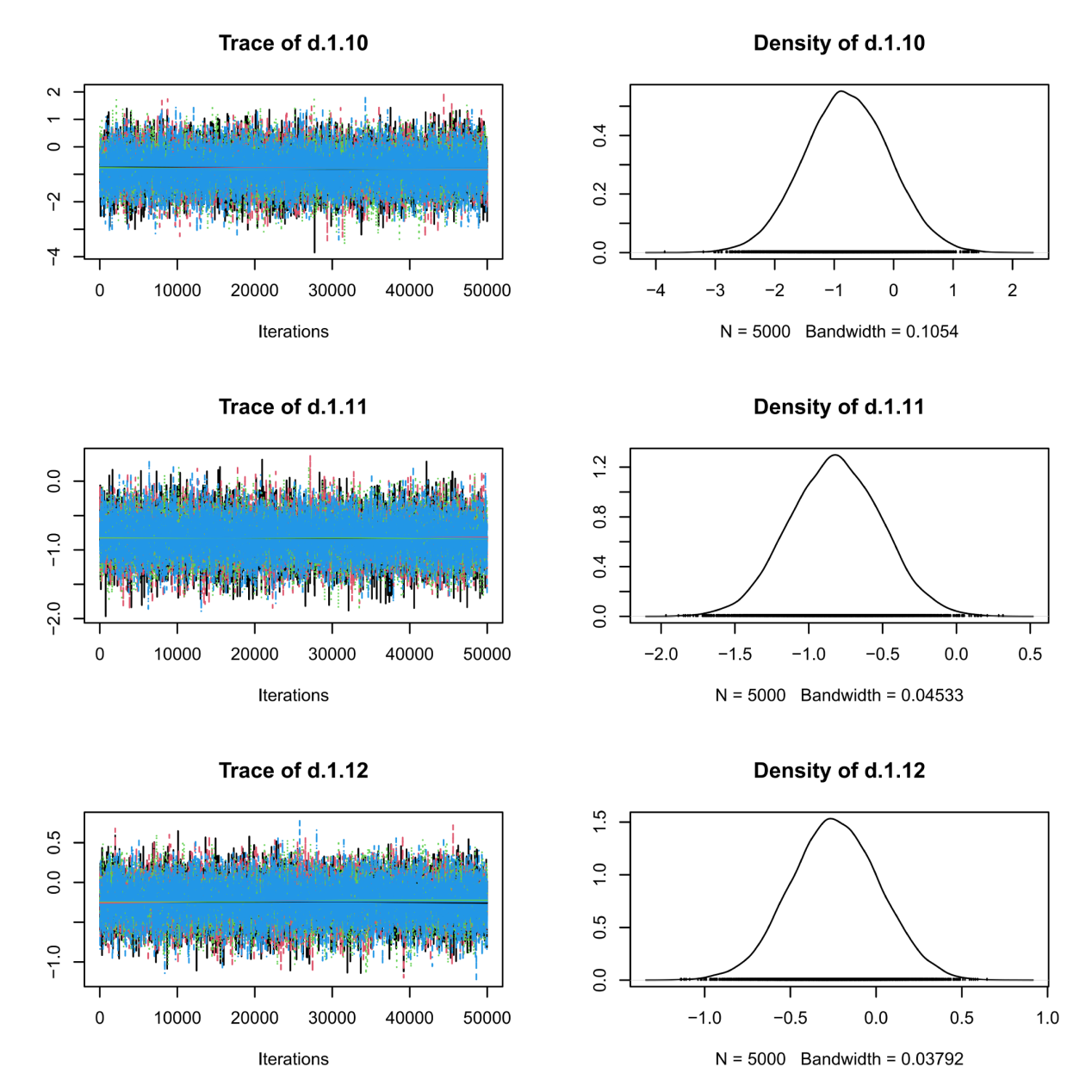


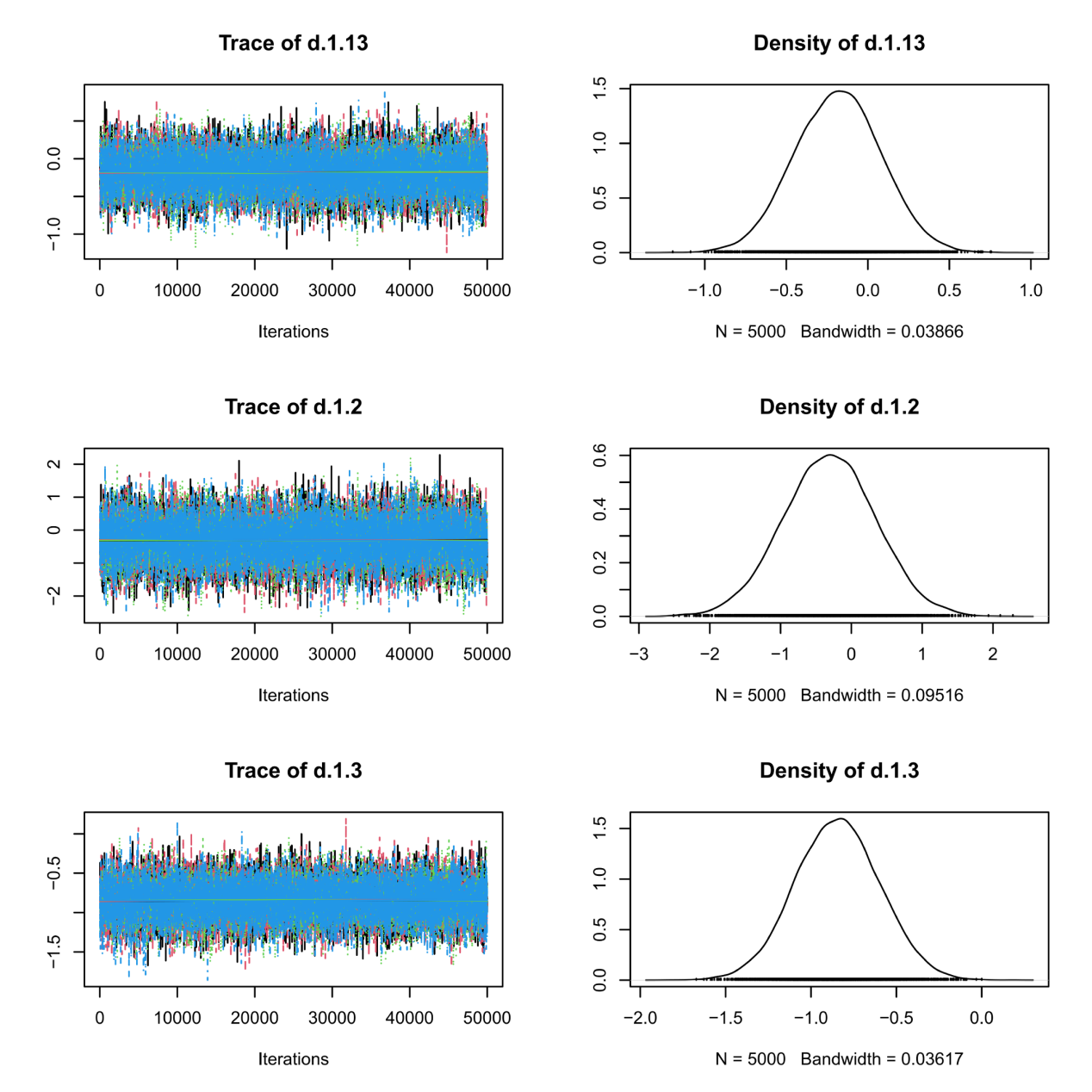


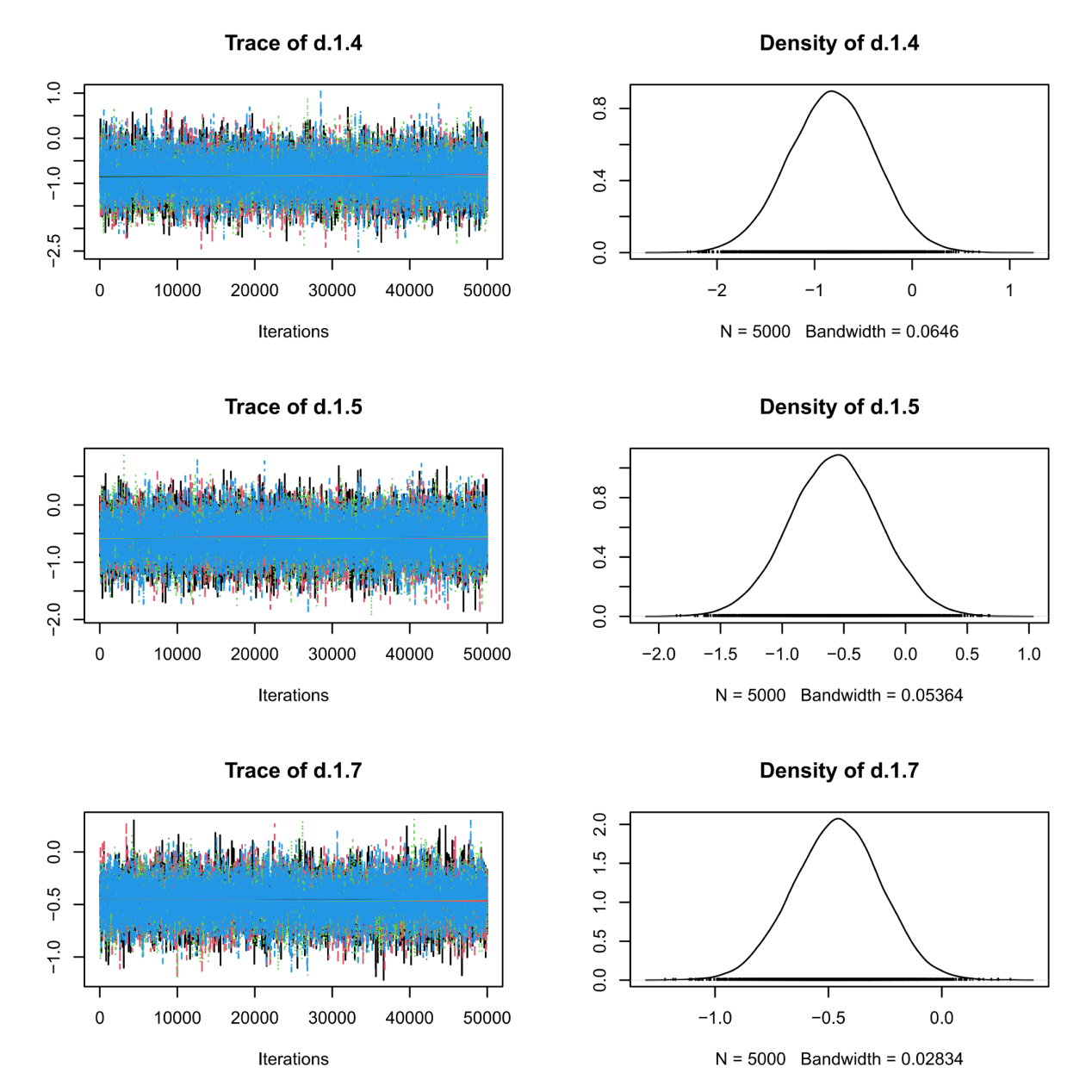


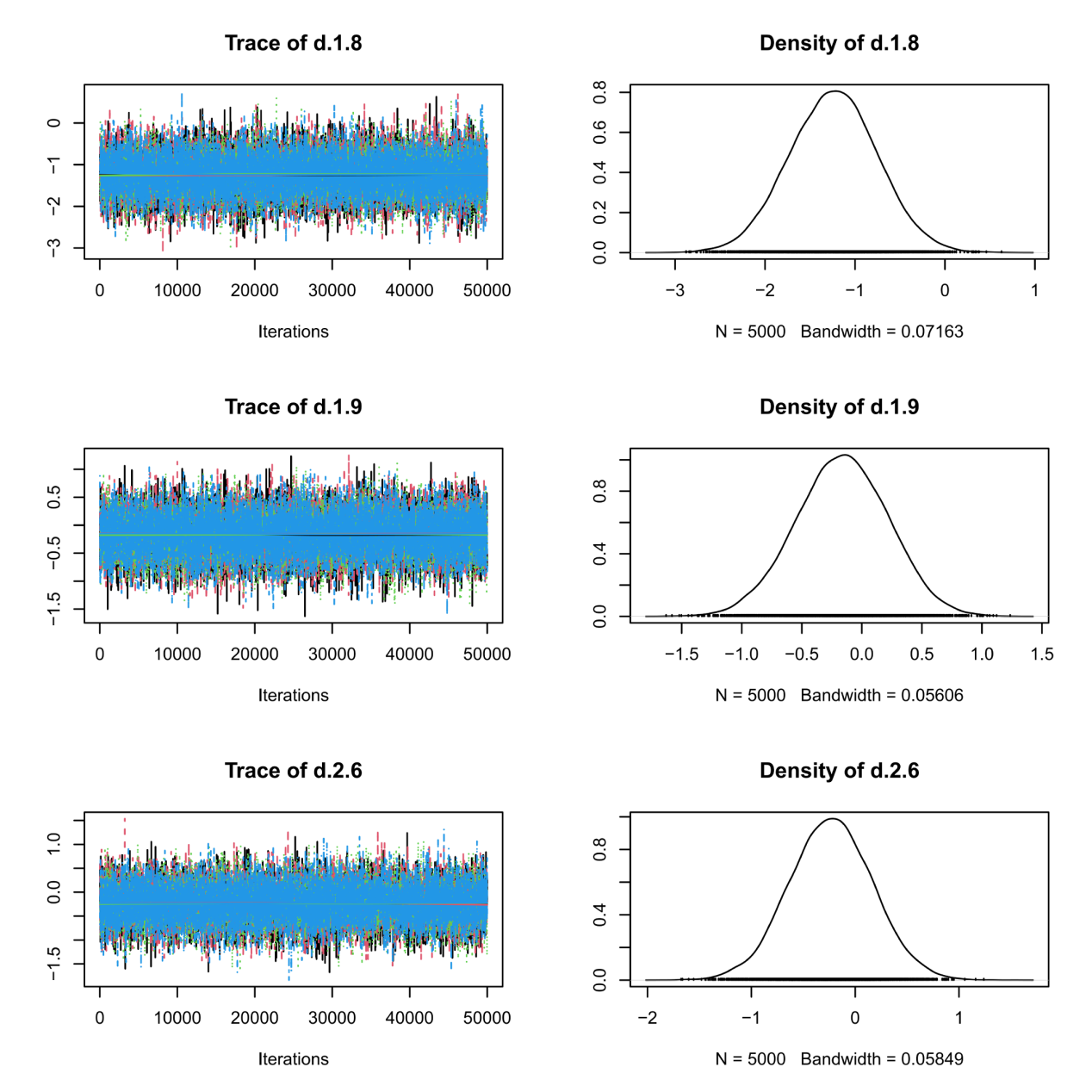


**Supplementary Figure 2.** Trace and density plots for the overall survival (OS) model in the brain metastasis subgroup.


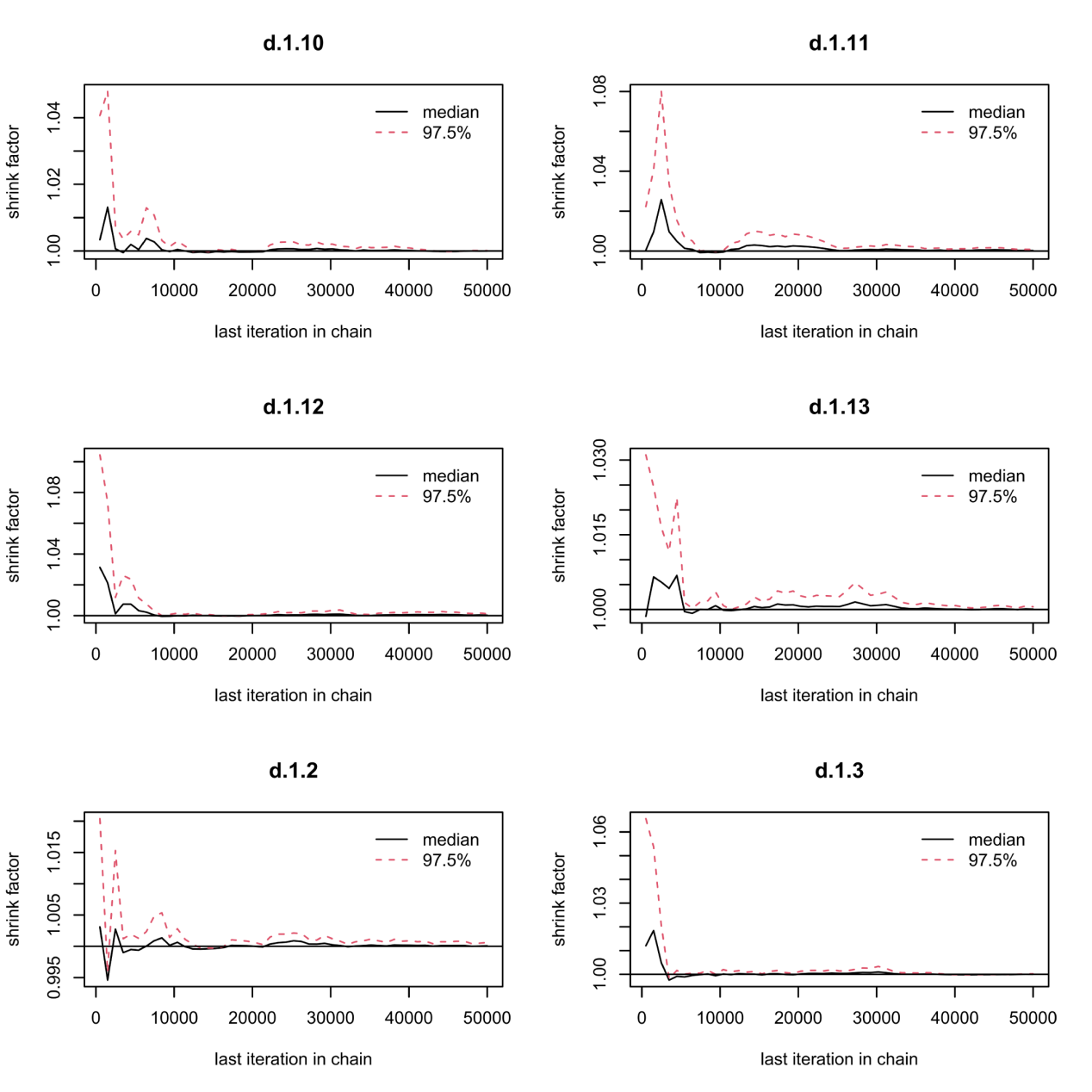


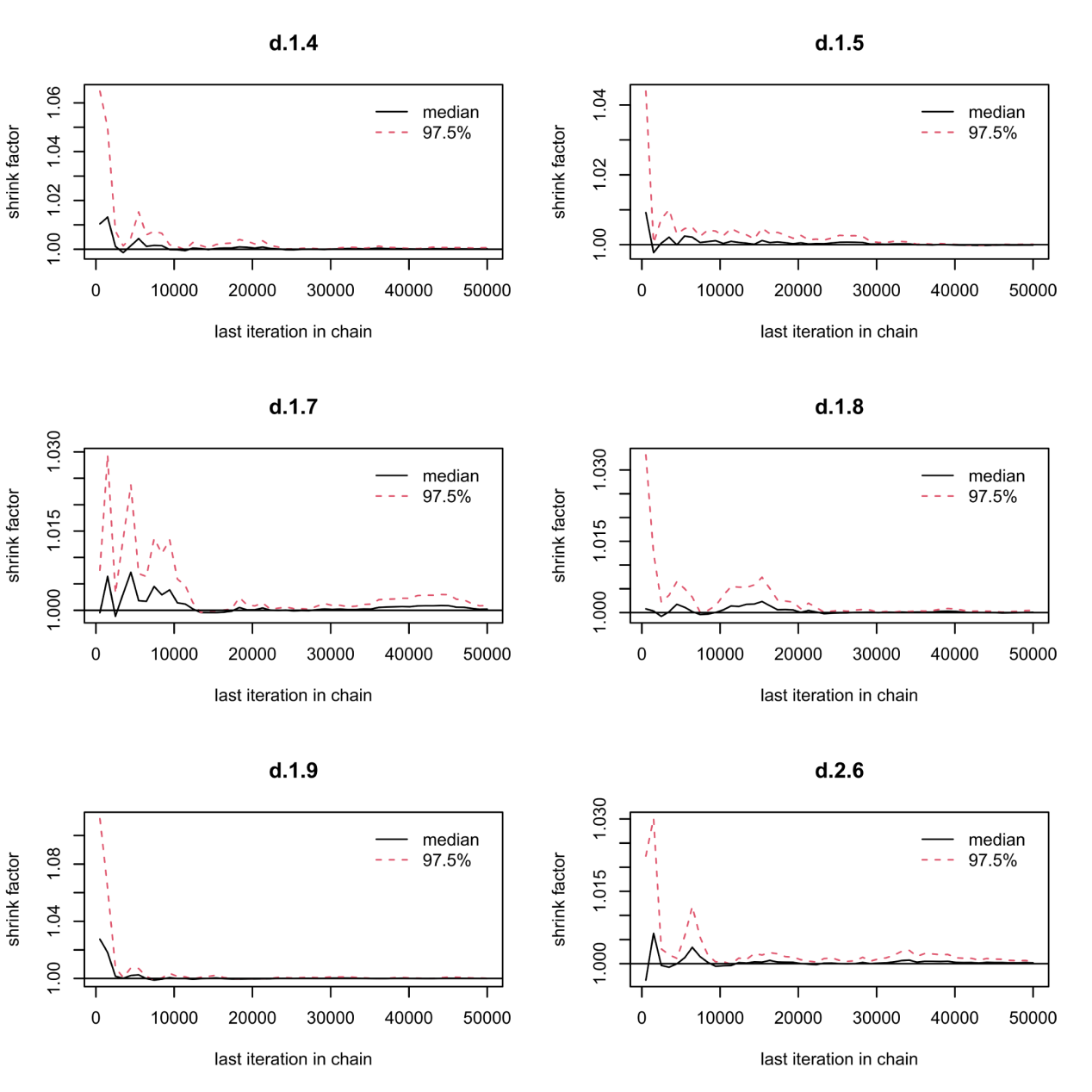


**Supplementary Figure 3.** Convergence diagnostics for the overall survival (OS) model in the non-brain metastasis subgroup.


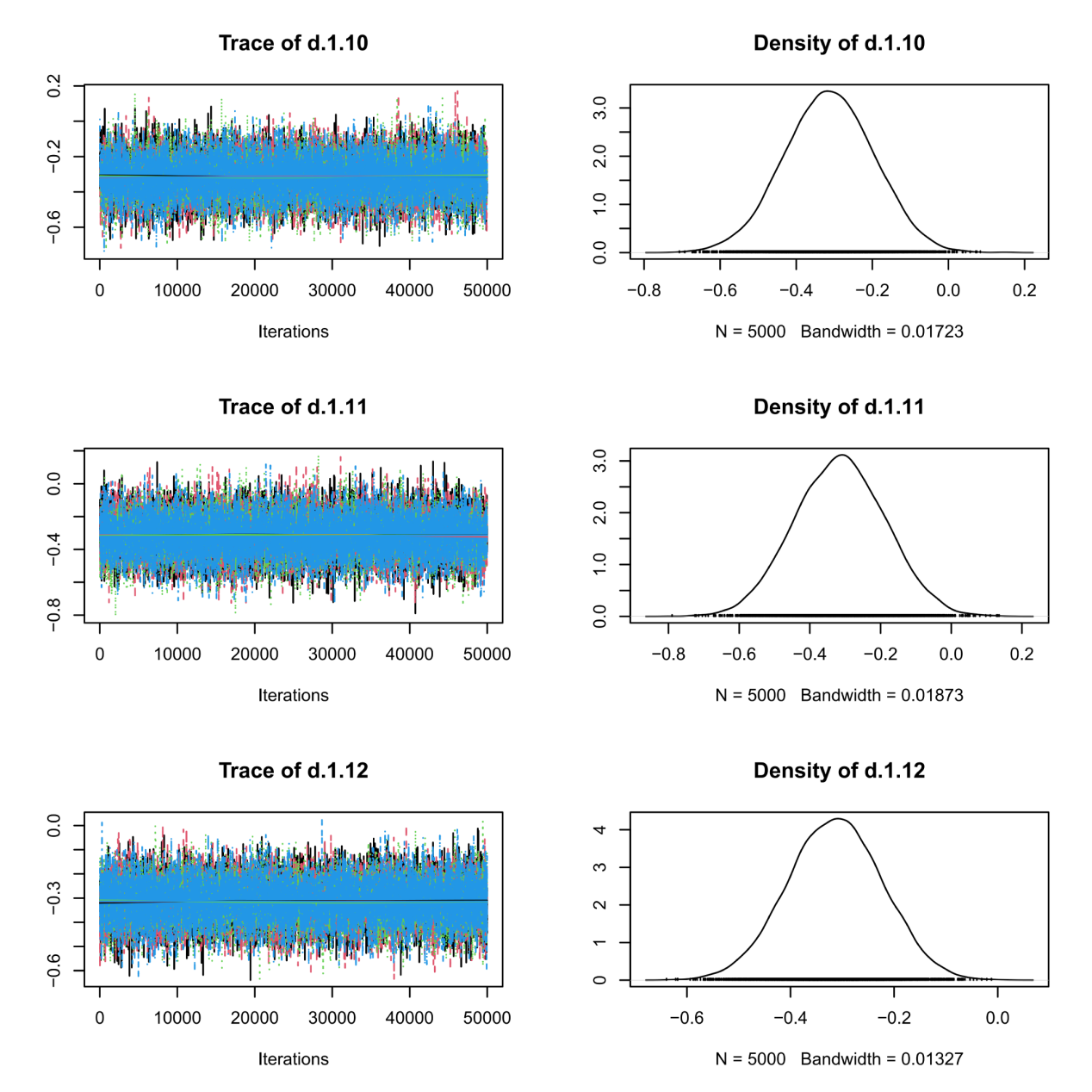


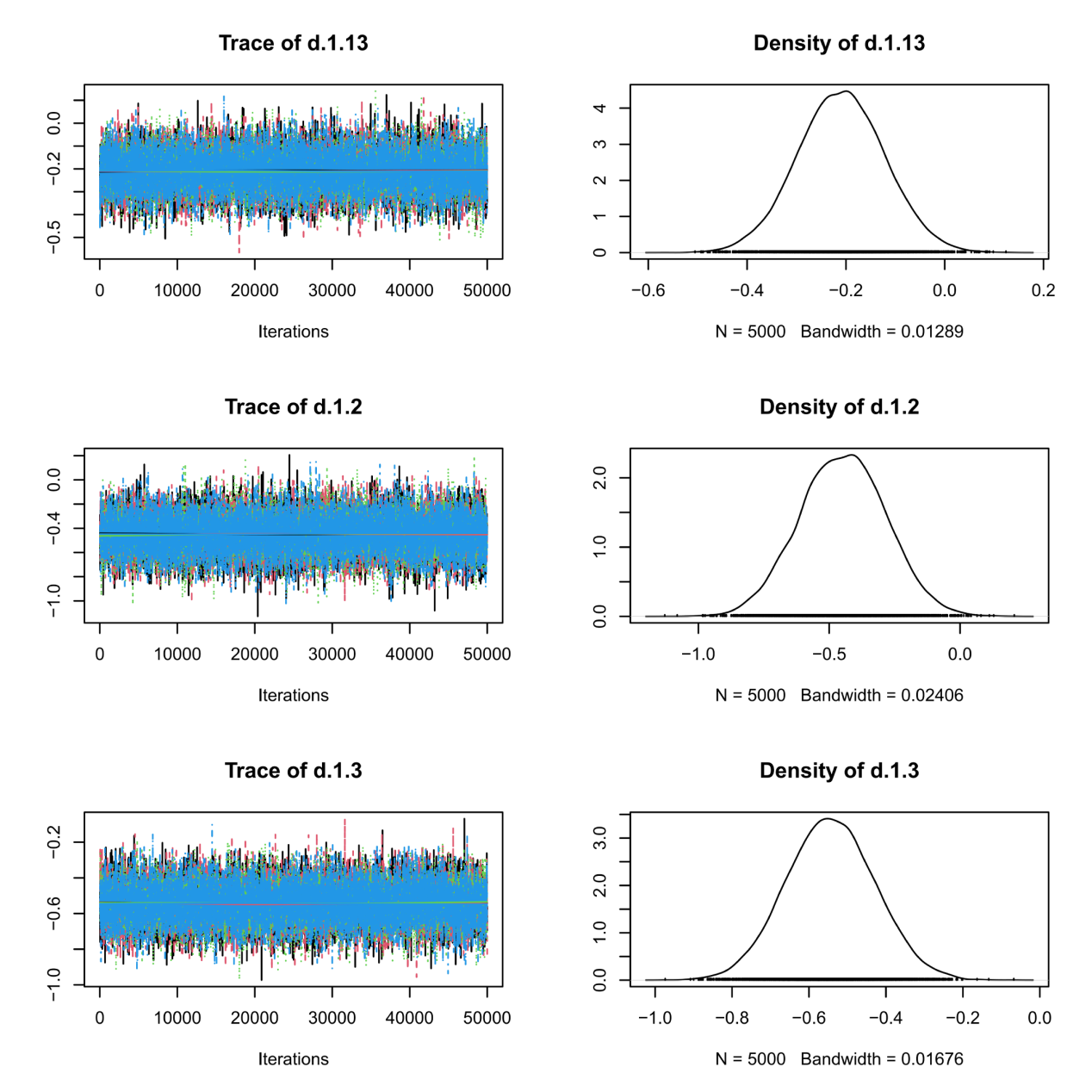


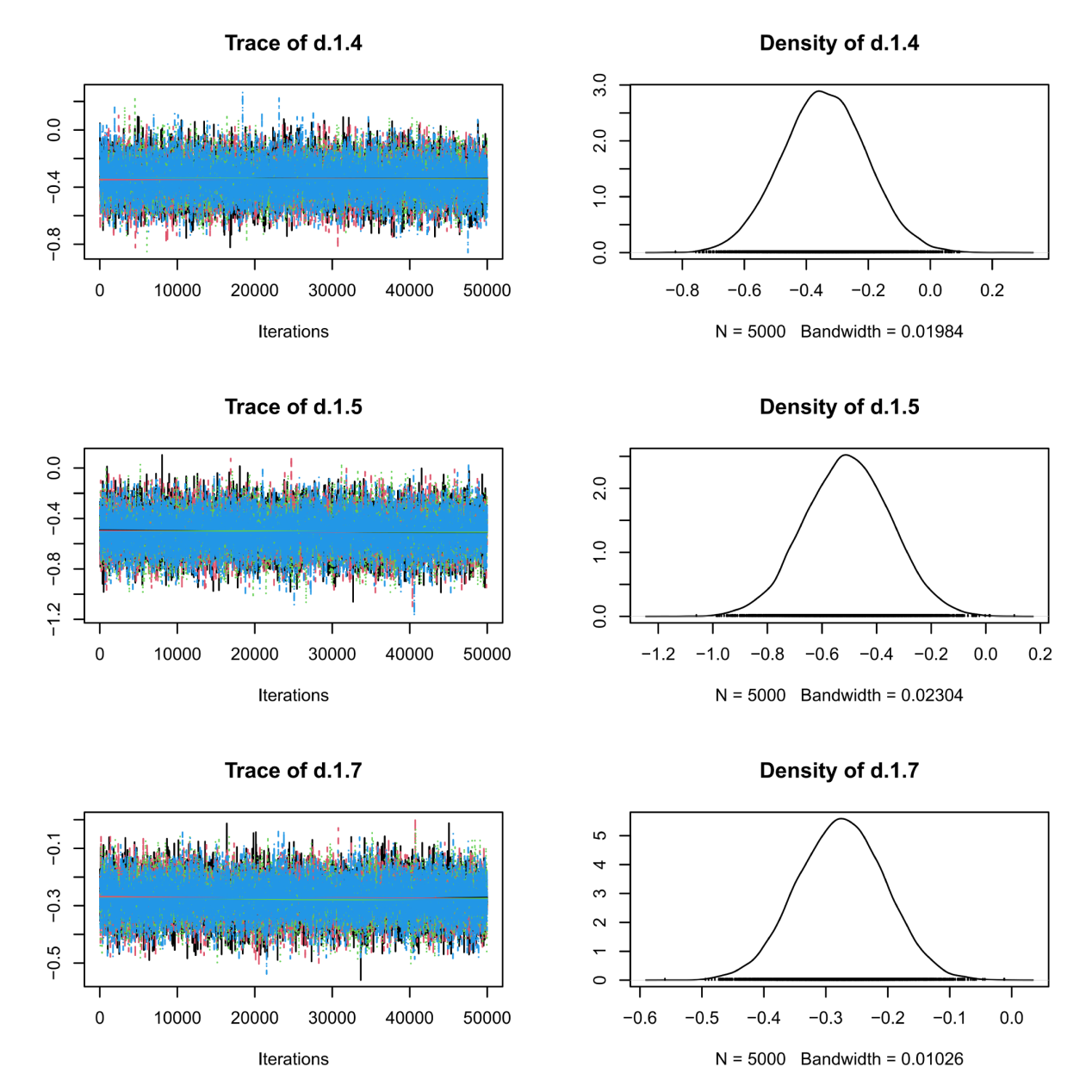


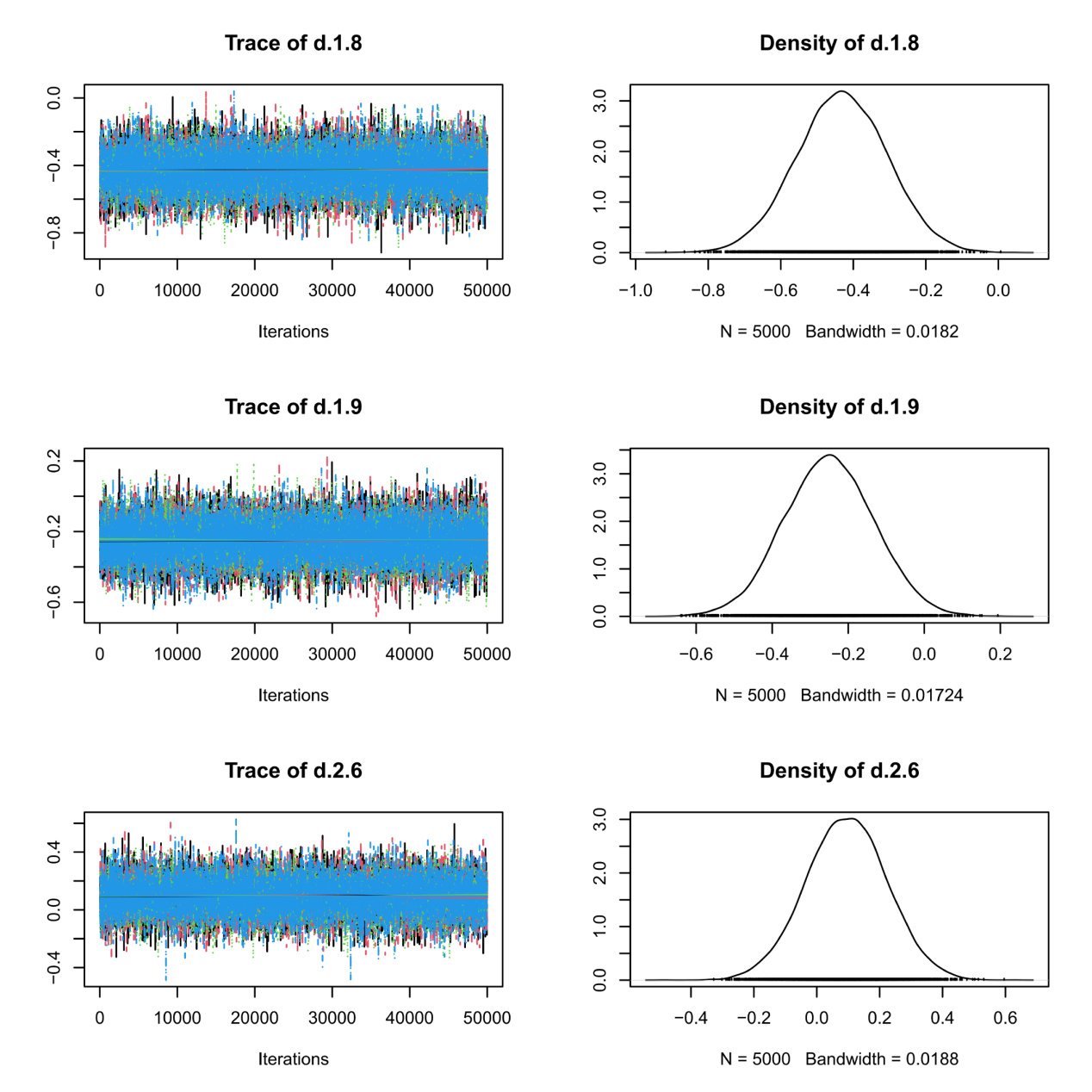


**Supplementary Figure 4.** Trace and density plots for the overall survival (OS) model in the non-brain metastasis subgroup.


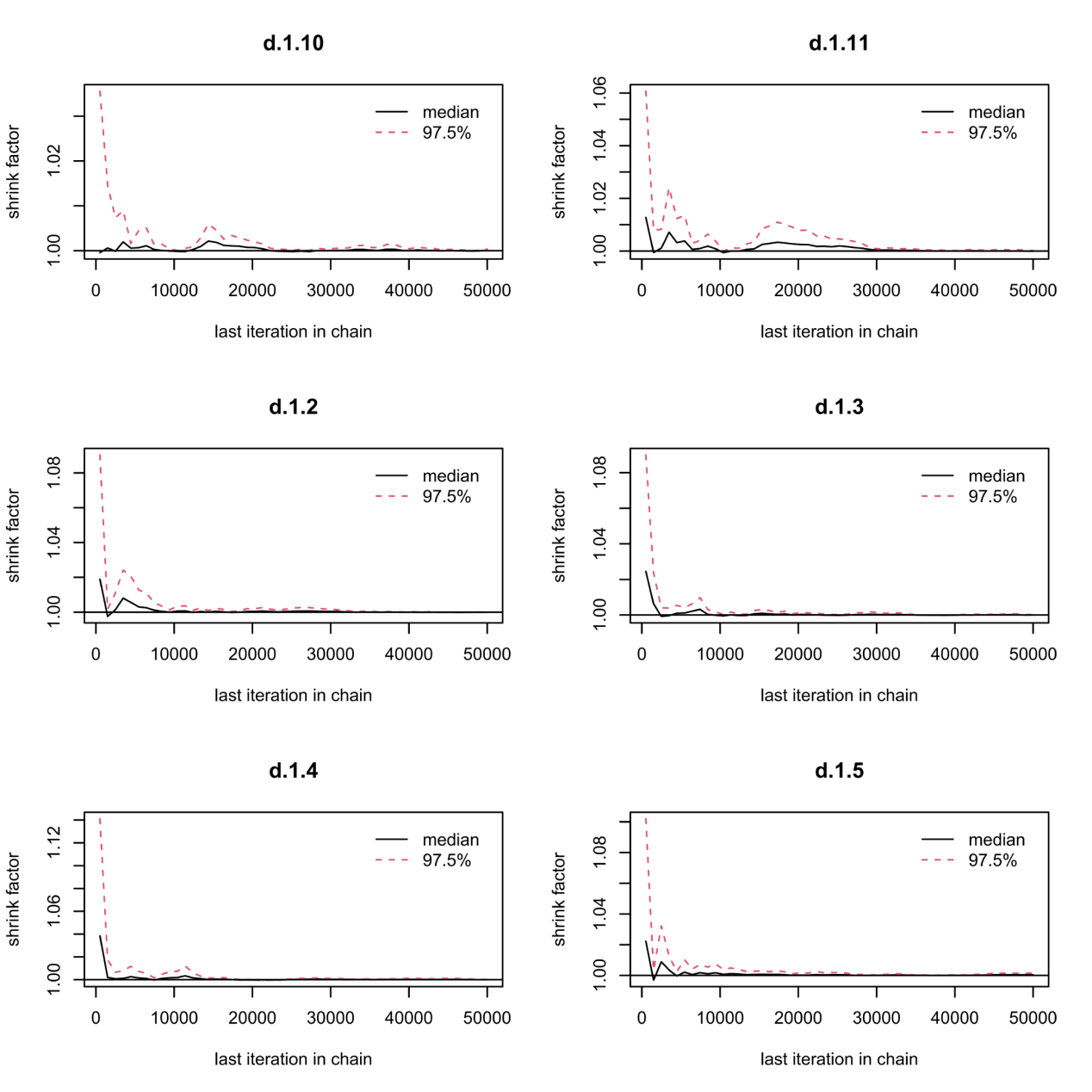


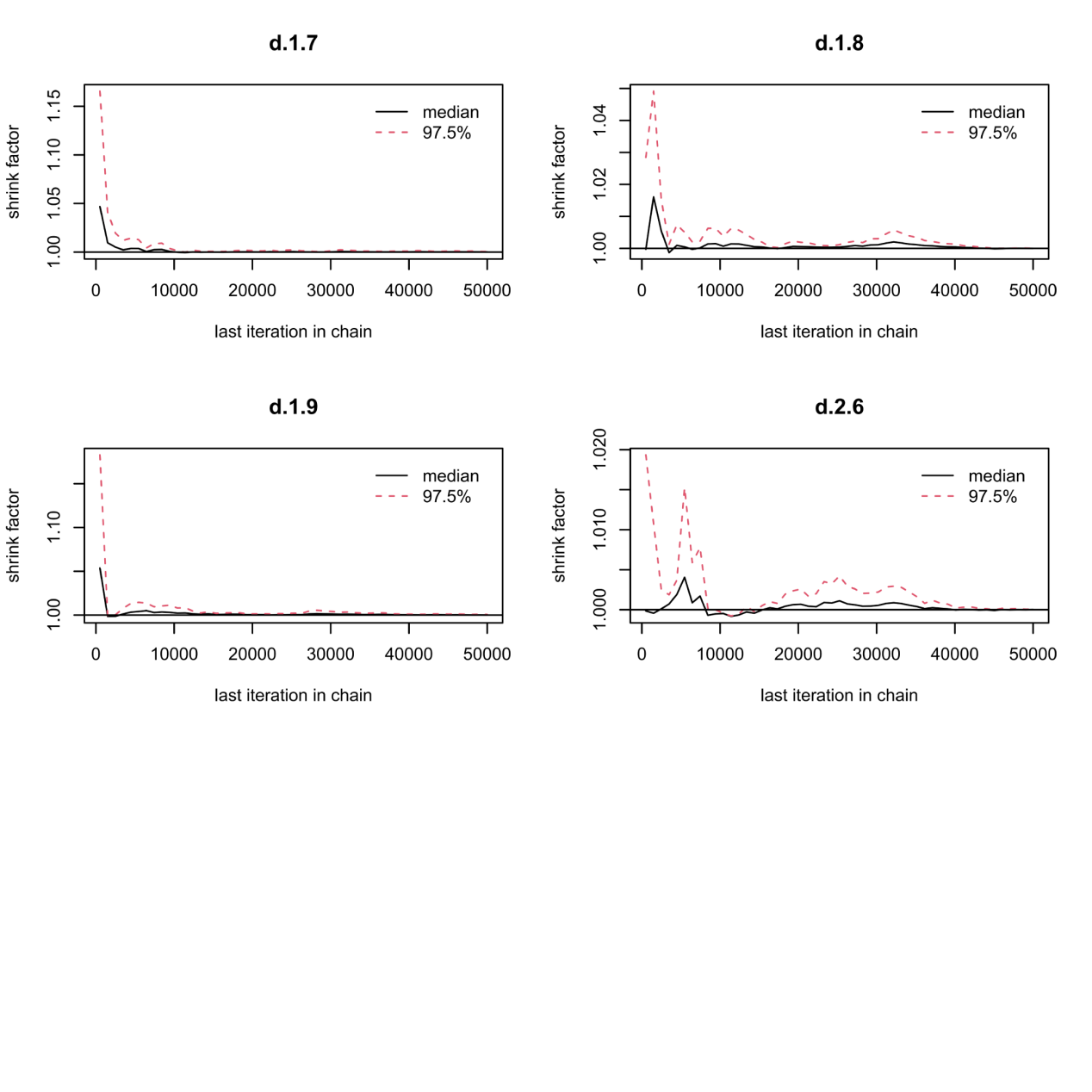


**Supplementary Figure 5.** Convergence diagnostics for the progression-free survival (PFS) model in the brain metastasis subgroup.


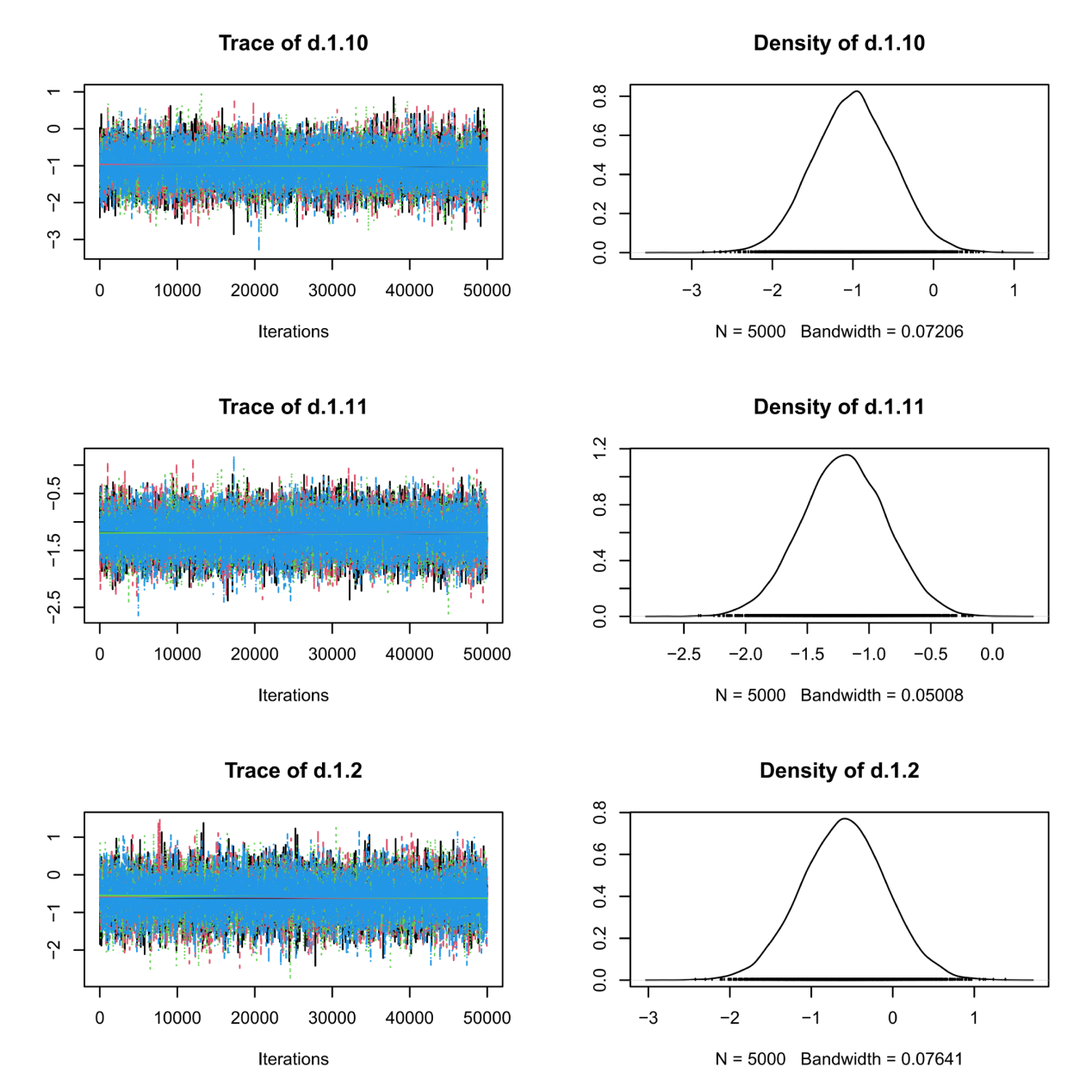


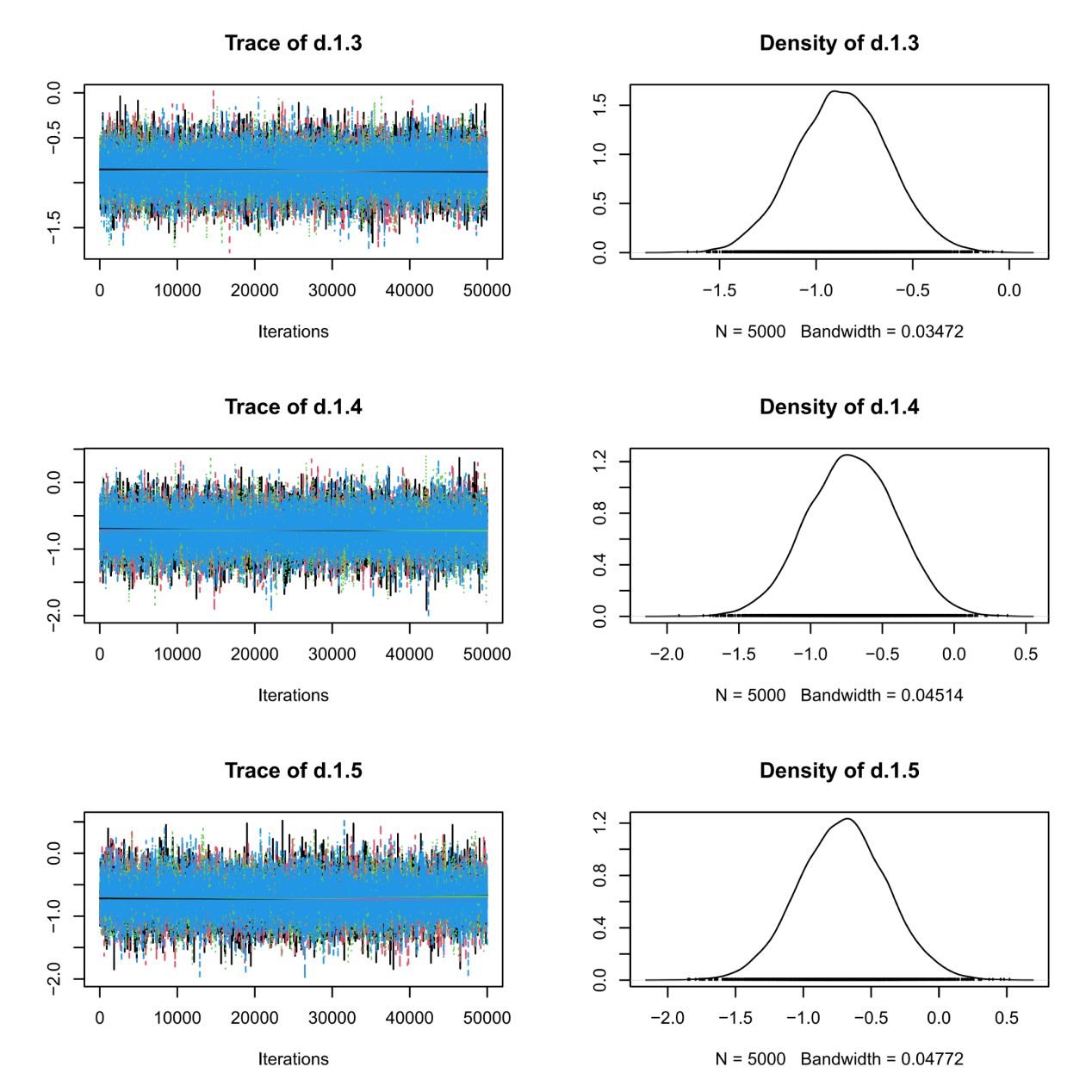


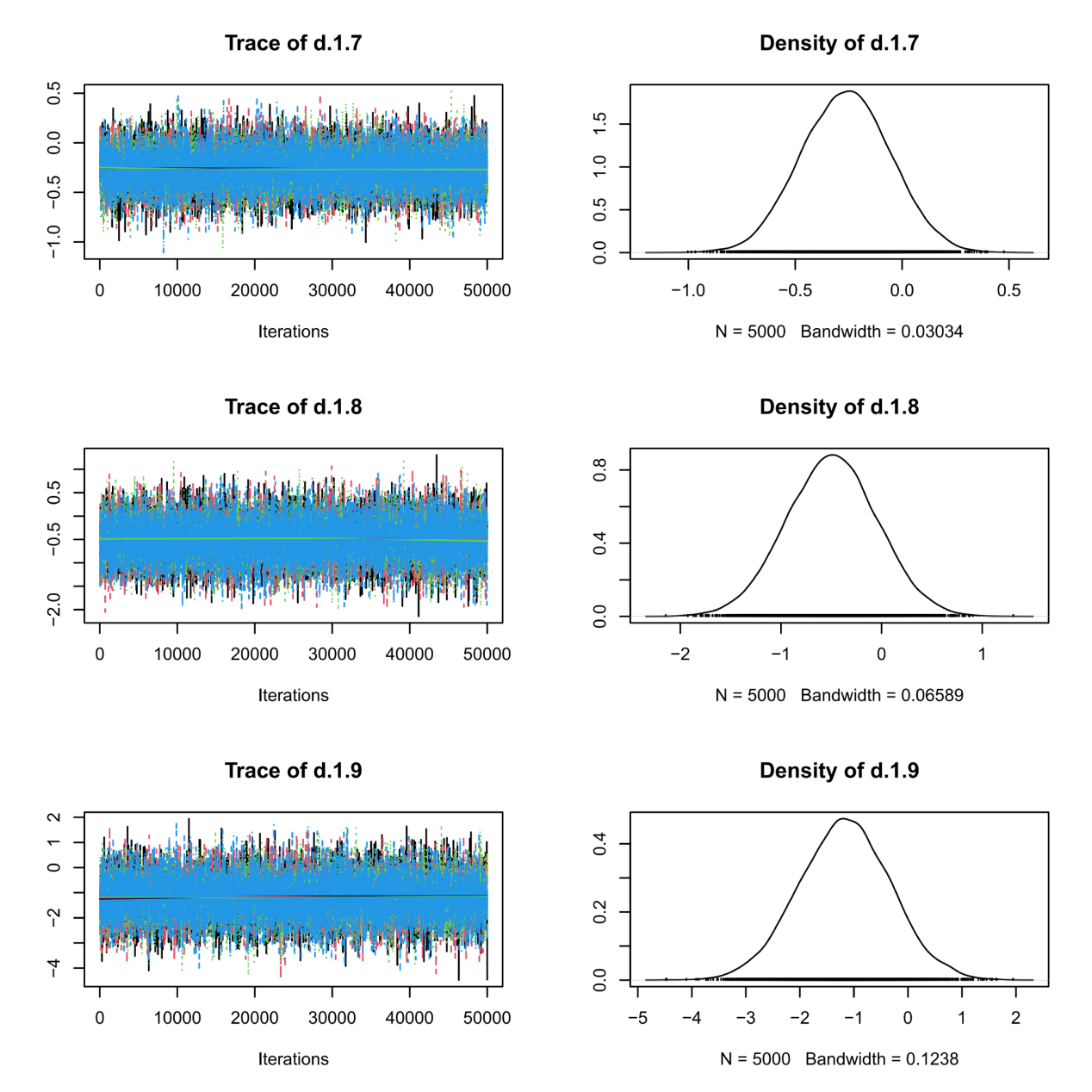


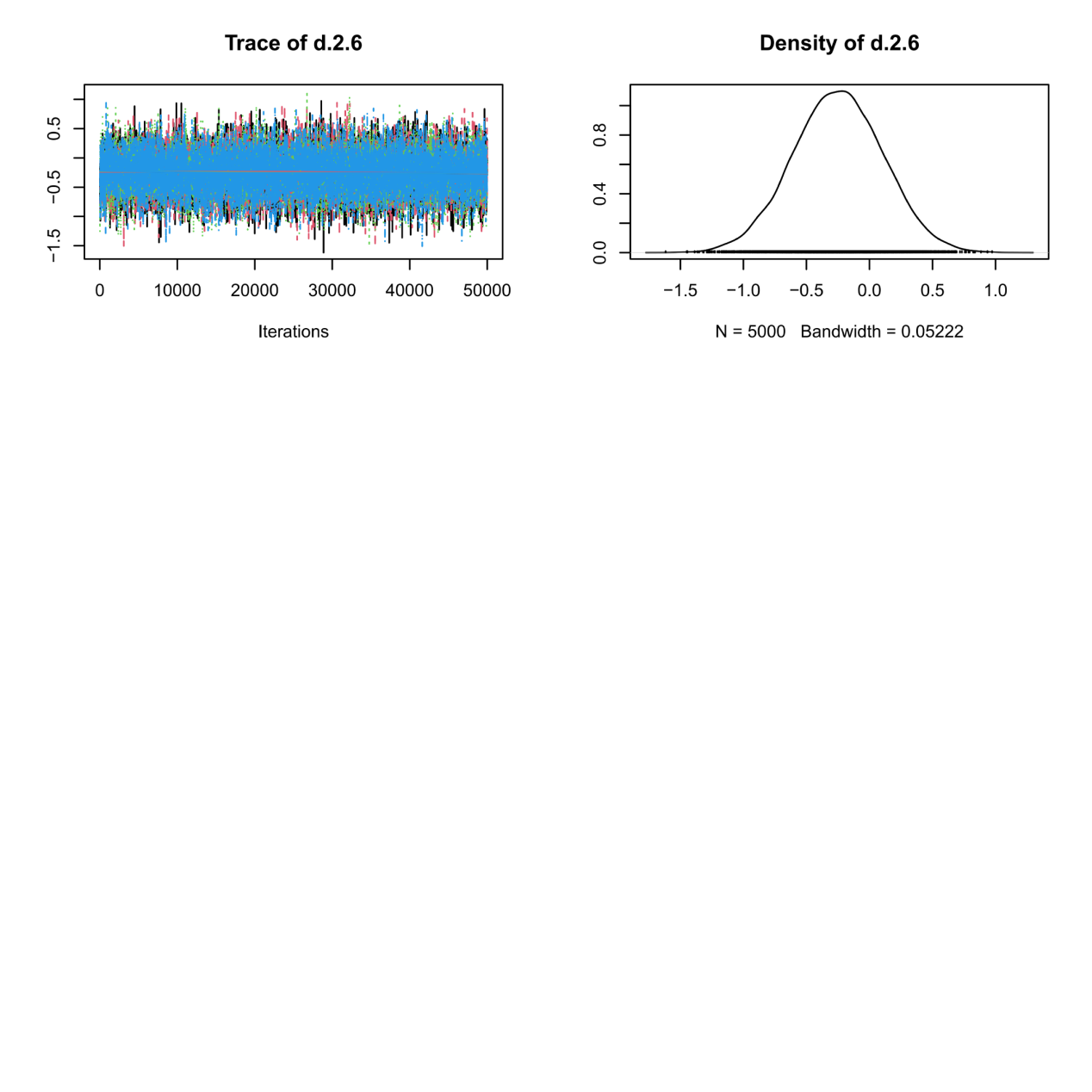


**Supplementary Figure 6.** Trace and density plots for the progression-free survival (PFS) model in the brain metastasis subgroup.


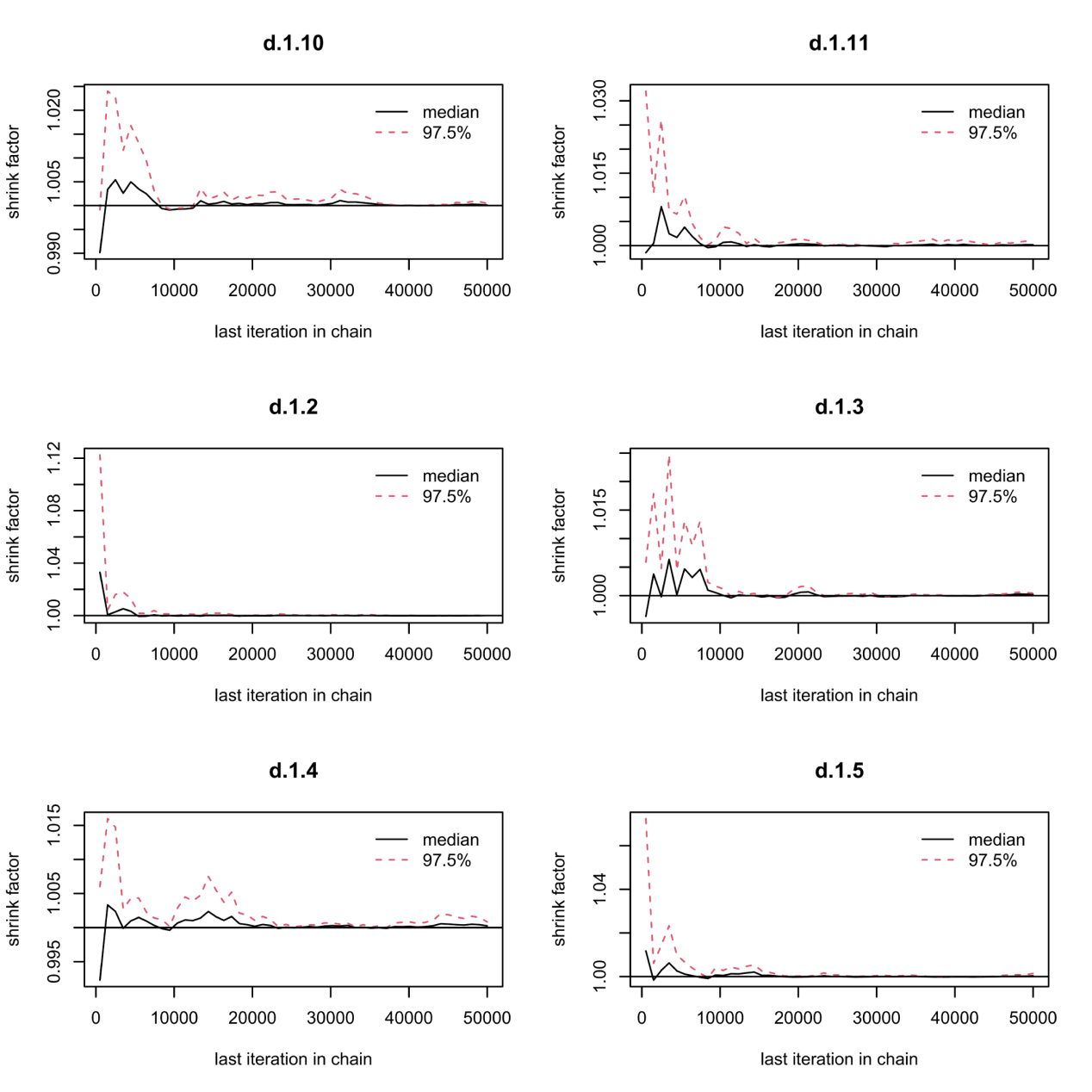


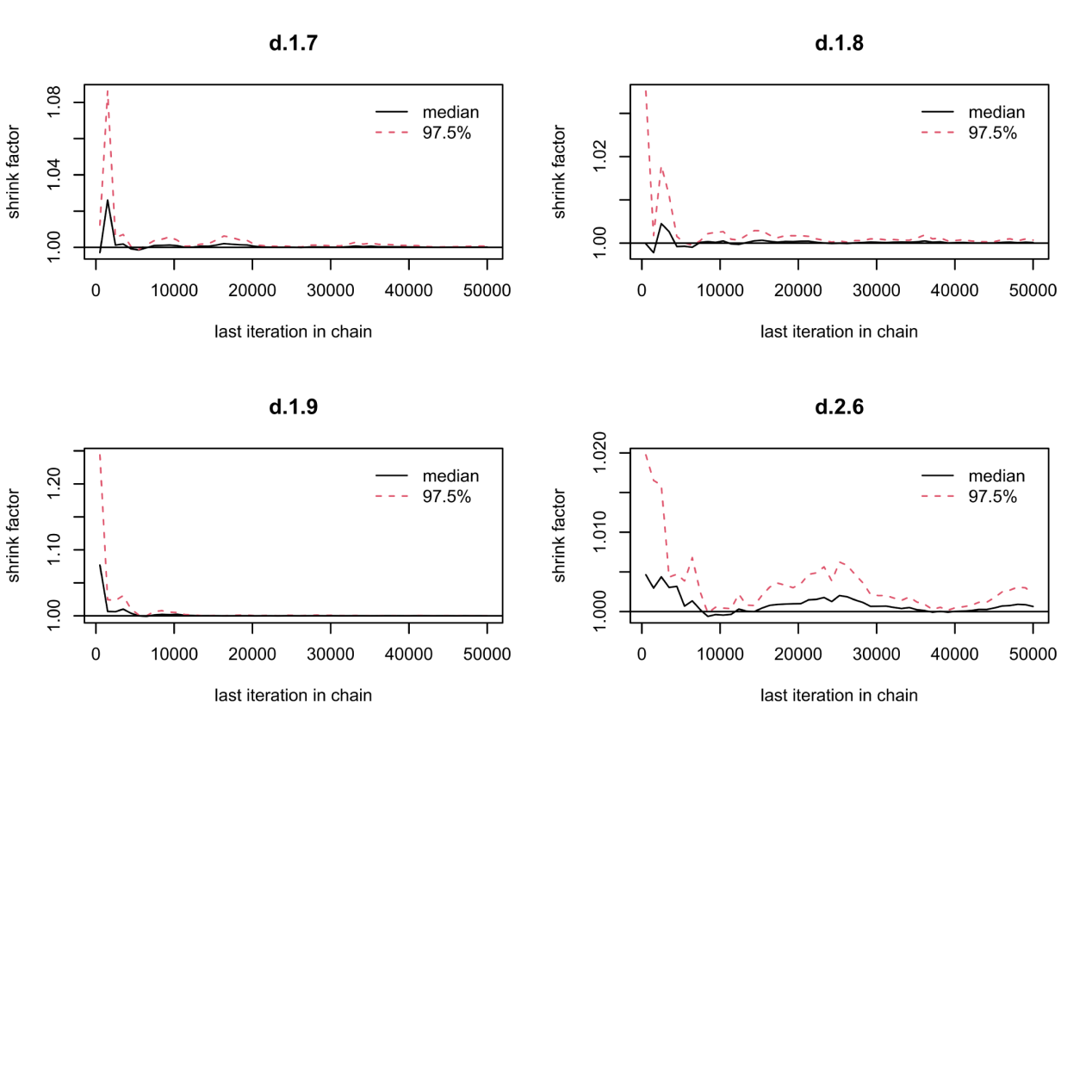


**Supplementary Figure 7.** Convergence diagnostics for the progression-free survival (PFS) model in the non-brain metastasis subgroup.


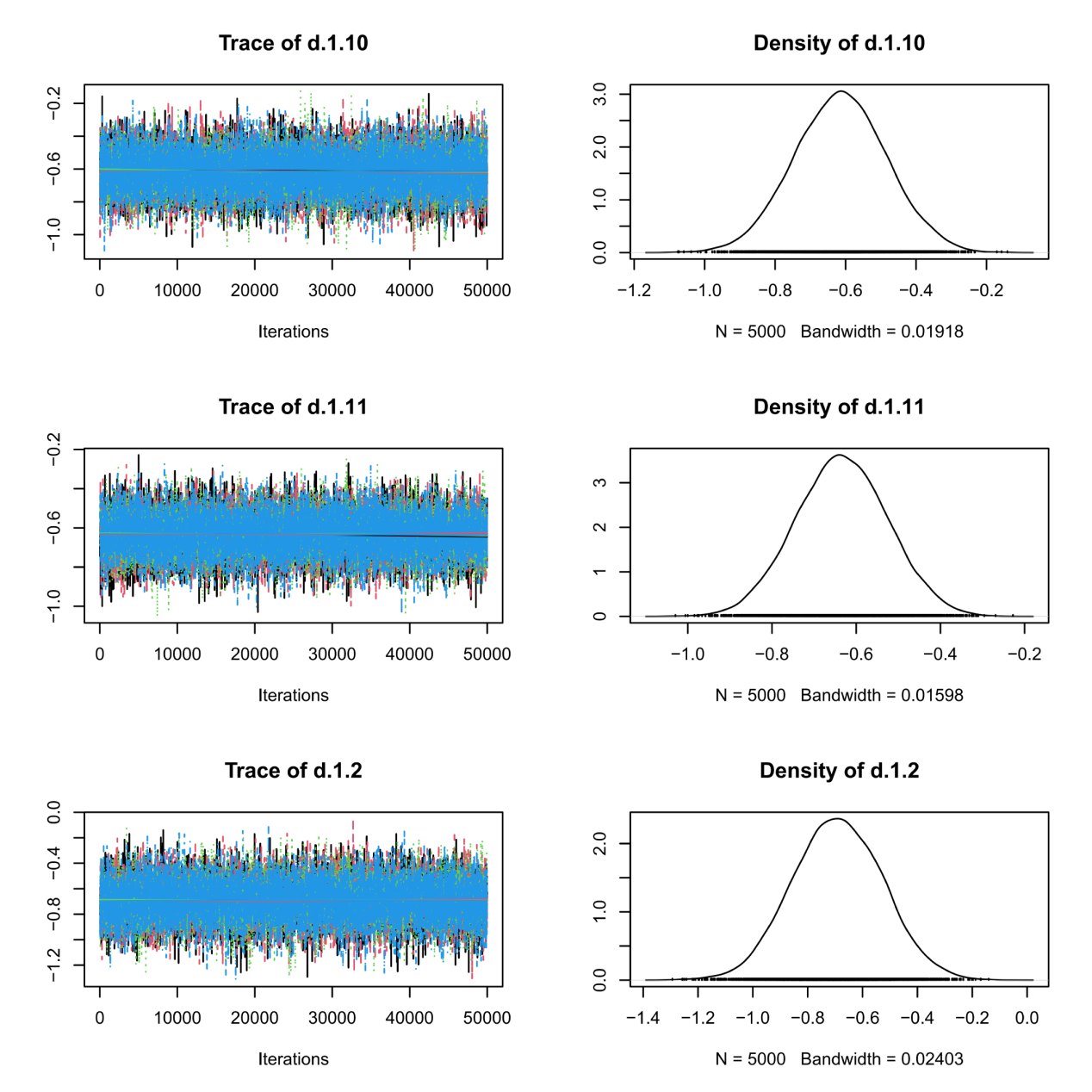


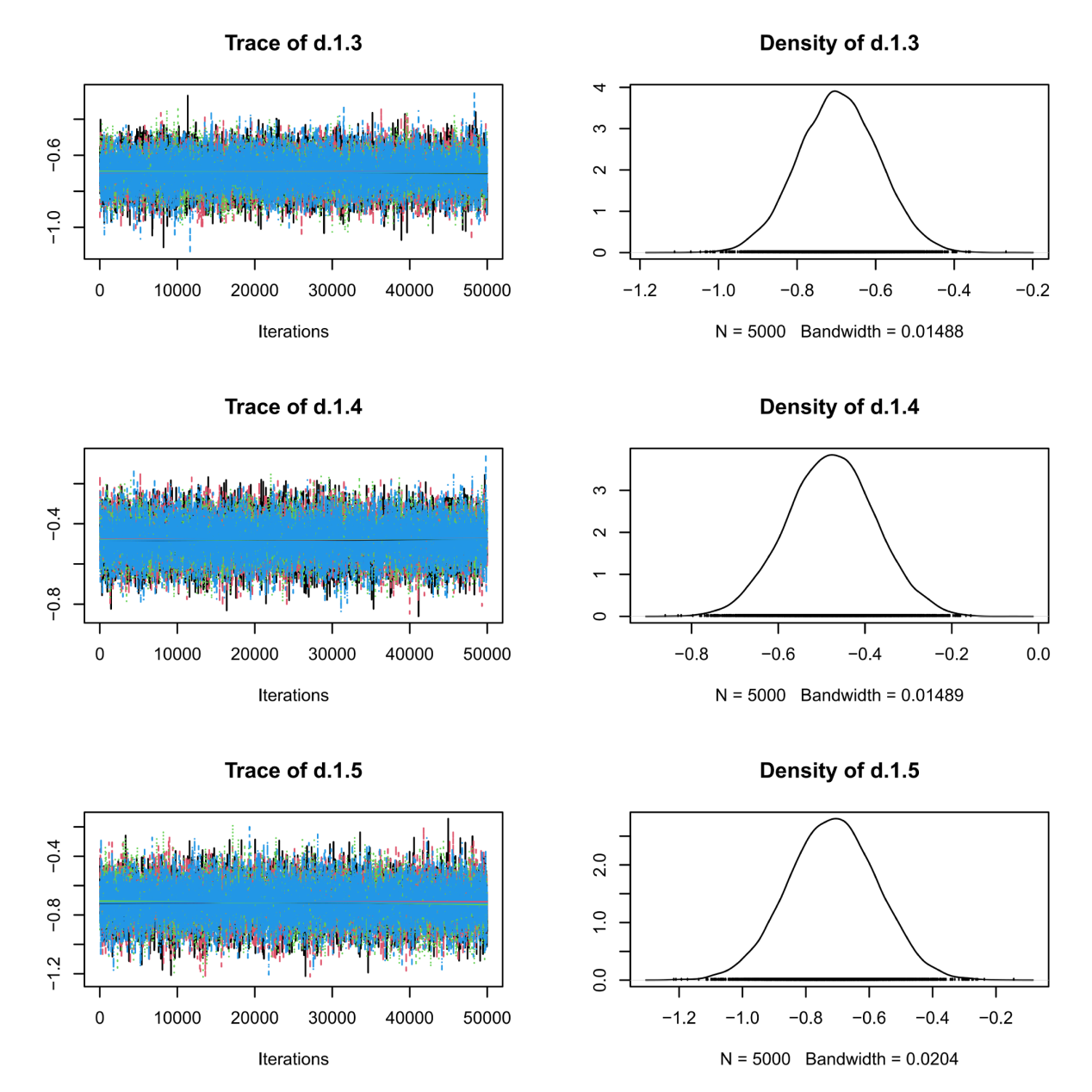


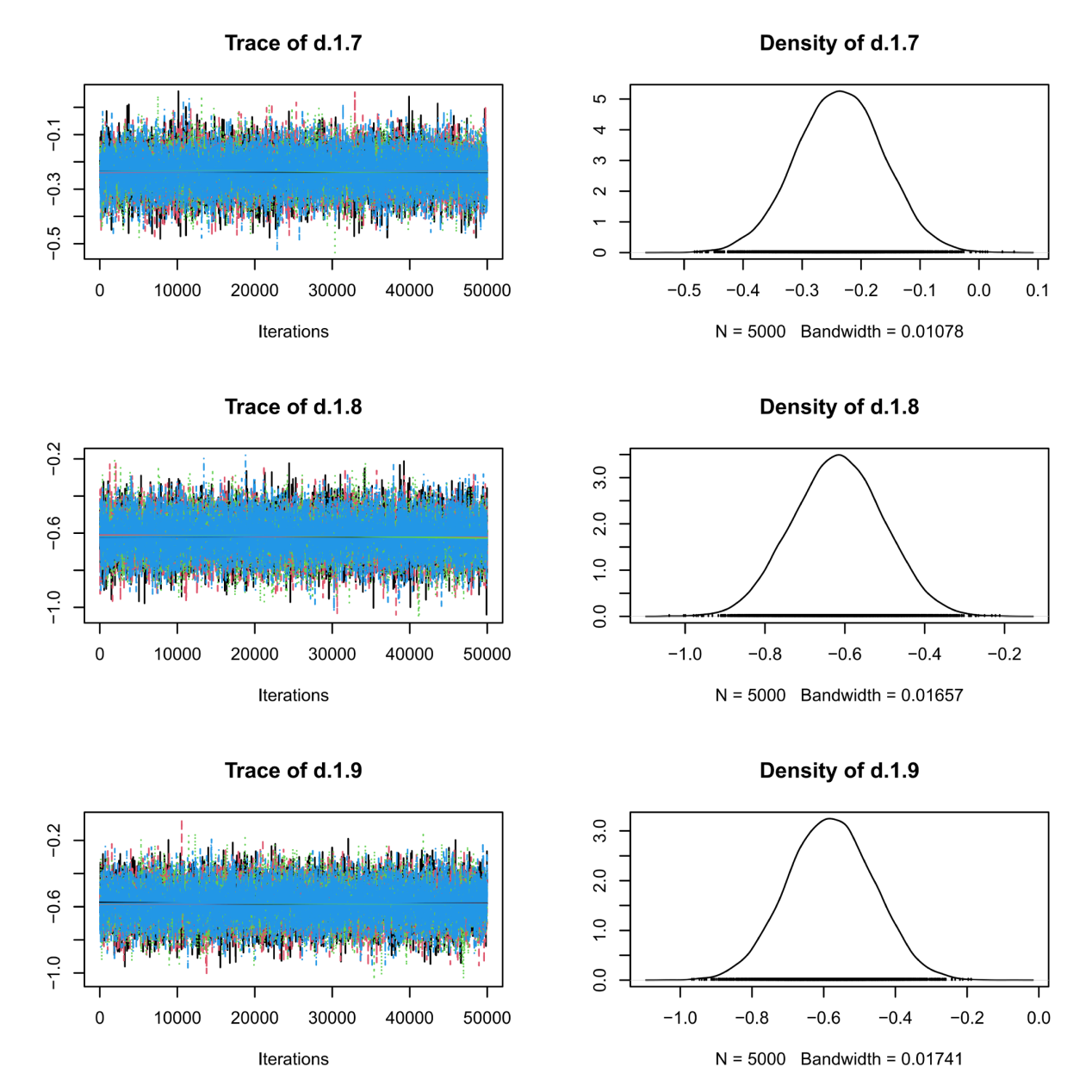


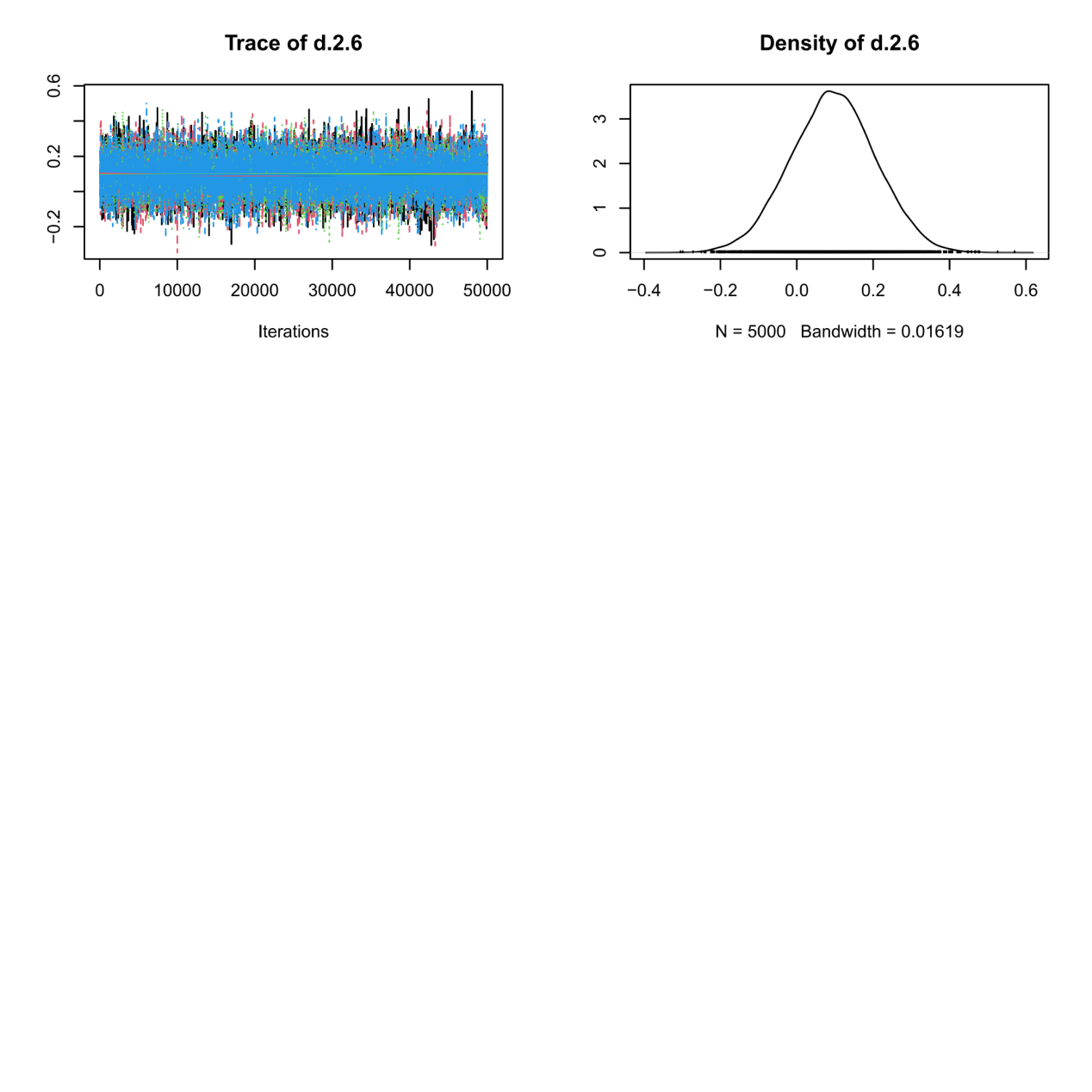


**Supplementary Figure 8.** Trace and density plots for the progression-free survival (PFS) model in the non-brain metastasis subgroup.

| **Supplementary Table 3. Certainty of evidence for overall survival in NSCLC patients with brain metastases assessed by CINeMA.** | | | | | | | | |
| --- | --- | --- | --- | --- | --- | --- | --- | --- |
| **Comparison** | **Studies(n)** | **Within-study bias** | **Reporting bias** | **Indirectness** | **Imprecision** | **Heterogeneity** | **Incoherence** | **Confidence rating** |
| Camre-chemo:chemo | 1 | No concerns | Low risk | No concerns | No concerns | Some concerns | Some concerns | Low |
| Cemip-chemo:chemo | 1 | No concerns | Low risk | No concerns | No concerns | Some concerns | Some concerns | Low |
| Pembro:Pembro-ipi | 1 | No concerns | Low risk | No concerns | Some concerns | No concerns | Some concerns | Low |
| chemo:Pembro-chemo | 1 | No concerns | Low risk | No concerns | No concerns | Some concerns | Some concerns | Low |
| chemo:Sugem-chemo | 1 | No concerns | Low risk | No concerns | No concerns | Some concerns | Some concerns | Low |
| Atezo:Sinti-chemo | 0 | No concerns | Low risk | No concerns | Some concerns | No concerns | Some concerns | Low |
| Atezo:Sugem-chemo | 0 | No concerns | Low risk | No concerns | Some concerns | No concerns | Some concerns | Low |
| Camre-chemo:Cemip-chemo | 0 | No concerns | Low risk | No concerns | Some concerns | No concerns | Some concerns | Low |
| Camre-chemo:Pembro-chemo | 0 | No concerns | Low risk | No concerns | Some concerns | No concerns | Some concerns | Low |
| Camre-chemo:Pembro-ipi | 0 | No concerns | Low risk | No concerns | Some concerns | No concerns | Some concerns | Low |
| Camre-chemo:Sinti-chemo | 0 | No concerns | Low risk | No concerns | Some concerns | No concerns | Some concerns | Low |
| Camre-chemo:Sugem-chemo | 0 | No concerns | Low risk | No concerns | Some concerns | No concerns | Some concerns | Low |
| Cemip:Cemip-chemo | 0 | No concerns | Low risk | No concerns | Some concerns | No concerns | Some concerns | Low |
| Cemip:Pembro-chemo | 0 | No concerns | Low risk | No concerns | Some concerns | No concerns | Some concerns | Low |
| Cemip:Sinti-chemo | 0 | No concerns | Low risk | No concerns | Some concerns | No concerns | Some concerns | Low |
| Cemip:Sugem-chemo | 0 | No concerns | Low risk | No concerns | Some concerns | No concerns | Some concerns | Low |
| Cemip-chemo:Nivo-ipi | 0 | No concerns | Low risk | No concerns | Some concerns | No concerns | Some concerns | Low |
| Cemip-chemo:Pembro | 0 | No concerns | Low risk | No concerns | Some concerns | No concerns | Some concerns | Low |
| Cemip-chemo:Pembro-chemo | 0 | No concerns | Low risk | No concerns | Some concerns | No concerns | Some concerns | Low |
| Cemip-chemo:Pembro-ipi | 0 | No concerns | Low risk | No concerns | Some concerns | No concerns | Some concerns | Low |
| Cemip-chemo:Sinti-chemo | 0 | No concerns | Low risk | No concerns | Some concerns | No concerns | Some concerns | Low |
| Cemip-chemo:Sugem-chemo | 0 | No concerns | Low risk | No concerns | Some concerns | No concerns | Some concerns | Low |
| Durva-chemo:Sinti-chemo | 0 | No concerns | Low risk | No concerns | Some concerns | No concerns | Some concerns | Low |
| Nivo-ipi:Pembro-chemo | 0 | No concerns | Low risk | No concerns | Some concerns | No concerns | Some concerns | Low |
| Nivo-ipi:Sinti-chemo | 0 | No concerns | Low risk | No concerns | Some concerns | No concerns | Some concerns | Low |
| Nivo-ipi:Sugem-chemo | 0 | No concerns | Low risk | No concerns | Some concerns | No concerns | Some concerns | Low |
| Pembro:Pembro-chemo | 0 | No concerns | Low risk | No concerns | Some concerns | No concerns | Some concerns | Low |
| Pembro:Sinti-chemo | 0 | No concerns | Low risk | No concerns | Some concerns | No concerns | Some concerns | Low |
| Pembro:Sugem-chemo | 0 | No concerns | Low risk | No concerns | Some concerns | No concerns | Some concerns | Low |
| Pembro-chemo:Pembro-ipi | 0 | No concerns | Low risk | No concerns | Some concerns | No concerns | Some concerns | Low |
| Pembro-chemo:Sinti-chemo | 0 | No concerns | Low risk | No concerns | Some concerns | No concerns | Some concerns | Low |
| Pembro-chemo:Sugem-chemo | 0 | No concerns | Low risk | No concerns | Some concerns | No concerns | Some concerns | Low |
| Pembro-ipi:Sinti-chemo | 0 | No concerns | Low risk | No concerns | Some concerns | No concerns | Some concerns | Low |
| Pembro-ipi:Sugem-chemo | 0 | No concerns | Low risk | No concerns | Some concerns | No concerns | Some concerns | Low |
| chemo:Pembro-ipi | 0 | No concerns | Low risk | No concerns | Some concerns | No concerns | Some concerns | Low |
| Sinti-chemo:Sugem-chemo | 0 | No concerns | Low risk | No concerns | Some concerns | No concerns | Some concerns | Low |
| Sinti-chemo:Treme-durva-chemo | 0 | No concerns | Low risk | No concerns | Some concerns | No concerns | Some concerns | Low |
| Atezo:chemo | 1 | Some concerns | Low risk | No concerns | Some concerns | No concerns | Some concerns | Very low |
| Cemip:chemo | 1 | Some concerns | Low risk | No concerns | Some concerns | Some concerns | Some concerns | Very low |
| chemo:Durva-chemo | 1 | Some concerns | Low risk | No concerns | Some concerns | No concerns | Some concerns | Very low |
| chemo:Nivo-ipi | 1 | Some concerns | Low risk | No concerns | No concerns | Some concerns | Some concerns | Very low |
| chemo:Pembro | 1 | Some concerns | Low risk | No concerns | Some concerns | No concerns | Some concerns | Very low |
| chemo:Sinti-chemo | 1 | No concerns | Low risk | No concerns | Some concerns | Some concerns | Some concerns | Very low |
| chemo:Treme-durva-chemo | 1 | Some concerns | Low risk | No concerns | Some concerns | No concerns | Some concerns | Very low |
| Atezo:Camre-chemo | 0 | Some concerns | Low risk | No concerns | Some concerns | Some concerns | Some concerns | Very low |
| Atezo:Cemip | 0 | Some concerns | Low risk | No concerns | Some concerns | No concerns | Some concerns | Very low |
| Atezo:Cemip-chemo | 0 | No concerns | Low risk | No concerns | Some concerns | Some concerns | Some concerns | Very low |
| Atezo:Durva-chemo | 0 | Some concerns | Low risk | No concerns | Some concerns | No concerns | Some concerns | Very low |
| Atezo:Nivo-ipi | 0 | Some concerns | Low risk | No concerns | Some concerns | No concerns | Some concerns | Very low |
| Atezo:Pembro | 0 | Some concerns | Low risk | No concerns | Some concerns | No concerns | Some concerns | Very low |
| Atezo:Pembro-chemo | 0 | No concerns | Low risk | No concerns | Some concerns | Some concerns | Some concerns | Very low |
| Atezo:Pembro-ipi | 0 | Some concerns | Low risk | No concerns | Some concerns | No concerns | Some concerns | Very low |
| Atezo:Treme-durva-chemo | 0 | Some concerns | Low risk | No concerns | Some concerns | No concerns | Some concerns | Very low |
| Camre-chemo:Cemip | 0 | Some concerns | Low risk | No concerns | Some concerns | No concerns | Some concerns | Very low |
| Camre-chemo:Durva-chemo | 0 | Some concerns | Low risk | No concerns | Some concerns | Some concerns | Some concerns | Very low |
| Camre-chemo:Nivo-ipi | 0 | Some concerns | Low risk | No concerns | Some concerns | No concerns | Some concerns | Very low |
| Camre-chemo:Pembro | 0 | Some concerns | Low risk | No concerns | Some concerns | No concerns | Some concerns | Very low |
| Camre-chemo:Treme-durva-chemo | 0 | Some concerns | Low risk | No concerns | Some concerns | Some concerns | Some concerns | Very low |
| Cemip:Durva-chemo | 0 | Some concerns | Low risk | No concerns | Some concerns | No concerns | Some concerns | Very low |
| Cemip:Nivo-ipi | 0 | Some concerns | Low risk | No concerns | Some concerns | No concerns | Some concerns | Very low |
| Cemip:Pembro | 0 | Some concerns | Low risk | No concerns | Some concerns | No concerns | Some concerns | Very low |
| Cemip:Pembro-ipi | 0 | Some concerns | Low risk | No concerns | Some concerns | No concerns | Some concerns | Very low |
| Cemip:Treme-durva-chemo | 0 | Some concerns | Low risk | No concerns | Some concerns | No concerns | Some concerns | Very low |
| Cemip-chemo:Durva-chemo | 0 | No concerns | Low risk | No concerns | Some concerns | Some concerns | Some concerns | Very low |
| Cemip-chemo:Treme-durva-chemo | 0 | No concerns | Low risk | No concerns | Some concerns | Some concerns | Some concerns | Very low |
| Durva-chemo:Nivo-ipi | 0 | Some concerns | Low risk | No concerns | Some concerns | No concerns | Some concerns | Very low |
| Durva-chemo:Pembro | 0 | Some concerns | Low risk | No concerns | Some concerns | No concerns | Some concerns | Very low |
| Durva-chemo:Pembro-chemo | 0 | No concerns | Low risk | No concerns | Some concerns | Some concerns | Some concerns | Very low |
| Durva-chemo:Pembro-ipi | 0 | Some concerns | Low risk | No concerns | Some concerns | No concerns | Some concerns | Very low |
| Durva-chemo:Sugem-chemo | 0 | No concerns | Low risk | No concerns | Some concerns | Some concerns | Some concerns | Very low |
| Durva-chemo:Treme-durva-chemo | 0 | Some concerns | Low risk | No concerns | Some concerns | No concerns | Some concerns | Very low |
| Nivo-ipi:Pembro | 0 | Some concerns | Low risk | No concerns | Some concerns | No concerns | Some concerns | Very low |
| Nivo-ipi:Pembro-ipi | 0 | Some concerns | Low risk | No concerns | Some concerns | No concerns | Some concerns | Very low |
| Nivo-ipi:Treme-durva-chemo | 0 | Some concerns | Low risk | No concerns | Some concerns | No concerns | Some concerns | Very low |
| Pembro:Treme-durva-chemo | 0 | Some concerns | Low risk | No concerns | Some concerns | No concerns | Some concerns | Very low |
| Pembro-chemo:Treme-durva-chemo | 0 | No concerns | Low risk | No concerns | Some concerns | Some concerns | Some concerns | Very low |
| Pembro-ipi:Treme-durva-chemo | 0 | Some concerns | Low risk | No concerns | Some concerns | No concerns | Some concerns | Very low |
| Sugem-chemo:Treme-durva-chemo | 0 | No concerns | Low risk | No concerns | Some concerns | Some concerns | Some concerns | Very low |

| **Supplementary Table 4. Certainty of evidence for overall survival in NSCLC patients without brain metastases assessed by CINeMA.** | | | | | | | | |
| --- | --- | --- | --- | --- | --- | --- | --- | --- |
| **Comparison** | **Studies(n)** | **Within-study bias** | **Reporting bias** | **Indirectness** | **Imprecision** | **Heterogeneity** | **Incoherence** | **Confidence rating** |
| Camre-chemo:chemo | 1 | No concerns | Low risk | No concerns | No concerns | Some concerns | Some concerns | Low |
| Cemip-chemo:chemo | 1 | No concerns | Low risk | No concerns | No concerns | Some concerns | Some concerns | Low |
| chemo:Pembro-chemo | 1 | No concerns | Low risk | No concerns | No concerns | Some concerns | Some concerns | Low |
| chemo:Sinti-chemo | 1 | No concerns | Low risk | No concerns | No concerns | Some concerns | Some concerns | Low |
| chemo:Sugem-chemo | 1 | No concerns | Low risk | No concerns | No concerns | Some concerns | Some concerns | Low |
| Atezo:Sugem-chemo | 0 | No concerns | Low risk | No concerns | Some concerns | No concerns | Some concerns | Low |
| Camre-chemo:Pembro-ipi | 0 | No concerns | Low risk | No concerns | Some concerns | No concerns | Some concerns | Low |
| Camre-chemo:Sugem-chemo | 0 | No concerns | Low risk | No concerns | Some concerns | No concerns | Some concerns | Low |
| Cemip:Cemip-chemo | 0 | No concerns | Low risk | No concerns | Some concerns | No concerns | Some concerns | Low |
| Cemip:Sinti-chemo | 0 | No concerns | Low risk | No concerns | Some concerns | No concerns | Some concerns | Low |
| Cemip:Sugem-chemo | 0 | No concerns | Low risk | No concerns | Some concerns | No concerns | Some concerns | Low |
| Cemip-chemo:Pembro | 0 | No concerns | Low risk | No concerns | Some concerns | No concerns | Some concerns | Low |
| Cemip-chemo:Pembro-ipi | 0 | No concerns | Low risk | No concerns | Some concerns | No concerns | Some concerns | Low |
| Cemip-chemo:Sinti-chemo | 0 | No concerns | Low risk | No concerns | Some concerns | No concerns | Some concerns | Low |
| Cemip-chemo:Sugem-chemo | 0 | No concerns | Low risk | No concerns | Some concerns | No concerns | Some concerns | Low |
| Durva-chemo:Pembro-chemo | 0 | No concerns | Low risk | No concerns | No concerns | Some concerns | Some concerns | Low |
| Nivo-ipi:Pembro-chemo | 0 | No concerns | Low risk | No concerns | No concerns | Some concerns | Some concerns | Low |
| Nivo-ipi:Sugem-chemo | 0 | No concerns | Low risk | No concerns | Some concerns | No concerns | Some concerns | Low |
| Pembro:Pembro-chemo | 0 | No concerns | Low risk | No concerns | Some concerns | No concerns | Some concerns | Low |
| Pembro:Sinti-chemo | 0 | No concerns | Low risk | No concerns | Some concerns | No concerns | Some concerns | Low |
| Pembro:Sugem-chemo | 0 | No concerns | Low risk | No concerns | Some concerns | No concerns | Some concerns | Low |
| Pembro-chemo:Pembro-ipi | 0 | No concerns | Low risk | No concerns | Some concerns | No concerns | Some concerns | Low |
| Pembro-ipi:Sinti-chemo | 0 | No concerns | Low risk | No concerns | Some concerns | No concerns | Some concerns | Low |
| Pembro-ipi:Sugem-chemo | 0 | No concerns | Low risk | No concerns | Some concerns | No concerns | Some concerns | Low |
| Pembro-chemo:Sinti-chemo | 0 | No concerns | Low risk | No concerns | Some concerns | No concerns | Some concerns | Low |
| Sugem-chemo:Treme-durva-chemo | 0 | No concerns | Low risk | No concerns | Some concerns | No concerns | Some concerns | Low |
| Atezo:chemo | 1 | Some concerns | Low risk | No concerns | No concerns | Some concerns | Some concerns | Very low |
| Cemip:chemo | 1 | Some concerns | Low risk | No concerns | No concerns | Some concerns | Some concerns | Very low |
| chemo:Durva-chemo | 1 | Some concerns | Low risk | No concerns | No concerns | Some concerns | Some concerns | Very low |
| chemo:Nivo-ipi | 1 | Some concerns | Low risk | No concerns | No concerns | Some concerns | Some concerns | Very low |
| Pembro:Pembro-ipi | 1 | No concerns | Low risk | No concerns | Some concerns | Some concerns | Some concerns | Very low |
| chemo:Pembro | 1 | Some concerns | Low risk | No concerns | No concerns | Some concerns | Some concerns | Very low |
| chemo:Treme-durva-chemo | 1 | Some concerns | Low risk | No concerns | No concerns | Some concerns | Some concerns | Very low |
| Atezo:Camre-chemo | 0 | Some concerns | Low risk | No concerns | Some concerns | Some concerns | Some concerns | Very low |
| Atezo:Cemip | 0 | Some concerns | Low risk | No concerns | Some concerns | No concerns | Some concerns | Very low |
| Atezo:Cemip-chemo | 0 | No concerns | Low risk | No concerns | Some concerns | Some concerns | Some concerns | Very low |
| Atezo:Durva-chemo | 0 | Some concerns | Low risk | No concerns | Some concerns | No concerns | Some concerns | Very low |
| Atezo:Nivo-ipi | 0 | Some concerns | Low risk | No concerns | Some concerns | No concerns | Some concerns | Very low |
| Atezo:Pembro | 0 | Some concerns | Low risk | No concerns | Some concerns | Some concerns | Some concerns | Very low |
| Atezo:Pembro-ipi | 0 | Some concerns | Low risk | No concerns | Some concerns | No concerns | Some concerns | Very low |
| Atezo:Pembro-chemo | 0 | No concerns | Low risk | No concerns | Some concerns | Some concerns | Some concerns | Very low |
| Atezo:Sinti-chemo | 0 | No concerns | Low risk | No concerns | Some concerns | Some concerns | Some concerns | Very low |
| Atezo:Treme-durva-chemo | 0 | Some concerns | Low risk | No concerns | Some concerns | No concerns | Some concerns | Very low |
| Camre-chemo:Cemip | 0 | Some concerns | Low risk | No concerns | Some concerns | No concerns | Some concerns | Very low |
| Camre-chemo:Cemip-chemo | 0 | No concerns | Low risk | No concerns | Some concerns | Some concerns | Some concerns | Very low |
| Camre-chemo:Durva-chemo | 0 | Some concerns | Low risk | No concerns | Some concerns | Some concerns | Some concerns | Very low |
| Camre-chemo:Nivo-ipi | 0 | Some concerns | Low risk | No concerns | Some concerns | Some concerns | Some concerns | Very low |
| Camre-chemo:Pembro | 0 | Some concerns | Low risk | No concerns | Some concerns | No concerns | Some concerns | Very low |
| Camre-chemo:Pembro-chemo | 0 | No concerns | Low risk | No concerns | Some concerns | Some concerns | Some concerns | Very low |
| Camre-chemo:Sinti-chemo | 0 | No concerns | Low risk | No concerns | Some concerns | Some concerns | Some concerns | Very low |
| Camre-chemo:Treme-durva-chemo | 0 | Some concerns | Low risk | No concerns | Some concerns | No concerns | Some concerns | Very low |
| Cemip:Durva-chemo | 0 | Some concerns | Low risk | No concerns | Some concerns | Some concerns | Some concerns | Very low |
| Cemip:Nivo-ipi | 0 | Some concerns | Low risk | No concerns | Some concerns | No concerns | Some concerns | Very low |
| Cemip:Pembro | 0 | Some concerns | Low risk | No concerns | Some concerns | No concerns | Some concerns | Very low |
| Cemip:Pembro-ipi | 0 | Some concerns | Low risk | No concerns | Some concerns | No concerns | Some concerns | Very low |
| Cemip:Pembro-chemo | 0 | No concerns | Low risk | No concerns | Some concerns | Some concerns | Some concerns | Very low |
| Cemip:Treme-durva-chemo | 0 | Some concerns | Low risk | No concerns | Some concerns | No concerns | Some concerns | Very low |
| Cemip-chemo:Durva-chemo | 0 | No concerns | Low risk | No concerns | Some concerns | Some concerns | Some concerns | Very low |
| Cemip-chemo:Nivo-ipi | 0 | No concerns | Low risk | No concerns | Some concerns | Some concerns | Some concerns | Very low |
| Cemip-chemo:Pembro-chemo | 0 | No concerns | Low risk | No concerns | Some concerns | Some concerns | Some concerns | Very low |
| Cemip-chemo:Treme-durva-chemo | 0 | No concerns | Low risk | No concerns | Some concerns | Some concerns | Some concerns | Very low |
| Durva-chemo:Nivo-ipi | 0 | Some concerns | Low risk | No concerns | Some concerns | Some concerns | Some concerns | Very low |
| Durva-chemo:Pembro | 0 | Some concerns | Low risk | No concerns | Some concerns | Some concerns | Some concerns | Very low |
| Durva-chemo:Pembro-ipi | 0 | Some concerns | Low risk | No concerns | Some concerns | No concerns | Some concerns | Very low |
| Durva-chemo:Sinti-chemo | 0 | No concerns | Low risk | No concerns | Some concerns | Some concerns | Some concerns | Very low |
| Durva-chemo:Sugem-chemo | 0 | No concerns | Low risk | No concerns | Some concerns | Some concerns | Some concerns | Very low |
| Durva-chemo:Treme-durva-chemo | 0 | Some concerns | Low risk | No concerns | Some concerns | Some concerns | Some concerns | Very low |
| Nivo-ipi:Pembro | 0 | Some concerns | Low risk | No concerns | Some concerns | Some concerns | Some concerns | Very low |
| Nivo-ipi:Pembro-ipi | 0 | Some concerns | Low risk | No concerns | Some concerns | No concerns | Some concerns | Very low |
| Nivo-ipi:Sinti-chemo | 0 | No concerns | Low risk | No concerns | Some concerns | Some concerns | Some concerns | Very low |
| Nivo-ipi:Treme-durva-chemo | 0 | Some concerns | Low risk | No concerns | Some concerns | Some concerns | Some concerns | Very low |
| Pembro:Treme-durva-chemo | 0 | Some concerns | Low risk | No concerns | Some concerns | No concerns | Some concerns | Very low |
| Pembro-ipi:Treme-durva-chemo | 0 | Some concerns | Low risk | No concerns | Some concerns | No concerns | Some concerns | Very low |
| chemo:Pembro-ipi | 0 | No concerns | Low risk | No concerns | Some concerns | Some concerns | Some concerns | Very low |
| Pembro-chemo:Sugem-chemo | 0 | No concerns | Low risk | No concerns | Some concerns | Some concerns | Some concerns | Very low |
| Pembro-chemo:Treme-durva-chemo | 0 | No concerns | Low risk | No concerns | Some concerns | Some concerns | Some concerns | Very low |
| Sinti-chemo:Sugem-chemo | 0 | No concerns | Low risk | No concerns | Some concerns | Some concerns | Some concerns | Very low |
| Sinti-chemo:Treme-durva-chemo | 0 | No concerns | Low risk | No concerns | Some concerns | Some concerns | Some concerns | Very low |

| **Supplementary Table 5. Certainty of evidence for progression-free survival in NSCLC patients with brain metastases assessed by CINeMA.** | | | | | | | | |
| --- | --- | --- | --- | --- | --- | --- | --- | --- |
| **Comparison** | **Studies(n)** | **Within-study bias** | **Reporting bias** | **Indirectness** | **Imprecision** | **Heterogeneity** | **Incoherence** | **Confidence rating** |
| Cemip-chemo:chemo | 1 | No concerns | Low risk | No concerns | Some concerns | No concerns | Some concerns | Low |
| Pembro:Pembro-ipi | 1 | No concerns | Low risk | No concerns | Some concerns | No concerns | Some concerns | Low |
| chemo:Pembro-chemo | 1 | No concerns | Low risk | No concerns | No concerns | Some concerns | Some concerns | Low |
| chemo:Serpl-chemo | 1 | No concerns | Low risk | No concerns | No concerns | Some concerns | Some concerns | Low |
| chemo:Sinti-chemo | 1 | No concerns | Low risk | No concerns | No concerns | Some concerns | Some concerns | Low |
| chemo:Sugem-chemo | 1 | No concerns | Low risk | No concerns | No concerns | Some concerns | Some concerns | Low |
| Camre-chemo:Cemip-chemo | 0 | No concerns | Low risk | No concerns | Some concerns | No concerns | Some concerns | Low |
| Camre-chemo:Pembro-chemo | 0 | No concerns | Low risk | No concerns | Some concerns | No concerns | Some concerns | Low |
| Camre-chemo:Pembro-ipi | 0 | No concerns | Low risk | No concerns | Some concerns | No concerns | Some concerns | Low |
| Camre-chemo:Serpl-chemo | 0 | No concerns | Low risk | No concerns | Some concerns | No concerns | Some concerns | Low |
| Camre-chemo:Sinti-chemo | 0 | No concerns | Low risk | No concerns | Some concerns | No concerns | Some concerns | Low |
| Camre-chemo:Sugem-chemo | 0 | No concerns | Low risk | No concerns | Some concerns | No concerns | Some concerns | Low |
| Cemip:Cemip-chemo | 0 | No concerns | Low risk | No concerns | Some concerns | No concerns | Some concerns | Low |
| Cemip:Pembro-chemo | 0 | No concerns | Low risk | No concerns | Some concerns | No concerns | Some concerns | Low |
| Cemip:Serpl-chemo | 0 | No concerns | Low risk | No concerns | Some concerns | No concerns | Some concerns | Low |
| Cemip:Sinti-chemo | 0 | No concerns | Low risk | No concerns | Some concerns | No concerns | Some concerns | Low |
| Cemip:Sugem-chemo | 0 | No concerns | Low risk | No concerns | Some concerns | No concerns | Some concerns | Low |
| Cemip-chemo:Nivo-ipi | 0 | No concerns | Low risk | No concerns | Some concerns | No concerns | Some concerns | Low |
| Cemip-chemo:Pembro | 0 | No concerns | Low risk | No concerns | Some concerns | No concerns | Some concerns | Low |
| Cemip-chemo:Pembro-chemo | 0 | No concerns | Low risk | No concerns | Some concerns | No concerns | Some concerns | Low |
| Cemip-chemo:Pembro-ipi | 0 | No concerns | Low risk | No concerns | Some concerns | No concerns | Some concerns | Low |
| Cemip-chemo:Serpl-chemo | 0 | No concerns | Low risk | No concerns | Some concerns | No concerns | Some concerns | Low |
| Cemip-chemo:Sinti-chemo | 0 | No concerns | Low risk | No concerns | Some concerns | No concerns | Some concerns | Low |
| Cemip-chemo:Sugem-chemo | 0 | No concerns | Low risk | No concerns | Some concerns | No concerns | Some concerns | Low |
| Nivo-ipi:Serpl-chemo | 0 | No concerns | Low risk | No concerns | Some concerns | No concerns | Some concerns | Low |
| Nivo-ipi:Sinti-chemo | 0 | No concerns | Low risk | No concerns | Some concerns | No concerns | Some concerns | Low |
| Nivo-ipi:Sugem-chemo | 0 | No concerns | Low risk | No concerns | No concerns | Some concerns | Some concerns | Low |
| Pembro:Pembro-chemo | 0 | No concerns | Low risk | No concerns | Some concerns | No concerns | Some concerns | Low |
| Pembro:Serpl-chemo | 0 | No concerns | Low risk | No concerns | Some concerns | No concerns | Some concerns | Low |
| Pembro:Sinti-chemo | 0 | No concerns | Low risk | No concerns | Some concerns | No concerns | Some concerns | Low |
| Pembro:Sugem-chemo | 0 | No concerns | Low risk | No concerns | Some concerns | No concerns | Some concerns | Low |
| Pembro-chemo:Pembro-ipi | 0 | No concerns | Low risk | No concerns | Some concerns | No concerns | Some concerns | Low |
| Pembro-chemo:Serpl-chemo | 0 | No concerns | Low risk | No concerns | Some concerns | No concerns | Some concerns | Low |
| Pembro-chemo:Sinti-chemo | 0 | No concerns | Low risk | No concerns | Some concerns | No concerns | Some concerns | Low |
| Pembro-chemo:Sugem-chemo | 0 | No concerns | Low risk | No concerns | Some concerns | No concerns | Some concerns | Low |
| Pembro-ipi:Serpl-chemo | 0 | No concerns | Low risk | No concerns | Some concerns | No concerns | Some concerns | Low |
| Pembro-ipi:Sinti-chemo | 0 | No concerns | Low risk | No concerns | Some concerns | No concerns | Some concerns | Low |
| Pembro-ipi:Sugem-chemo | 0 | No concerns | Low risk | No concerns | Some concerns | No concerns | Some concerns | Low |
| chemo:Pembro-ipi | 0 | No concerns | Low risk | No concerns | Some concerns | No concerns | Some concerns | Low |
| Serpl-chemo:Sinti-chemo | 0 | No concerns | Low risk | No concerns | Some concerns | No concerns | Some concerns | Low |
| Serpl-chemo:Sugem-chemo | 0 | No concerns | Low risk | No concerns | Some concerns | No concerns | Some concerns | Low |
| Sinti-chemo:Sugem-chemo | 0 | No concerns | Low risk | No concerns | Some concerns | No concerns | Some concerns | Low |
| Camre-chemo:chemo | 1 | No concerns | Low risk | No concerns | Some concerns | Some concerns | Some concerns | Very low |
| Cemip:chemo | 1 | Some concerns | Low risk | No concerns | No concerns | Some concerns | Some concerns | Very low |
| chemo:Nivo-ipi | 1 | Some concerns | Low risk | No concerns | Some concerns | Some concerns | Some concerns | Very low |
| chemo:Pembro | 1 | Some concerns | Low risk | No concerns | Some concerns | No concerns | Some concerns | Very low |
| Camre-chemo:Cemip | 0 | Some concerns | Low risk | No concerns | Some concerns | No concerns | Some concerns | Very low |
| Camre-chemo:Nivo-ipi | 0 | Some concerns | Low risk | No concerns | Some concerns | No concerns | Some concerns | Very low |
| Camre-chemo:Pembro | 0 | Some concerns | Low risk | No concerns | Some concerns | No concerns | Some concerns | Very low |
| Cemip:Nivo-ipi | 0 | Some concerns | Low risk | No concerns | Some concerns | No concerns | Some concerns | Very low |
| Cemip:Pembro | 0 | Some concerns | Low risk | No concerns | Some concerns | No concerns | Some concerns | Very low |
| Cemip:Pembro-ipi | 0 | Some concerns | Low risk | No concerns | Some concerns | No concerns | Some concerns | Very low |
| Nivo-ipi:Pembro | 0 | Some concerns | Low risk | No concerns | Some concerns | No concerns | Some concerns | Very low |
| Nivo-ipi:Pembro-chemo | 0 | No concerns | Low risk | No concerns | Some concerns | Some concerns | Some concerns | Very low |
| Nivo-ipi:Pembro-ipi | 0 | Some concerns | Low risk | No concerns | Some concerns | No concerns | Some concerns | Very low |

| **Supplementary Table 6. Certainty of evidence for progression-free survival in NSCLC patients without brain metastases assessed by CINeMA.** | | | | | | | | |
| --- | --- | --- | --- | --- | --- | --- | --- | --- |
| **Comparison** | **Studies(n)** | **Within-study bias** | **Reporting bias** | **Indirectness** | **Imprecision** | **Heterogeneity** | **Incoherence** | **Confidence rating** |
| Camre-chemo:chemo | 1 | No concerns | Low risk | No concerns | No concerns | Some concerns | Some concerns | Low |
| Cemip-chemo:chemo | 1 | No concerns | Low risk | No concerns | No concerns | Some concerns | Some concerns | Low |
| chemo:Pembro-chemo | 1 | No concerns | Low risk | No concerns | No concerns | Some concerns | Some concerns | Low |
| chemo:Serpl-chemo | 1 | No concerns | Low risk | No concerns | No concerns | Some concerns | Some concerns | Low |
| chemo:Sinti-chemo | 1 | No concerns | Low risk | No concerns | No concerns | Some concerns | Some concerns | Low |
| chemo:Sugem-chemo | 1 | No concerns | Low risk | No concerns | No concerns | Some concerns | Some concerns | Low |
| Camre-chemo:Cemip-chemo | 0 | No concerns | Low risk | No concerns | Some concerns | No concerns | Some concerns | Low |
| Camre-chemo:Pembro-ipi | 0 | No concerns | Low risk | No concerns | Some concerns | No concerns | Some concerns | Low |
| Camre-chemo:Serpl-chemo | 0 | No concerns | Low risk | No concerns | Some concerns | No concerns | Some concerns | Low |
| Cemip-chemo:Nivo-ipi | 0 | No concerns | Low risk | No concerns | No concerns | Some concerns | Some concerns | Low |
| Cemip-chemo:Pembro | 0 | No concerns | Low risk | No concerns | Some concerns | No concerns | Some concerns | Low |
| Cemip-chemo:Pembro-ipi | 0 | No concerns | Low risk | No concerns | Some concerns | No concerns | Some concerns | Low |
| Cemip-chemo:Serpl-chemo | 0 | No concerns | Low risk | No concerns | Some concerns | No concerns | Some concerns | Low |
| Cemip-chemo:Sinti-chemo | 0 | No concerns | Low risk | No concerns | Some concerns | No concerns | Some concerns | Low |
| Cemip-chemo:Sugem-chemo | 0 | No concerns | Low risk | No concerns | Some concerns | No concerns | Some concerns | Low |
| Nivo-ipi:Pembro-chemo | 0 | No concerns | Low risk | No concerns | No concerns | Some concerns | Some concerns | Low |
| Nivo-ipi:Serpl-chemo | 0 | No concerns | Low risk | No concerns | No concerns | Some concerns | Some concerns | Low |
| Nivo-ipi:Sinti-chemo | 0 | No concerns | Low risk | No concerns | No concerns | Some concerns | Some concerns | Low |
| Nivo-ipi:Sugem-chemo | 0 | No concerns | Low risk | No concerns | No concerns | Some concerns | Some concerns | Low |
| Pembro:Pembro-chemo | 0 | No concerns | Low risk | No concerns | Some concerns | No concerns | Some concerns | Low |
| Pembro:Serpl-chemo | 0 | No concerns | Low risk | No concerns | Some concerns | No concerns | Some concerns | Low |
| Pembro:Sinti-chemo | 0 | No concerns | Low risk | No concerns | Some concerns | No concerns | Some concerns | Low |
| Pembro:Sugem-chemo | 0 | No concerns | Low risk | No concerns | Some concerns | No concerns | Some concerns | Low |
| Pembro-chemo:Pembro-ipi | 0 | No concerns | Low risk | No concerns | Some concerns | No concerns | Some concerns | Low |
| Pembro-chemo:Serpl-chemo | 0 | No concerns | Low risk | No concerns | Some concerns | No concerns | Some concerns | Low |
| Pembro-chemo:Sinti-chemo | 0 | No concerns | Low risk | No concerns | Some concerns | No concerns | Some concerns | Low |
| Pembro-chemo:Sugem-chemo | 0 | No concerns | Low risk | No concerns | Some concerns | No concerns | Some concerns | Low |
| Pembro-ipi:Serpl-chemo | 0 | No concerns | Low risk | No concerns | Some concerns | No concerns | Some concerns | Low |
| Pembro-ipi:Sinti-chemo | 0 | No concerns | Low risk | No concerns | Some concerns | No concerns | Some concerns | Low |
| Pembro-ipi:Sugem-chemo | 0 | No concerns | Low risk | No concerns | Some concerns | No concerns | Some concerns | Low |
| chemo:Pembro-ipi | 0 | No concerns | Low risk | No concerns | No concerns | Some concerns | Some concerns | Low |
| Serpl-chemo:Sinti-chemo | 0 | No concerns | Low risk | No concerns | Some concerns | No concerns | Some concerns | Low |
| Serpl-chemo:Sugem-chemo | 0 | No concerns | Low risk | No concerns | Some concerns | No concerns | Some concerns | Low |
| Sinti-chemo:Sugem-chemo | 0 | No concerns | Low risk | No concerns | Some concerns | No concerns | Some concerns | Low |
| Cemip:chemo | 1 | Some concerns | Low risk | No concerns | No concerns | Some concerns | Some concerns | Very low |
| chemo:Nivo-ipi | 1 | Some concerns | Low risk | No concerns | No concerns | Some concerns | Some concerns | Very low |
| Pembro:Pembro-ipi | 1 | No concerns | Low risk | No concerns | Some concerns | Some concerns | Some concerns | Very low |
| chemo:Pembro | 1 | Some concerns | Low risk | No concerns | No concerns | Some concerns | Some concerns | Very low |
| Camre-chemo:Cemip | 0 | Some concerns | Low risk | No concerns | Some concerns | Some concerns | Some concerns | Very low |
| Camre-chemo:Nivo-ipi | 0 | Some concerns | Low risk | No concerns | No concerns | Some concerns | Some concerns | Very low |
| Camre-chemo:Pembro | 0 | Some concerns | Low risk | No concerns | Some concerns | No concerns | Some concerns | Very low |
| Camre-chemo:Pembro-chemo | 0 | No concerns | Low risk | No concerns | Some concerns | Some concerns | Some concerns | Very low |
| Camre-chemo:Sinti-chemo | 0 | No concerns | Low risk | No concerns | Some concerns | Some concerns | Some concerns | Very low |
| Camre-chemo:Sugem-chemo | 0 | No concerns | Low risk | No concerns | Some concerns | Some concerns | Some concerns | Very low |
| Cemip:Cemip-chemo | 0 | No concerns | Low risk | No concerns | Some concerns | Some concerns | Some concerns | Very low |
| Cemip:Nivo-ipi | 0 | Some concerns | Low risk | No concerns | Some concerns | Some concerns | Some concerns | Very low |
| Cemip:Pembro | 0 | Some concerns | Low risk | No concerns | Some concerns | Some concerns | Some concerns | Very low |
| Cemip:Pembro-chemo | 0 | No concerns | Low risk | No concerns | Some concerns | Some concerns | Some concerns | Very low |
| Cemip:Pembro-ipi | 0 | Some concerns | Low risk | No concerns | Some concerns | No concerns | Some concerns | Very low |
| Cemip:Serpl-chemo | 0 | No concerns | Low risk | No concerns | Some concerns | Some concerns | Some concerns | Very low |
| Cemip:Sinti-chemo | 0 | No concerns | Low risk | No concerns | Some concerns | Some concerns | Some concerns | Very low |
| Cemip:Sugem-chemo | 0 | No concerns | Low risk | No concerns | Some concerns | Some concerns | Some concerns | Very low |
| Cemip-chemo:Pembro-chemo | 0 | No concerns | Low risk | No concerns | Some concerns | Some concerns | Some concerns | Very low |
| Nivo-ipi:Pembro | 0 | Some concerns | Low risk | No concerns | No concerns | Some concerns | Some concerns | Very low |
| Nivo-ipi:Pembro-ipi | 0 | Some concerns | Low risk | No concerns | Some concerns | Some concerns | Some concerns | Very low |
